# Multi-omics study revealing the complexity and spatial heterogeneity of tumor-infiltrating lymphocytes in primary liver carcinoma

**DOI:** 10.18632/oncotarget.16758

**Published:** 2017-03-31

**Authors:** Lijun Shi, Yang Zhang, Lin Feng, Liming Wang, Weiqi Rong, Fan Wu, Jianxiong Wu, Kaitai Zhang, Shujun Cheng

**Affiliations:** ^1^ State Key Laboratory of Molecular Oncology, Department of Etiology and Carcinogenesis, National Cancer Center/Cancer Hospital, Chinese Academy of Medical Sciences and Peking Union Medical College, Beijing 100021, China; ^2^ Department of Hepatobiliary Surgery, National Cancer Center/Cancer Hospital, Chinese Academy of Medical Sciences and Peking Union Medical College, Beijing 100021, China

**Keywords:** spatial heterogeneity, tumor-infiltrating lymphocytes, gene expression profiling, somatic mutation, next generation sequencing

## Abstract

Intratumoral heterogeneity has been revealed in primary liver carcinoma (PLC). However, spatial heterogeneity of tumor-infiltrating lymphocytes (TILs), which reflects one dimension of a tumor's spatial heterogeneity, and the relationship between TIL diversity, local immune response and mutation burden remain unexplored in PLC. Therefore, we performed immune repertoire sequencing, gene expression profiling analysis and whole-exome sequencing in parallel on five regions of each tumor and on matched adjacent normal tissues and peripheral blood from five PLC patients. A significantly higher cumulative frequency of the top 250 most abundant TIL clones was observed in tumors than in peripheral blood. Besides, overlap rates of T cell receptor (TCR) repertoire for intratumor comparisons, significant higher than those for tumor-adjacent normal tissue comparisons and tumor-blood comparisons, which provide evidence for antigen-driven clonal expansion in PLC. Analysis of the percentage of ubiquitous TCR sequences, regional frequencies of each clone and TIL diversity suggested TIL clones varying between distinct regions of the same tumor, which indicated weak TCR repertoire similarity within a single tumor. Furthermore, correlation analysis revealed that TIL diversity significantly correlated with the expression of immune response genes rather than the mutation load. We conclude that intratumoural T-cell clones are spatially heterogeneous, which can lead to underestimate the immune profile of PLC from a single biopsy sample and may present challenge to adoptive cell therapy using autologous TILs. TIL diversity provides a reasonable explanation for the degree of immune response, implied TIL diversity can serve as a surrogate marker to monitor the effect of immunotherapy.

## INTRODUCTION

Liver cancer is the third and second leading cause of cancer-related death among both men and women in China and worldwide, respectively [1, 2]. Hepatocellular carcinoma accounts for approximately 80% of primary liver carcinoma (PLC) and is the most frequent pathological type [3]. Despite substantial improvements in screening, diagnosis and treatment, the prognosis of PLC patients remains poor. Recent studies have attributed this inconsistency to the high intrahepatic recurrence rate after hepatectomy [4, 5], which is partly due to the naturally heterogeneous properties of PLC.

PLC shows substantial heterogeneity at the genetic level in multiple clinical and genomic studies [6, 7]. However, heterogeneity at the genetic level, for example, mutational heterogeneity, only reflects one dimension of a tumor's spatial heterogeneity, previous studies have also reported intratumor heterogeneity at non-genetic levels, such as methylation and TILs [8, 9]. Repertoire of TIL is generated by the somatic recombination of T cell receptor (TCR) α- and β-chains or γ- and δ-chains [10, 11]. The particular specificity of each T cell clone is determined by highly variable complementary determining region 3 (CDR3), which is generated by the rearrangement of the V, D and J segments at the TCR locus [12]. However, antigen-specific αβ-T cells account for the majority of TILs. Therefore, simultaneously analyzing the thousands of TCR beta chain (TCRβ) CDR3 regions by next-generation sequencing (NGS) can reveal the T cell subclones from various diseases, including autoimmune diseases [13] chronic diseases [14], and even the spatial heterogeneity of the clonal composition of TILs [15–17]. Although several studies have been conducted to assess TIL spatial heterogeneity [15–17], the reported results have been inconsistent. Chen et al. [16] explained this discrepancy by highlighting the nature of different cancer types and the technical limitations of early immune repertoire detection. Originating from livers with a history of chronic hepatocyte inflammation, PLC frequently displays significant heterogeneity between and even within tumors at genetic levels [6, 7]; however, whether spatial heterogeneity occurs in TIL populations in PLC remains unexplored.

TIL diversity, which reflects clonal composition, the potential antigenic recognition spectrum, and the quantity of available T-cell responses in the immune repertoire, is an important component of the comprehensive TIL repertoire. Preliminary evidence emerging from clinical trials of autologous active cellular immunotherapy suggests that sipuleucel-T treatment increases TIL diversity in prostate tumors [18]. The authors ascribe the increase in TIL diversity to the tumor-associated immune response modulated by sipuleucel-T. Although TIL diversity could be applied to explain tumor-associated immune responses, the relationship between TIL diversity and the anti-tumor immune response is still being explored. Moreover, TIL infiltration and clonal expansion is caused by a tumor-antigen-specific T cell response; thus, increasing the number of antigens will enhance the degree of the immune response. Tumor associated antigens such as neoantigens can be created by tumor-specific mutations [19]. Therefore, the total number of mutations theoretically should be associated with the degree of immune response. Previous studies have revealed that a higher mutation burden is predictive of a good response to immune checkpoint blockade therapy in non-small cell lung carcinoma (NSCLC) and melanoma [20, 21]. To understand the association of these components, it is necessary to assess TIL clonal composition and spatial distribution, local molecular phenotype and tumor cell somatic mutations simultaneously using advanced NGS technology.

To this end, we performed TCR repertoire sequencing, gene expression profiling analysis and whole-exome sequencing on multiple tumor samples obtained from five tumors as well as matched adjacent noncancerous liver tissues and peripheral blood from five patients. We found that the TIL repertoire exhibited extensive intratumoral heterogeneity in PLC, and TIL diversity significantly related to the degree of immune response instead of mutation burden. Deciphering the heterogeneity of the TIL repertoire within individual PLCs, the corresponding local molecular phenotype and tumor cell somatic mutations may have important implications for adoptive cell therapy using autologous tumor-antigen-specific TILs.

## RESULTS

### Statistical characteristics of the TCR repertoire sequencing data

**Figure 1:**
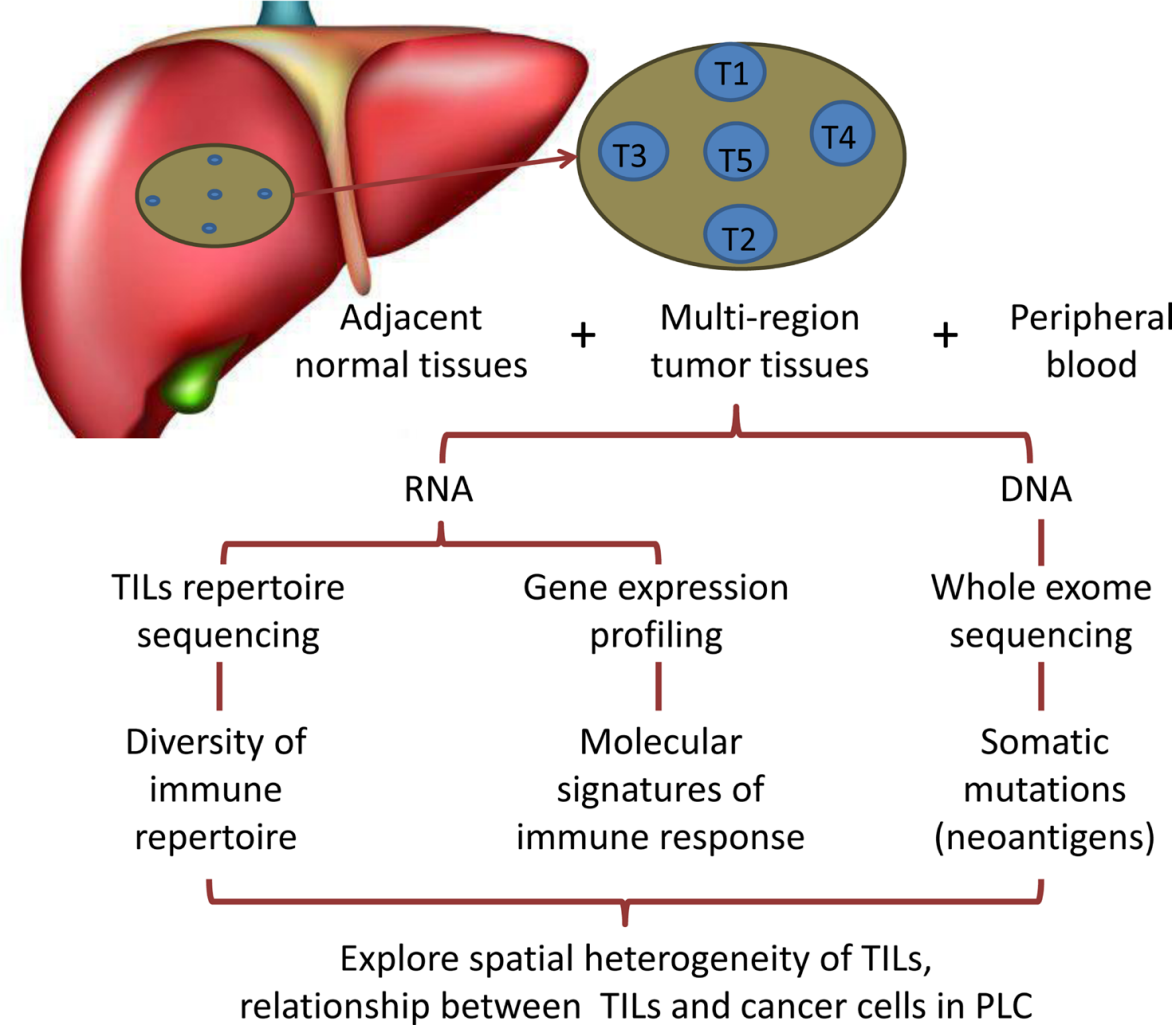
Schematic diagram of study design The brown circle on behalf of the lesions, and the position of blue circles represent spatial distribution of five tumor specimens (T1–T5) from the same tumor.

As shown in the flowchart (Figure 1), the TCRβ CDR3 regions of the T cells were reverse-transcribed and amplified from the total RNA and then sequenced in parallel. TIL repertoire sequencing was performed on 35 specimens from 5 PLC patients, including 25 tumor samples, 5 adjacent noncancerous liver tissues and 5 peripheral blood samples (Table 1). A detailed description of the statistical characteristics of the TCRβ repertoires is included in Supplementary Table 1. The total number of productive TCRβ reads achieved for each sample type from the five patients was 3.65 × 10^6^ to 5.92 × 10^6^ for tumor tissues, 3.94 × 10^6^ to 5.54 × 10^6^ for adjacent noncancerous liver tissues, and 4.14 × 10^6^ to 7.13 × 10^6^ for peripheral blood. The number of unique TCRβ reads was 2.55 × 10^5^ to 9.88 × 10^5^ for tumor tissues, 3.33 × 10^5^ to 7.51 × 10^5^ for adjacent noncancerous liver tissues, and 4.74 × 10^5^ to 1.27 × 10^6^ for peripheral blood, indicating that the average productive TCRβ reads between different tissue types were not significantly different (one-way ANOVA, *p* = 0.650), although average unique TCRβ reads were significantly higher for peripheral blood than for tumor tissues or adjacent normal tissues (one-way ANOVA, blood vs tumor, *p* < 0.001; blood vs noncancerous tissues, *p* = 0.007). This indicated that peripheral blood contains more T cell clone types than tumors or adjacent noncancerous liver tissues.

**Table 1:** Patient characteristics and the number of tissue samples from each of the five patients

Patient ID	Age	Gender	Type^a^	Different-iation^b^	Primary size (cm)	TNM stage	Max diameter(cm)	Location within the tumor
Patient 1	40	male	HCC	M	4 × 4.2 ×4	T1N0M0	4.2	T1: Superior pole, T2: Inferior pole, T3: Left-lateral pole, T4: Right-lateral pole, T5: Centre
Patient 2	65	female	HCC+ICC	L	9 × 7.5 ×3.8	T3N0M0	9	T1: Superior pole, T2: Inferior pole, T3: Left-lateral pole, T4: Right -lateral pole, T5: Centre
Patient 3	37	female	HCC	M	10 × 9 ×6	T2N0M0	10	T1: Superior pole, T2: Inferior pole, T3: Left-lateral pole, T4: Right -lateral pole, T5: Centre
Patient 4	45	male	HCC	M-L	6 × 4.2 ×3.8	T3N0M0	6	T1: Superior pole, T2: Inferior pole, T3: Left-lateral pole, T4: Right -lateral pole, T5: Centre
Patient 5	49	male	HCC	H	8 × 7.5 ×6	T1N0M0	8	T1: Superior pole, T2: Inferior pole, T3: Left-lateral pole, T4: Right -lateral pole, T5: Centre

^a^ HCC: hepatocellular carcinoma, ICC: intrahcpatic cholangiocarcinoma; ^b^ H: high-differentiated, M: moderately-differentiated, L: low-differentiated.

**Figure 2:**
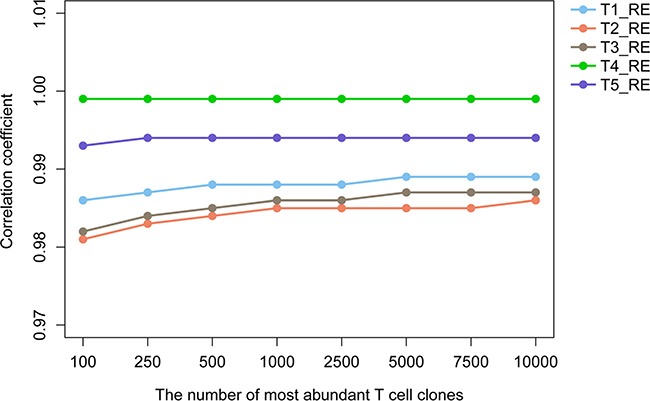
The correlation coefficient of the duplicate sample of each tumor region from patient 4 The y-axis shows the specific numerical of correlation coefficient, x-axis shows the group with various amounts of abundant T cell clones.

In order to evaluate the robustness of our TCR repertoire sequencing technique, the five tumor samples from patient 4 were amplified by PCR and sequenced again (the duplicated samples were recorded as T1_RE, T2_RE, T3_RE, T4_RE and T5_RE, respectively; for example, T1 and T1_RE are duplicate samples of T1). A detailed description of the statistical characteristics of the TCRβ repertoires and the percentages of TCRβ clones with different frequencies are summarized in Supplementary Table 1 and Supplementary Table 2, respectively. We selected the top 100, 250, 500, 1000, 2500, 5000, 7500 and 10000 most abundant T cell clones from the duplicated samples and different tumor samples drawn from the same patient's tumor, respectively. Then, identified the common unique TCRβ reads and calculated the common rate for the duplicate samples of each tumor region from patient 4. Furthermore, we assessed the correlation between the duplicate samples of each tumor region using the productive TCRβ reads of each common T cell clones. As shown in Supplementary Table 3, the average common rates of the duplicate samples of each tumor region from different T cell clone groups was as high as 0.893 (range: 0.842 to 0.950). Besides, the correlation coefficient increased with the increase number of T cell clones and always greater than 0.980 (Figure 2). This indicated that our TCR repertoire sequencing technique exhibits an excellent stability. The duplicated samples T1_RE, T2_RE, T3_RE, T4_RE and T5_RE were not included in the subsequent analysis.

### Differences in TCR repertoire between tumor tissue, adjacent normal tissue and peripheral blood

**Figure 3:**
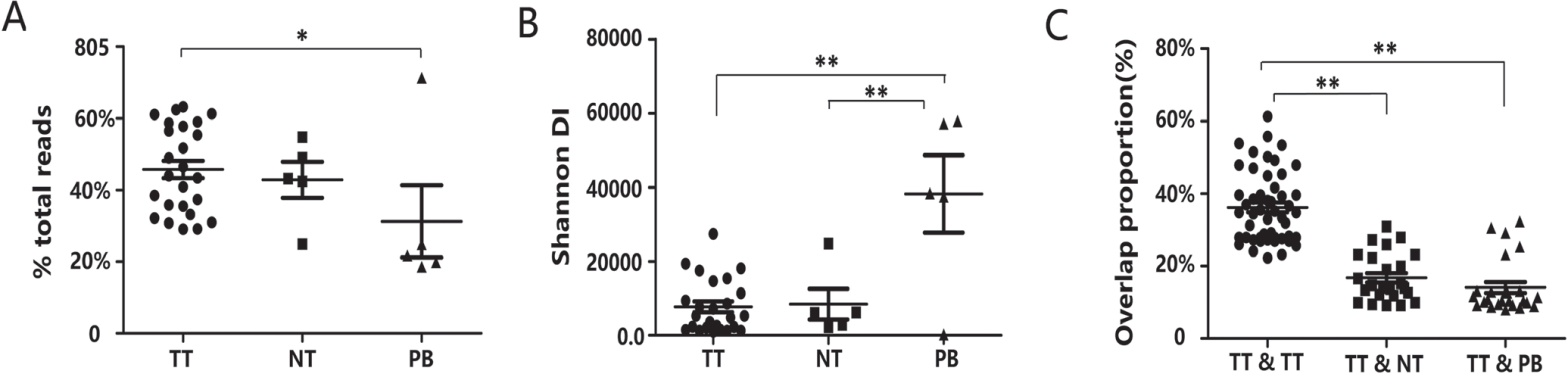
The distribution characteristics of the TCRβ repertoires in tumor tissues, adjacent normal tissues and peripheral blood (**A**) The cumulative frequency of the TOP250 in tumor tissues, adjacent normal tissues and peripheral blood. Data points represent the cumulative frequency of the TOP250 of each sample from five patients, and bars depict the mean (± SEM) of the groups. Differences between groups were compared using one-way ANOVA. *P = 0.039. TT, NT and PB represent tumor tissues, adjacent normal tissues and peripheral blood, respectively. (**B**) Data show the distribution of TCR diversity by measuring the shannonDI. Each dot represents the shannonDI of each sample, and bars show the mean (± SEM) of the groups. Differences between groups were compared using one-way ANOVA. **P < 0.001. (**C**) Data show the overlap of clonotypes between sample groups, TT and TT, TT and NT, TT and PB. Each dot represents the overlap rate between any two samples, and bars show the mean (± SEM) of the groups. Differences between groups were compared using one-way ANOVA. **P < 0.001.

To overview the composition of TCRβ repertoires, the percentages of TCRβ clones with different frequencies are summarized in Supplementary Table 2. We observed an obviously skewed distribution in the TCRβ clone frequency. This finding implied that only a small fraction of T cells was highly expanded. Then, to further explore the configuration of the T cell repertoire, the cumulative frequency of the top 250 most abundant T cell clones (called TOP250 hereafter) from each sample were calculated and plotted (Supplementary Figure 1). The results showed that the average cumulative frequencies for the TOP250 were 45.74% (range: 29.13% to 63.24%) in tumors, 42.86% (range: 24.90% to 54.77%) in adjacent noncancerous liver tissues and 31.25% (range: 18.55% to 71.31%) in peripheral blood. The cumulative frequencies of the TOP250 were significantly higher in tumors than in peripheral blood (one-way ANOVA, *p* = 0.039, Figure 3A, Supplementary Table 1), although there was no significant difference between tumors and adjacent noncancerous liver tissues. This indicated that T cell clones were highly expanded in tumor tissues, which agrees with a previous study [22]. In all three sample types, the TOP250 contained one-quarter or more of the total TCR repertoire, which suggests that the entire repertoire had been dominated by a tiny fraction of clones. Thus, to reduce the sampling bias of rare clones, only the TOP250 in each sample was included in the subsequent analysis except for the TCR diversity analysis.

Moreover, we analyzed the differences among tumor tissue, adjacent normal tissue and peripheral blood from five patients based on the diversity of T cell clones. On average, TIL diversity was 7689.49 (range: 949.64 to 27406.56) in tumor tissues, 8416.05 (range: 2215.72 to 24790.03) in adjacent normal tissues, and 38232.71 (range: 234.52 to 57895.63) in peripheral blood samples. A significantly lower TIL diversity was observed in tumor samples and adjacent normal tissues than in peripheral blood samples (one-way ANOVA, tumor tissues vs peripheral blood, *p* < 0.001; adjacent normal tissues vs peripheral blood, *p* < 0.001, Figure 3B), although no significant difference was observed between tumor tissues and adjacent normal tissue (one-way ANOVA, *p* = 0.892). The results supported the existence of differences in immune repertoire between tumor tissue and adjacent normal tissues or peripheral blood and indicated that the TCRβ repertoires of PLC tissues were less clonotypes than in matched peripheral blood.

**Figure 4:**
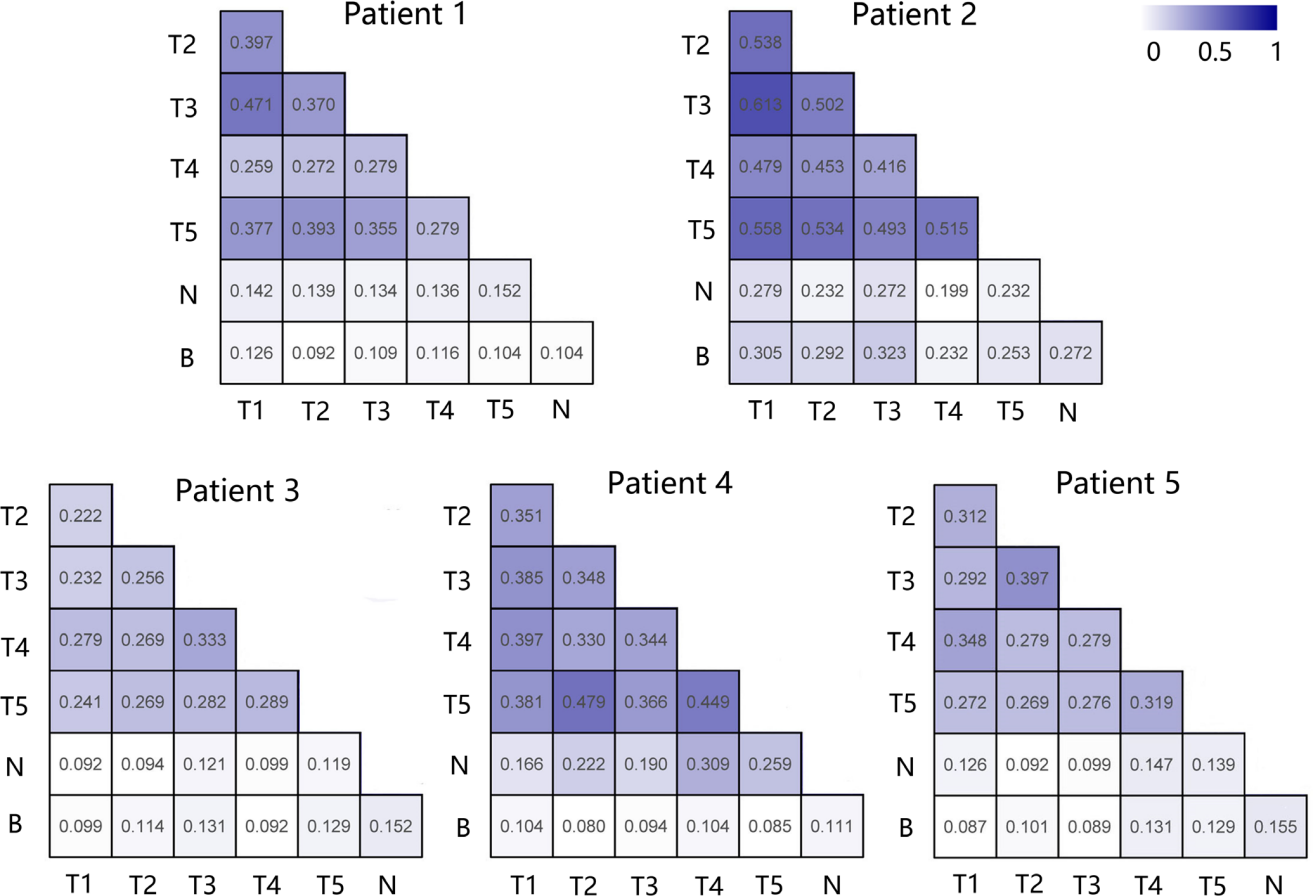
Comparison of the pairwise overlap of TCRβ repertoire between different samples of each patient For each patient we computed pairwise overlaps among all samples, the high overlap rate obtain a darker shade of blue in the heat map. Sample names T, N and B represent tumor tissues, adjacent normal tissues and peripheral blood, respectively.

Additionally, to further decipher the dissimilarity of the immune repertoires in tumor tissue, adjacent noncancerous liver tissues and peripheral blood for each patient, we generated heat maps to illustrate the pairwise sequence overlap for each PLC patient. The average pairwise sequence overlap rates from five PLC patients was 36.19% (range: 22.25% to 61.29%) for tumor-tumor pairs, 16.76% (range: 9.17% to 30.89%) for tumor-adjacent normal tissue pairs, and 14.08% (range: 7.99% to 32.28%) for tumor-peripheral blood pairs. There was significantly higher overlap rates between tumor tissues than those between the tumor and other tissues (one-way ANOVA, tumor-tumor pairs vs tumor-adjacent normal tissue pairs, *p* < 0.001; tumor-tumor pairs vs tumor-peripheral blood pairs, *p* < 0.001, Figure 3C). The results further suggested that the TIL repertoire is more similar between tissues of the same tumor than that for tumor tissues compared to adjacent noncancerous tissue or peripheral blood (Figure 4), which agrees with findings published in esophageal cancer patients [16]. In order to verify the stability of our results, we re-assessed the pairwise overlap including only the 100 T cell clones found with the highest regional frequency across each region of the five tumors. As shown in Supplementary Figure 2A, the TIL repertoire is still more similar between tissues of the same tumor than that for tumor tissues compared to adjacent noncancerous tissue or peripheral blood. Collectively, T cell clones which highly expand in PLC could be related to the tumor.

### Spatial heterogeneity of TCR repertoire within a single tumor

**Figure 5:**
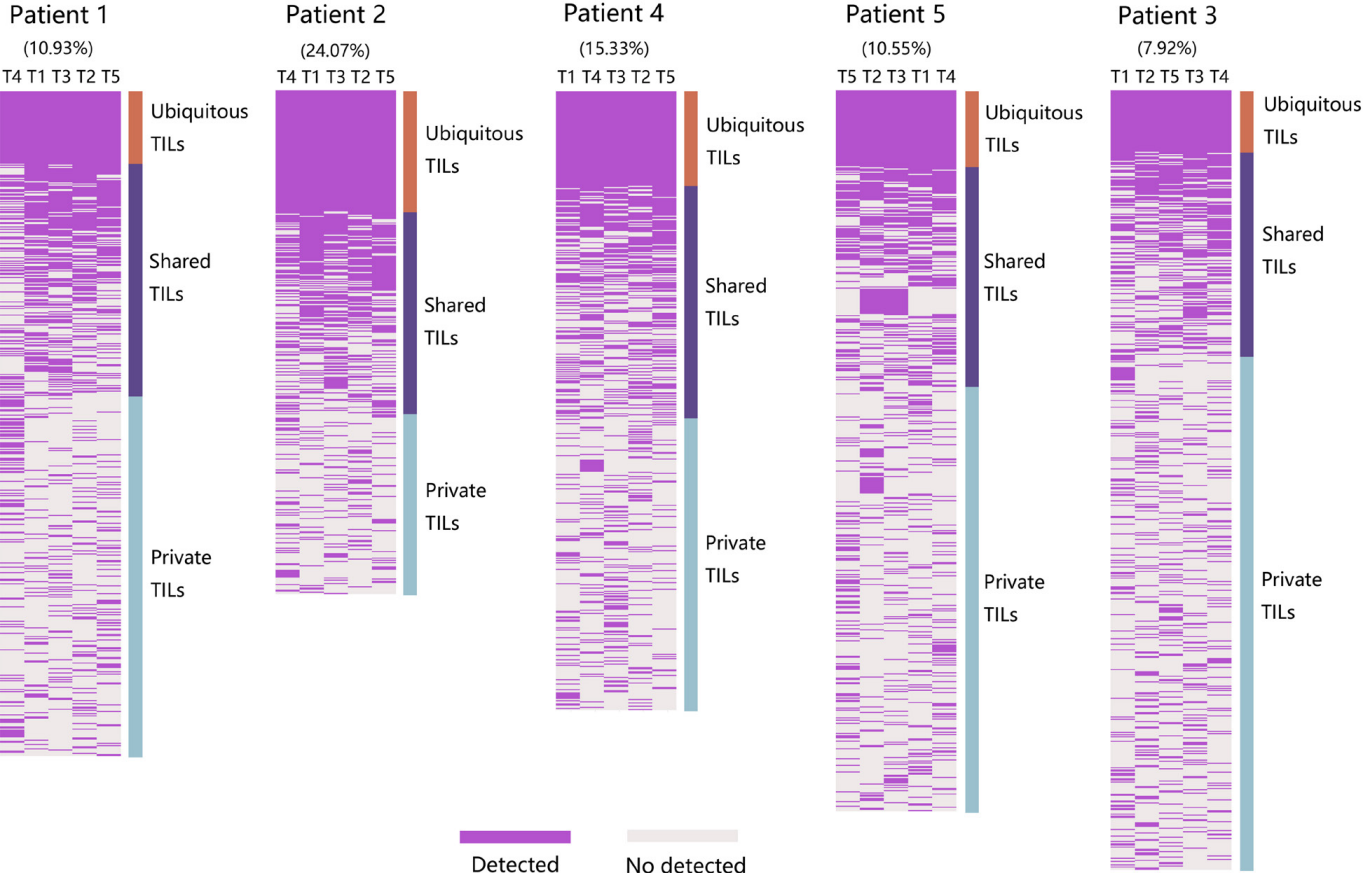
Spatial heterogeneity of TIL clones in five PLC patients Heat maps show the regional distribution of TOP250 from all tumor samples of each patient. T cell clones identified in its original regions showed purple, otherwise light grey. Column close to heat map show three categories of TIL populations: TILs present in all regions were defined as ubiquitous (salmon), in more than one but not all regions were considered as shared (modena) and in one region was regarded as private (light-blue). Both shared and private TILs were heterogeneous. Patient identification is showed on the top of figure. Then, the percentage of ubiquitous TIL clones of each patient is indicated. Next, lesion names in the form of regional identification.

To dissect the degree of intratumoral TIL heterogeneity in PLC, we further analyzed the TCR repertoires from multiple regions of each tumor and used the ubiquitous rate to measure the degree. The clones present in all the regions from each tumor were defined as ubiquitous; clones present in more than one but not all regions were considered shared; and those found only in one region were private. Both shared and private TILs were heterogeneous. As shown in Figure 5, the percentage of ubiquitous sequences in the TOP250 ranged from 7.92% to 24.07% (median 10.93%), whereas 75.93–92.08% (median 89.07%) of the sequences were heterogeneous, indicating that TIL populations are spatially heterogeneous in PLCs and that the degree of spatial heterogeneity showed obvious differences between patients. Spatial heterogeneity of TIL populations was still remains when including only the 100 most abundant T cell clones (Supplementary Figure 2B).

**Figure 6:**
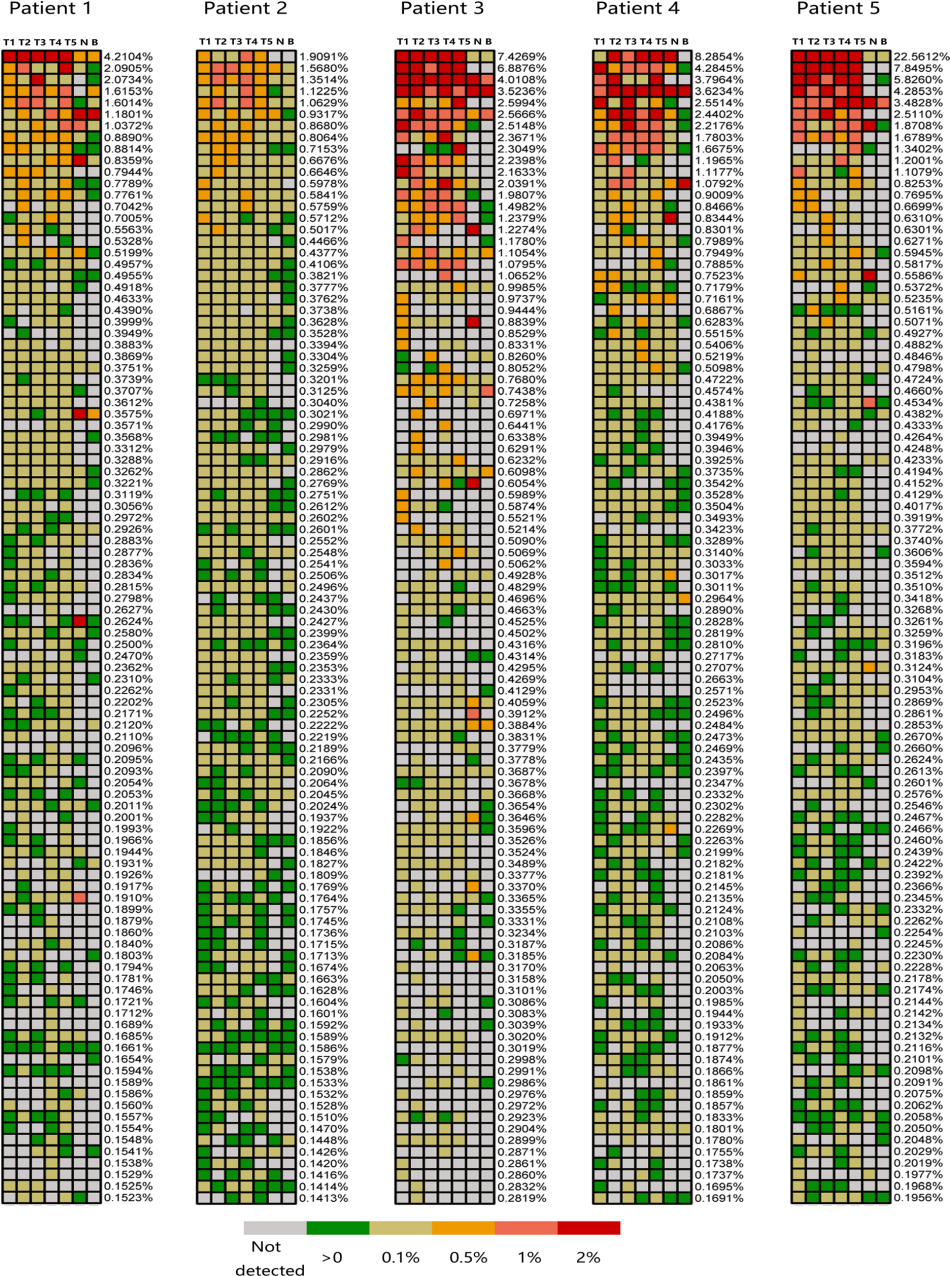
Regional frequencies of the 100 most abundant T cell clones in different samples of the five PLC patients The 100 most abundant TIL clones showed in heat maps identified as the highest regional frequencies throughout five regions of each tumor. Frequencies that listed on the right of each heat map represent the corresponding T cell clones. The color of cell check indicates different frequency of T cell clone, the corresponding relationship between color and clone abundances are indicated by figure legend presents on the bottom of figure. Patient identifications and lesion names are showed on the top of figure.

Along with the T cell clonal population, the frequency of each oligoclone is important for the immune response. Thus, the frequencies of each clone detected in all regions of each tumor were also required to assess spatial heterogeneity within a single tumor. We used the heat maps to demonstrate the regional abundance of each T cell clone in each tumor. Only the TOP250 present in each tumor region were included in the analysis. The TIL population was not identical among the tumor regions, and there were more than 250 unique clones from the tumor regions of each patient. To facilitate the generation of heat maps, we used the top 100 highly expanded T cell clones from all the tumor tissues from each patient (Figure 6). Heat maps of the 100 most frequent clones from all the tumor regions from each patient demonstrated that different tumor regions predominantly harbor distinct T cell clones. Even the ubiquitous T cell clones show regional abundance with obvious differences in certain tumor regions. Most of the highly expanded T cell clones present in tumor samples were low in abundance or did not exist in matched noncancerous liver tissues and peripheral blood, revealing that only a small fraction of T cells present in the tumor samples circulate throughout the tumor within the blood and implying that the majority of T cell clones detected with high frequency in the tumor tissues were related to tumor-associated antigens. Additionally, to verify the stability of this result, only the 100 most abundant T cell clones present in each tumor region were included in analysis according to the foregoing method. As shown in Supplementary Figure 2C, spatial heterogeneity of TIL populations in PLC was still remains and that different TIL clones could be dominant in different tumor regions, which indicated the results exhibit an excellent stability in this study.

**Figure 7:**
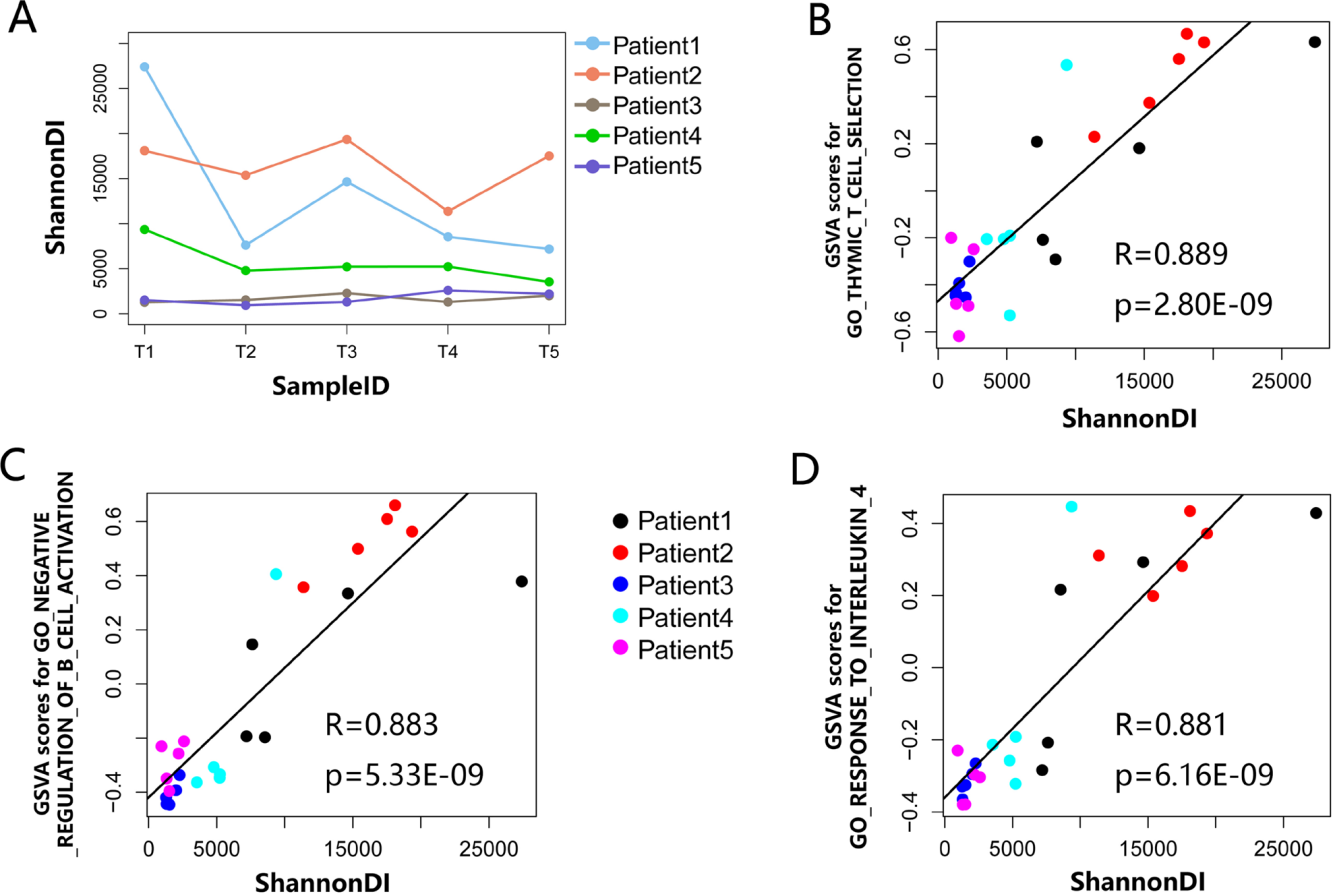
The relationship between local TCR clone diversity and the immune status (**A**) The ShannonDI for each tumor sample of five PLC patients. (**B**–**D**) Correlations between ShannonDI values and GSVA score of three GO gene sets list in Table 2 that achieved the largest correlation coefficient in the tumor samples of five PLC patients. The best-fit lines are indicated on each panel, each dot represents an individual tumor region.

Furthermore, we further investigated the spatial heterogeneity of the TCR repertoire by analyzing TIL diversity within a tumor. The median ShannonDI value was 8544.03 (range: 7187.40 to 27406.56), 17516.28 (range: 11369.95 to 19349.52), 1534.50 (range: 1278.49 to 2281.84), 5218.94 (range: 3541.83 to 9356.73) and 1521.37 (range: 949.64 to 2593.43) for patient 1 to patient 5, respectively. This indicated that TIL diversity fluctuated considerably in different sites of the same tumor (Figure 7A), which provides additional evidence of the spatial heterogeneity of TILs.

### TCR diversity is associated with expression level of immune response genes rather than mutation burden

Baseline TCR diversity has been linked to prognosis and response to immunotherapy. However, whether TCR diversity can serve as a surrogate marker for the antigen-specific T-cell immune response remains unexplored. To this end, mRNA transcriptome profiling of the 25 tumor samples from five PLC patients was performed using Agilent microarrays to evaluate the correlation between TCRβ repertoire diversity in the tumor microenvironment and the local molecular phenotype. To collect information reflecting the molecular phenotype in a more robust manner from the mRNA transcriptome profiling data, the GSVA scores for the GO gene sets provided by the Molecular Signatures Database were calculated for each of the 25 tumor samples to evaluate their activity. Then, correlation analysis was conducted to assess the relationship between TCR clonal diversity and the activity of the gene sets. Ten GO gene sets with the largest correlation coefficients are listed in Table 2, and we found six gene sets in Table 2 that were associated with immune response, suggesting a significant association between TCR clonal diversity and the anti-tumor immune response. Figure 7B–7D shows the relationship between the ShannonDI, which represents TIL diversity, and the three GO gene sets that achieved the largest correlation coefficient, respectively (Pearson's correlation, all *p* < 0.001).

**Table 2:** The ten gene sets having the largest correlation coefficient

GO terms^a^	R	*P* Value
**GO_THYMIC_T_CELL_SELECTION**	0.889	2.80E-09
**GO_NEGATIVE_REGULATION_OF_B_ CELL_ACTIVATION**	0.883	5.33E-09
**GO_RESPONSE_TO_INTERLEUKIN_4**	0.881	6.16E-09
GO_POSITIVE_REGULATION_OF_ACTIN_FILAMENT_BUNDLE_ASSEMBLY	0.878	8.29E-09
**GO_CELLULAR_RESPONSE_TO_INTERLEUKIN_4**	0.868	1.85E-08
**GO_T_CELL_DIFFERENTIATION_IN_THYMUS**	0.868	1.85E-08
**GO_THYMOCYTE_AGGREGATION**	0.868	1.94E-08
GO_NEGATIVE_REGULATION_OF_KINASE_ACTIVITY	0.864	2.57E-08
GO_POSITIVE_REGULATION_OF_TRANSCRIPTION_FROM_RNA_POLYMERASE_II_PROMOTER	0.864	2.66E-08
GO_REGULATION_OF_ACTIN_FILAMENT_ BUNDLE_ASSEMBLY	0.860	3.69E-08

^a^ GO terms which relate to immune response are indicated in bold.

Non-synonymous mutations in cancer cells are known neoantigen sources that can trigger a T cell-dependent immune response. Previous studies by Rooney and colleagues have suggested a significant correlation between mutation burden and cytolytic activity index in a pan-cancer context [23]. Synonymous mutations do not yield neoantigens, and the correlation between non-synonymous mutational burden and immune activity is important to investigate. Whole-exome sequencing resulted in a mean coverage of 126.87 reads (Supplementary Table 4) in our study. We identified 430 non-synonymous mutations and mapped their regional distributions across multiple regions of each tumor (Supplementary Table 5). We selected 75, 70, 52, 56 and 80 non-synonymous mutations from patient 1 to patient 5, respectively, to validate the use of Sanger sequencing (Supplementary Figure 3). For each PLC patient, routine validation was performed in T1-T5. On average, 97.03% of the mutations were specifically validated in the regions in which they were initially detected (Supplementary Table 6). We discarded mutations which failed to validate and then investigated the relationship between TIL diversity and the number of remaining non-synonymous mutations. No significant correlation between TIL diversity, represented by the ShannonDI within the tumor microenvironment, and the corresponding numbers of somatic non-synonymous mutations (Spearman's correlation, *p* = 0.163) was observed. Overall, our results associate the expression of immune response genes rather than mutational load with TIL diversity in PLC.

## DISCUSSION

To our knowledge, this is the first study to evaluate the intratumoral spatial heterogeneity of TIL populations and the association between TILs and cancer cells in primary PLC patients. Accumulating evidence has supported the idea that the tumor micro-environment [24–26], including TILs [15, 16], exhibits extensive spatial heterogeneity. In our study, we conducted next-generation TCR sequencing to elucidate the spatial distribution of TIL populations in PLC. Analysis of the pairwise sequence overlap of TCRβ repertoire revealed that intratumoral TIL populations are more similar than those between tumor and adjacent tissues or peripheral blood, which is consistent with recent findings in renal cell carcinomas [15] and esophageal squamous cell carcinomas [16]. This result indicated that T cell clones spatially confined in the tumor micro-environment might be tumor-specific. In addition, the cumulative frequencies of the TOP250 were significantly higher in tumors than in peripheral blood, indicating that tumor-related T cell clones were highly expanded in tumor tissues. This suggests that autologous tumor-antigen-specific TILs may be an effective agent for PLC patients as for metastatic colorectal cancer [27]. Moreover, the tumor-peripheral blood overlap revealed that 7.99% to 32.28% of the αβ-T cells in peripheral blood might be tumor-specific, which would also provide a potential non-invasive method to monitor the intratumoral environment during clinical trials.

Regarding the TIL spatial distribution within a tumor, our study demonstrated that only approximately ten percent (7.92–24.07%) of the T cell clones were shared among the five analyzed biopsy sites for each patient. This suggests that distinct tumor regions harbor different T cell clones, indicates TIL populations present in PLC exhibit extensive spatial heterogeneity. Even for the ubiquitous clones, regional frequencies show obvious discrepancies. Furthermore, analysis of TIL diversity revealed that it varies between distinct regions of the same PLC, further demonstrating that intratumoral TIL repertoires were spatially heterogeneous. Similar findings have been reported in esophageal squamous cell carcinomas [16]. This suggests that single tumor-biopsy specimens reveal only a minority of the TIL signature present in an entire tumor, and it's possible to overestimate the effects of adoptive cell therapy using autologous TILs that obtained from single tumor-biopsy specimens.

Immune repertoire diversity is a fundamental determinant of the immune system competence [28]. Diversity of TILs is closely related to patient prognosis and treatment effectiveness for different diseases [29, 30]. Previous studies have reported that low TIL diversity at baseline suggests tumor progression and non-functional TILs [31]. In our study, association analysis revealed that the diversity of TCRβ repertoires was significantly correlated with the T cell-mediated immune response, which suggests TIL diversity can serve as a surrogate marker for the antigen-specific T-cell immune response, indicates that increasing TIL diversity would lead to more effective killing of cancer cells during antitumor therapy. For example, higher GSVA score of GO term “response to interleukin 4”, higher TIL diversity, which indicated the greater interleukin 4 was secreted by activated T lymphocytes. Recent studies revealed that interleukin 4 can stimulate growth of B and T lymphocytes, enhance cytotoxic T lymphocyte activity and promote growth of TILs [32–34]. Besides, Soydinc and colleagues reported that serum IL-4 levels decreased following chemotherapy [35]. Thus, higher TIL diversity at baseline suggests more pre-existing tumor-related T cells in the tumor microenvironment, which predicts a better response to chemotherapy and clinical outcomes. However, as our study is a cross-sectional study, the prognostic information for the PLC patients is unknown. Whether TIL diversity predicts PLC patient clinical outcomes must be further analyzed in a longitudinal, large-scale study.

Clinical trials of immune checkpoint blockade therapy in melanoma and NSCLC indicated that high mutational loads might predict good responses [20, 21]. A higher number of non-synonymous mutations are expected to result in greater numbers of neoantigen [36]. Neoantigens-normally absent from the human genome-that are presented by the major histocompatibility complex (MHC) on the surface of tumor cells can elicit T cell immune responses, which facilitates rejection of the tumor cells by the immune system [19]. Thus, each additional mutation increases the odds of identifying a rejection epitope, and the burden of non-synonymous mutations has been considered as biomarker for anti-tumor immunity. In our study, non-synonymous somatic mutations in combination with TIL diversity in 25 tumor samples were simultaneously analyzed. We observed no correlation between the degree of anti-tumor immunity presented by TIL diversity and the corresponding numbers of non-synonymous somatic mutations. However, the lack of association between TIL diversity and mutational burden does not rule out neoantigens as a driver of lymphocyte activation. A large proportion of non-synonymous mutations produce antigens with low or no immunogenicity. Although a minority of non-synonymous mutations yields high immunogenicity neoantigens, these are obscured in the surrounding mutational profile, which reduces the “visibility” of these mutations to investigators attempting to distinguish them from non-synonymous mutations. Furthermore, some low frequency mutations that encode immunogenicity neoantigens might be neglected due to the lower sequencing depth.

Antigen-specific αβ-T cells are generated by somatic recombination of the TCR α- and β-chains. In this study, we used the TCRβ chains to analysis the diversity of TCR repertoires, which is likely to underestimate the diversity of TIL repertoires because TCRs sharing a common TCR β-chain may have different TCR α-chains. Besides, TCRβ chain sequencing allows analysing the clonal expansion of intratumoral and peripheral T cells, but we cannot compare the clonal expansion obtained from distinctly subtypes of T cells, such as CD4+ T cells and CD8+ T cells. Only when combined with cell sorting technique by cell surface marker, TCRβ chain sequencing makes it possible for us to evaluate the differences between the TCR repertoires of the T cell subsets. Lastly, we did not consider the MHC binding ability of the mutated proteins because we could not find a reliable method. Much effort has been dedicated to solve these limitations in the future.

In summary, using TCR repertoire sequencing, we provide a comprehensive characterization and report the spatial heterogeneity of the PLC-infiltrating T cells from five PLC patients. Furthermore, a synthetic analysis of the relationship between TILs and cancer cells connected the expression level of immune response genes, rather than mutational load, with TIL diversity. In view of the modest sample sizes, the findings should be further validated in a large-scale study.

## MATERIALS AND METHODS

### Patient samples

Fresh tumor specimens as well as matched adjacent noncancerous liver tissues and peripheral blood were obtained from five patients diagnosed with PLC by histopathology and treated with surgical resection at the National Cancer Center/Hospital of the Chinese Academy of Medical Sciences and Peking Union Medical College from November 2015 to March 2016. The spatial distribution of five tumor tissues from each PLC patient is shown in Supplementary Figure 4. Different regions of the tumor specimens and matched adjacent normal tissue from each patient were bisected, and one tissue sample was processed with RNAlater (Invitrogen, cat. AM7021) and stored at −80°C; the other sample was sent for histological verification. Four milliliters of blood was collected into a K2EDTA tube (BD, cat. 367844) prior to surgery. HE staining showed that all the PLC samples contained more than 90% viable tumor cells. All the patients with chronic HBV infection were treatment naïve, and informed consent was obtained from each patient for this research. The clinical characteristics of all of the individuals are presented in Table 1. This study was approved by the Ethics Committee of the National Cancer Center/Hospital of the Chinese Academy of Medical Sciences and Peking Union Medical College.

### TCR sequencing and data analysis

Genomic DNA and total RNA were extracted from each specimen (20-30 mg) simultaneously using the AllPrep DNA/RNA mini kit (Qiagen, cat. 80204). Germ-line DNA and blood cell RNA were extracted from whole blood using a DNeasy Blood & Tissue kit (Qiagen, cat. 69504) and TRIzol reagent (Thermo Fisher, cat. 15596018), respectively. Subsequently, total RNA was used to prepare TCR libraries. A series of three previously described nested PCR reactions [37] were performed to obtain a TCR repertoire using the primers shown in Supplementary Table 7; the ARM-PCR procedure [28] was slightly modified for this assay. Primer TRBC Ro (Supplementary Table 7) and ProtoScript II Reverse Transcriptase (NEB, cat. no. M0368X) were used to synthesize TCRB-specific cDNA from 500 ng of total RNA. For the first PCR reaction, pre-amplification was performed with a multiplex PCR 5x Master Mix (NEB, cat. no. M0284S) using multiple Vβ region primers (TRBV1Fo - TRBV30Fo, Supplementary Table 7) and a Cβ region primer (TRBCRo primer) in a 25 μl reaction based on the following cycling conditions: 95°C for 3 min; 95°C for 30 sec, 60°C for 2 min, 68°C for 1 min × 10 cycles; 68°C for 5 min; and 4°C. Then, 2 μl of the first reaction product was used as the template for the next 25 μl PCR with NEB multiplex PCR master mix and TRBV1Fi - TRBV30Fi and TRBCRi primers (Supplementary Table 7). The cycling conditions were identical to the first step. The goal of the final PCR was to incorporate barcodes and enable sequencing on the Illumina HiSeq 2500 platform (pair-end 250). Two microliters of the second product was used as a template for 50 μl PCR using Deep VentR (exo-) DNA polymerase (NEB, cat. no. M0259S) with the primers SuperF and SuperR (Supplementary Table 7) according to the following cycling conditions: 94°C for 3 min; 94°C for 30 sec, 72°C for 1 min × 25 cycles; 72°C for 5 min; and 4°C. Next, DNA ranging from 200-500 bp was separated and purified with a QIAquick gel extraction kit (Qiagen, cat. no. 28706), and the amplicons were sequenced. Clean data were acquired using Trimmomatic v0.33. Then, FLASH v1.2.11 was used to merge the paired reads to obtain the complete CDR3 region sequence for TCRb. MiGEC v1.2.1b was used to assign the rearranged mRNA sequences to their germ-line V, D, and J counterparts. Subsequently, we applied VDJ tools v1.0.0 as a post-alignment analysis tool to analyze the basic TCR clone statistics and T cell clonal diversity (calculation of Shannon-Wiener diversity index) [38–40]. All raw data of TCR sequencing are available from the SRA database (accession number SRP094909).

### Messenger RNA microarray analysis

RNA samples from five PLC patients were analyzed using the Agilent 4 × 44 K Whole Human Genome Oligo microarrays (Agilent, cat.no G4112A). The labeling, hybridization, and washing were performed according to the manufacturer's instructions. Then, the slides were scanned using an Agilent SureScan Microarray Scanner (G2600D) and extracted with Agilent Feature Extraction Software v10.5.1.1.

The data were normalized using the quantile method from the “limma” R package. The raw and normalized PLC data were submitted to the GEO database with the accession number GSE92528.

### Whole-exome sequencing and somatic mutation calling

DNA of tumor samples were normalized to 1 μg and sheared to 150–200 bp according to the manufacturer's protocol for Agilent Sure Select Human All Exon V6 (Agilent, cat. no. 5190-8863). Then, a Paired-End DNA Sample Prep Kit (Illumina, cat. no. PE-102-1002) was used to repair the end of the enriched DNA fragments and ligate adaptors to them. The capture libraries were quality checked and sequenced on an Illumina HiSeq 2000 instrument (Illumina, San Diego). For each sample, the average effective read depth required a minimum of 100-fold. Illumina CASAVA v1.7 software with default parameters was used to process the raw image files, and the sequences for each sample were generated as 100-bp paired-end reads. For the raw data, we first removed the adapter sequence. In addition, the low-quality reads were discarded. Then, the cleaned short paired-end reads were aligned to hg19 (UCSC) using the Burrows-Wheeler Aligner (BWA v0.7.12) with default parameters. The aligned SAM files were converted to BAM format and sorted. Picard tools (broadinstitute.github.io/picard/) v1.119 was used to remove PCR duplications. Next, the data were processed for local InDel realignment and base quality recalibration using the typical GATK workflow (Genome Analysis Toolkit v3.4-46). For somatic mutation calling, we used VarScan v2.3.9. The following criteria were used to further filter the calls for each variant position: 1) at least 10× coverage in the germline sample of each patient with zero non-reference reads; 2) at least 10x total coverage in tumor samples with over 2× mutation coverage; 3) the variant position had to be annotated as exonic by ANNOVAR v2015 Jun16, and multiple variants located in a genomic interval shorter than 100 bp within the identical site were discarded. All of the raw NGS data have been deposited in the SRA database (accession number SRP092464).

### Additional statistical analysis

Pairwise overlap rate was used to explore the similarity of TCRβ repertoires between two samples and was calculated as follows: n/U_ab_, in which a and b represent two different samples, n is the unique TCRβ CDR3 sequences present in both samples, and U represents the total number of unique sequencing reads in a and b. The Shannon-Wiener diversity index (ShannonDI) was used to quantify the T cell clonal diversity in this study. The Gene Set Variation Analysis (GSVA [41]) program was used for expression profiling of the multi-region tumor samples to assess the underlying activity variation for all of the GO gene sets provided by the Molecular Signatures Database v5.2. For correlation analysis, Pearson's or Spearman's correlation was performed using the R packages “stat.” Moreover, one-way analysis of variance (ANOVA) was used to analyze normally distributed data. *p*-values < 0.05 were considered statistically significant. These analyses were performed using SPSS v16.0 (SPSS Inc., Chicago, IL, USA).

## SUPPLEMENTARY MATERIALS FIGURES AND TABLES







## Abbreviations

TILs: tumor-infiltrating lymphocytes
PLC: primary liver carcinoma
TCR: T cell receptor
CDR3: complementary determining region 3
TCRβ: TCR beta chain
NGS: next-generation sequencing
NSCLC: non-small cell lung carcinoma
TOP250: the top 250 most abundant T cell clones
MHC: major histocompatibility complex
