# Supplementary material for: Multi-omics study revealing the complexity and spatial heterogeneity of tumor-infiltrating lymphocytes in primary liver carcinoma
Source: Oncotarget. 2017 Mar 31;8(21):34844–57. doi: 10.18632/oncotarget.16758 (PMC5471016; doi:10.18632/oncotarget.16758)
Supplement: Supplementary file 1 [file oncotarget-08-34844-s001.pdf]

Multi-omics study revealing the complexity and spatial heterogeneity of tumor-infiltrating lymphocytes in primary liver carcinoma

Supplementary Materials

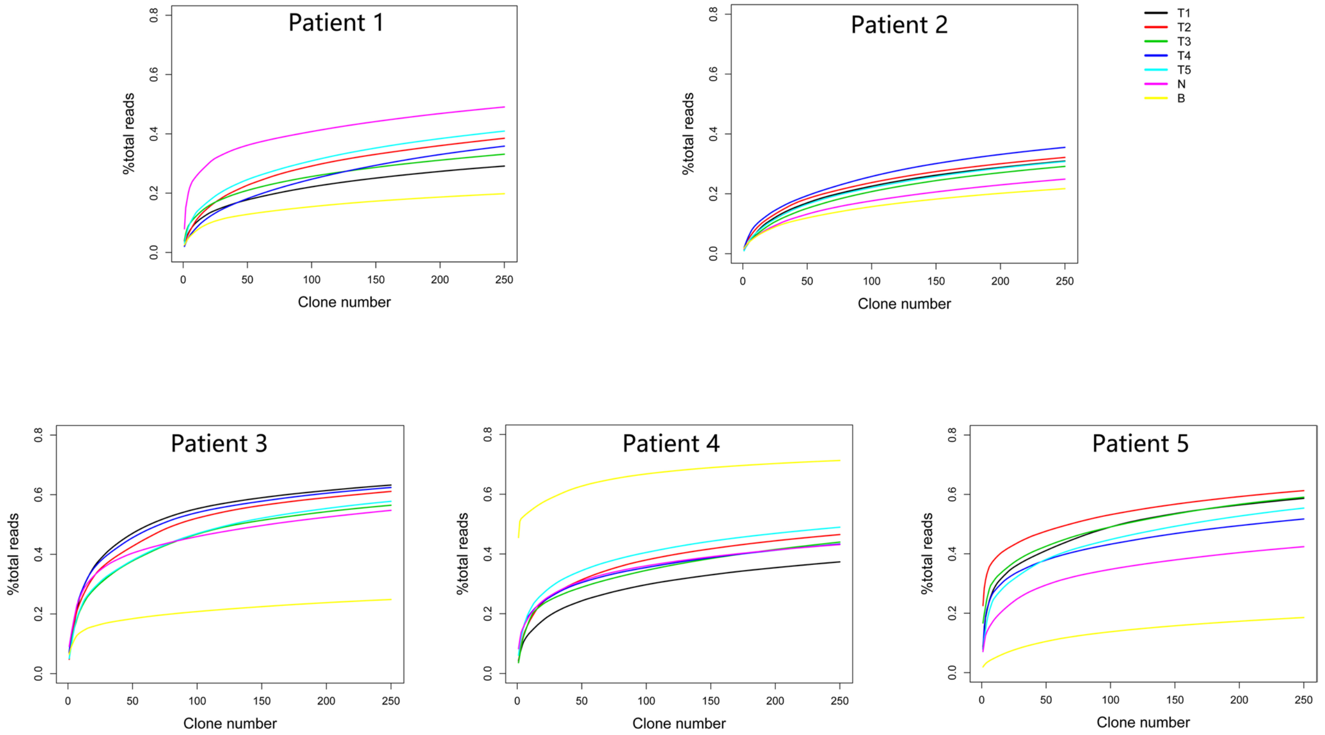

Supplementary Figure 1: The cumulative size of the TOP250 in five PLC patients. The x-axis depicts the number of clones included (starting from the most expanded clones). The y-axis shows the percentage of TCRβ sequences that are covered by the included clones.

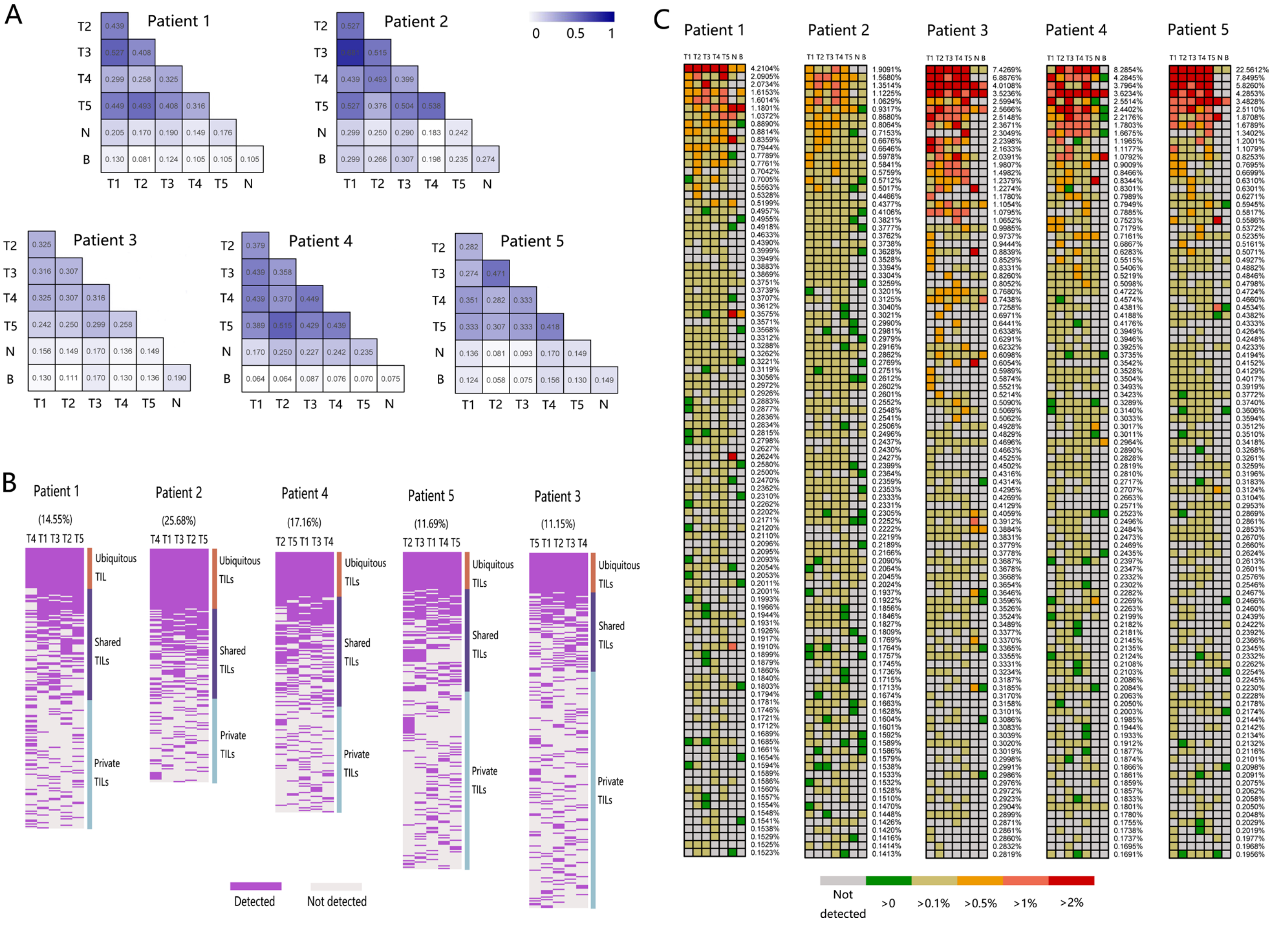

Supplementary Figure 2: The heterogeneity of the TCR repertoires of each HCC patient. (**A**) Comparison of TOP100 overlaps between different samples of each patient. For each patient we computed pairwise overlaps among all samples, the high overlap rate obtain a darker shade of blue in the heat map. (**B**) Spatial heterogeneity of TIL clonotype in HCC patients. Heat maps show the regional distribution of TOP100 from all tumor samples of each patient. T cell clones identified in its original regions showed purple, otherwise light grey. Column close to heat map show three categories of TIL populations: TILs present in all regions were defined as ubiquitous (salmon), in more than one but not all regions were considered as shared (modena) and in one region was regarded as private (light-blue). Both shared and private TILs were heterogeneous. Patient identification is showed on the top of figure. Then, the percentage of ubiquitous TIL clones of each patient is indicated. Next, lesion names in the form of regional identification. (**C**) Regional frequencies of the 100 most abundant T cell clones. The 100 most abundant TIL clones showed in heat maps identified as the highest regional frequencies throughout five biopsy regions of each tumor when including the top100 T cell clones. Frequencies that listed on the right of each heat map represent the corresponding T cell clones. The color of cell check indicates different frequency of T cell clone, the corresponding relationship between color and clone abundances are indicated by figure legend presents on the bottom of figure. Patient identifications and lesion names are showed on the top of figure.

P1T1

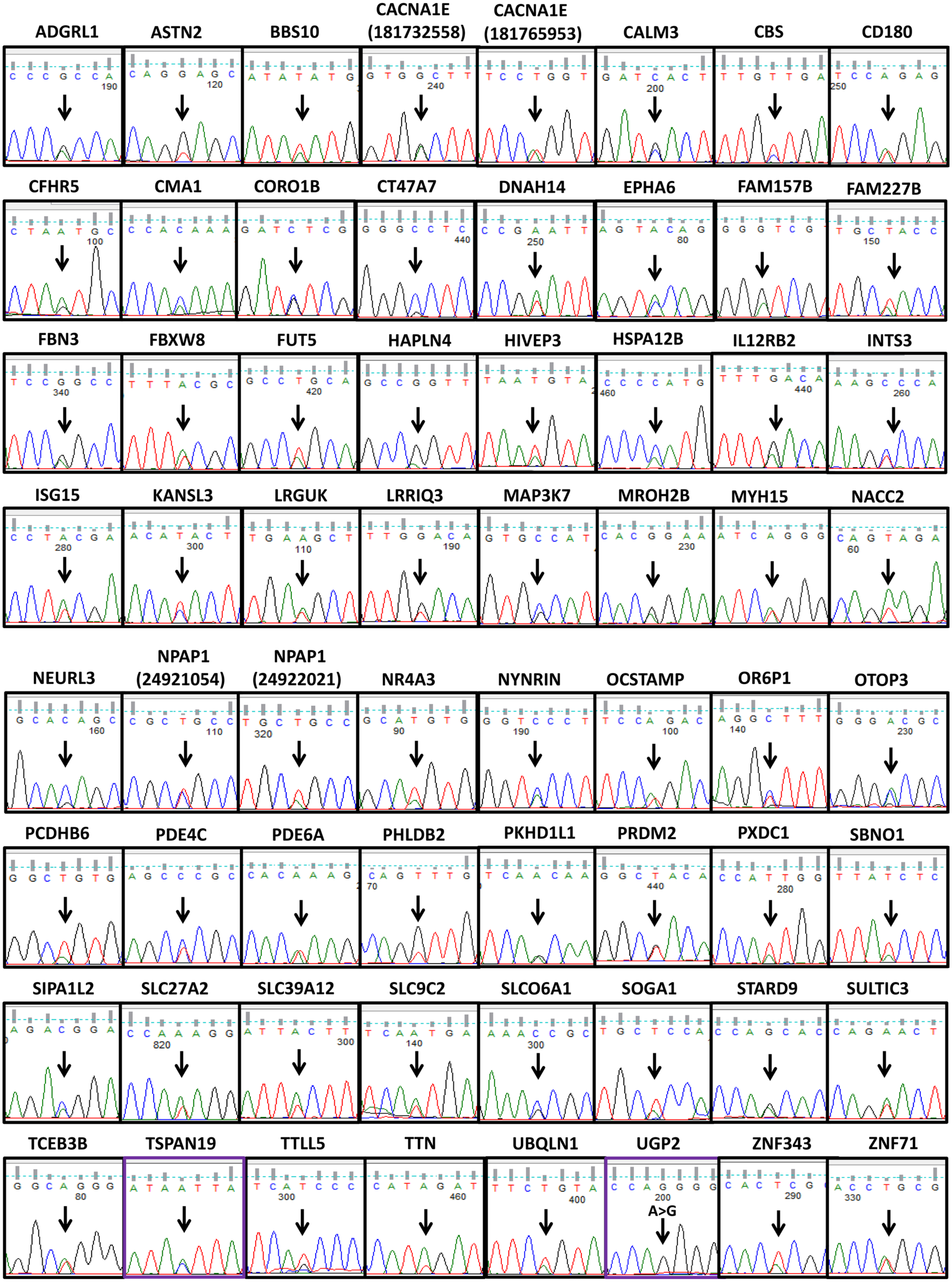
ADGRL1
ASTN2
BBS10
CACNA1E (181732558)
CACNA1E (181765953)
CALM3
CBS
CD180
CFHR5
CMA1
CORO1B
CT47A7
DNAH14
EPHA6
FAM157B
FAM227B
FBN3
FBXW8
FUT5
HAPLN4
HIVEP3
HSPA12B
IL12RB2
INTS3
ISG15
KANSL3
LRGUK
LRRIQ3
MAP3K7
MROH2B
MYH15
NACC2
NEURL3
NPAP1 (24921054)
NPAP1 (24922021)
NR4A3
NYNRIN
OCSTAMP
OR6P1
OTOP3
PCDHB6
PDE4C
PDE6A
PHLDB2
PKHD1L1
PRDM2
PXDC1
SBNO1
SIPA1L2
SLC27A2
SLC39A12
SLC9C2
SLCO6A1
SOGA1
STARD9
SULTIC3
TCEB3B
TSPAN19
TTLL5
TTN
UBQLN1
UGP2
A>G
ZNF343
ZNF71

P1T2

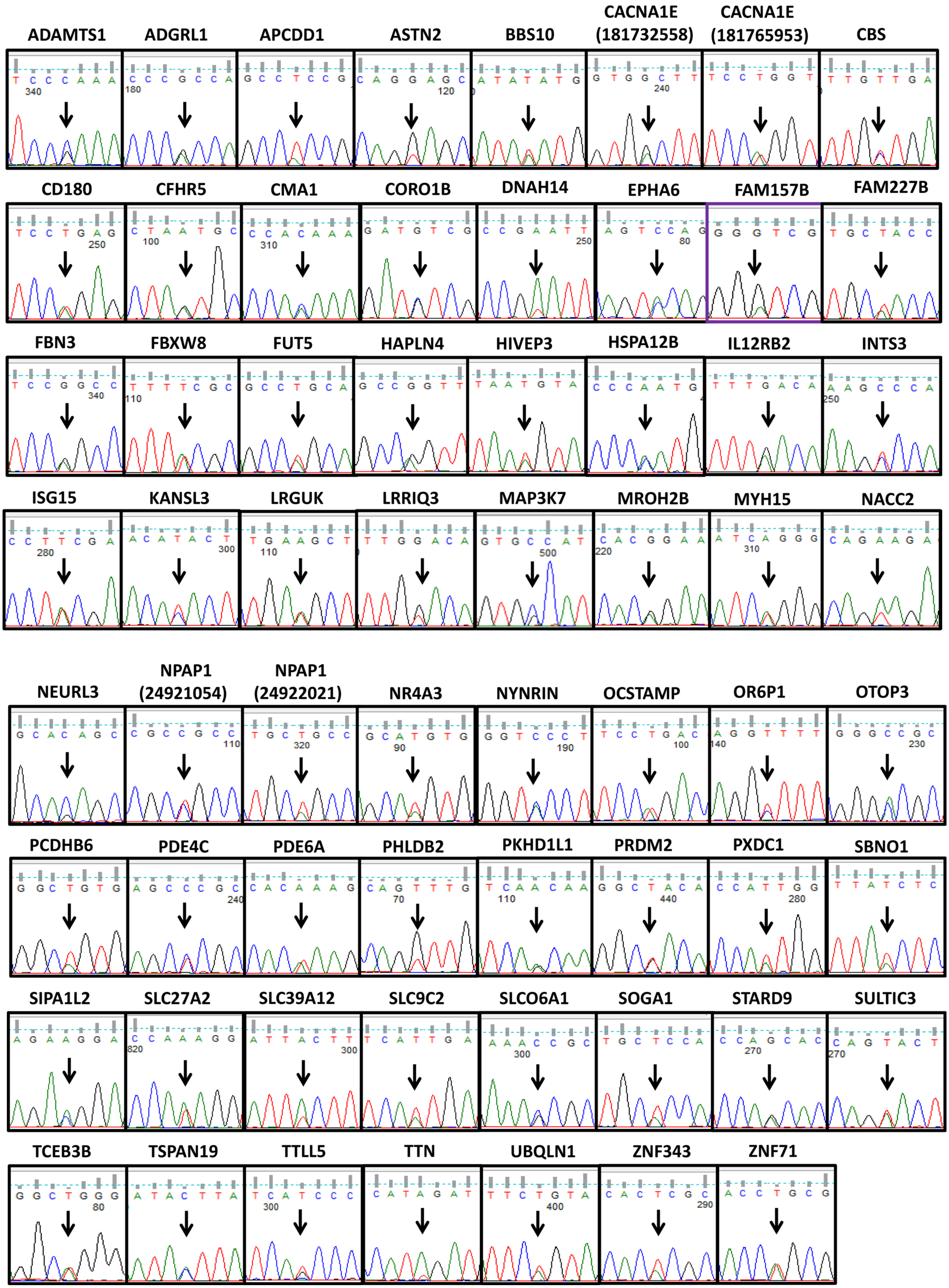
ADAMTS1
ADGRL1
APCDD1
ASTN2
BBS10
CACNA1E (181732558)
CACNA1E (181765953)
CBS
CD180
CFHR5
CMA1
CORO1B
DNAH14
EPHA6
FAM157B
FAM227B
FBN3
FBXW8
FUT5
HAPLN4
HIVEP3
HSPA12B
IL12RB2
INTS3
ISG15
KANSL3
LRGUK
LRRIQ3
MAP3K7
MROH2B
MYH15
NACC2
NEURL3
NPAP1 (24921054)
NPAP1 (24922021)
NR4A3
NYNRIN
OCSTAMP
OR6P1
OTOP3
PCDHB6
PDE4C
PDE6A
PHLDB2
PKHD1L1
PRDM2
PXDC1
SBNO1
SIPA1L2
SLC27A2
SLC39A12
SLC9C2
SLCO6A1
SOGA1
STARD9
SULTIC3
TCEB3B
TSPAN19
TTLL5
TTN
UBQLN1
ZNF343
ZNF71

P1T3

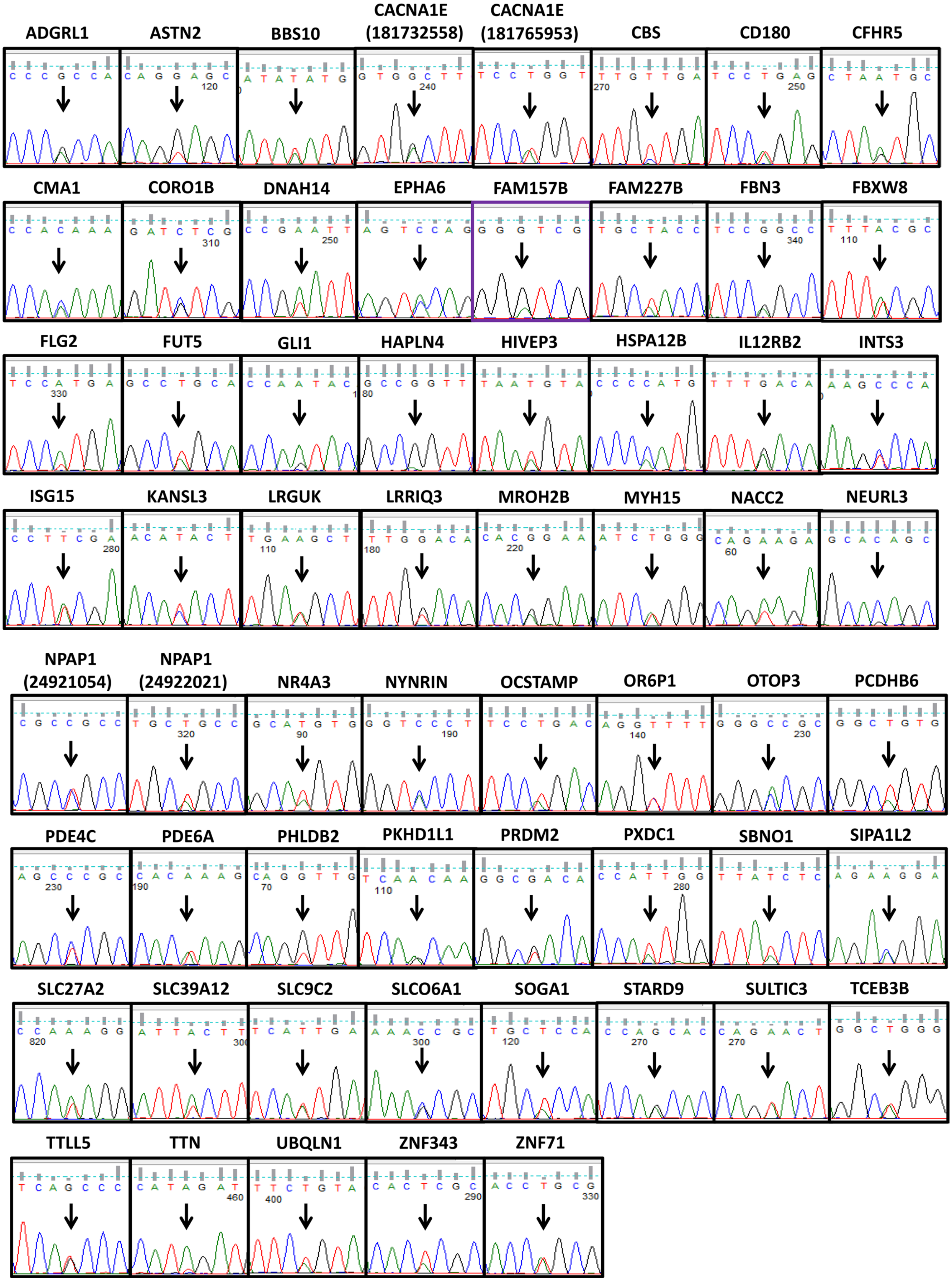
ADGRL1
ASTN2
BBS10
CACNA1E (181732558)
CACNA1E (181765953)
CBS
CD180
CFHR5
CMA1
CORO1B
DNAH14
EPHA6
FAM157B
FAM227B
FBN3
FBXW8
FLG2
FUT5
GLI1
HAPLN4
HIVEP3
HSPA12B
IL12RB2
INTS3
ISG15
KANSL3
LRGUK
LRRIQ3
MROH2B
MYH15
NACC2
NEURL3
NPAP1 (24921054)
NPAP1 (24922021)
NR4A3
NYNRIN
OCSTAMP
OR6P1
OTOP3
PCDHB6
PDE4C
PDE6A
PHLDB2
PKHD1L1
PRDM2
PXDC1
SBNO1
SIPA1L2
SLC27A2
SLC39A12
SLC9C2
SLCO6A1
SOGA1
STARD9
SULTIC3
TCEB3B
TTLL5
TTN
UBQLN1
ZNF343
ZNF71

P1T4

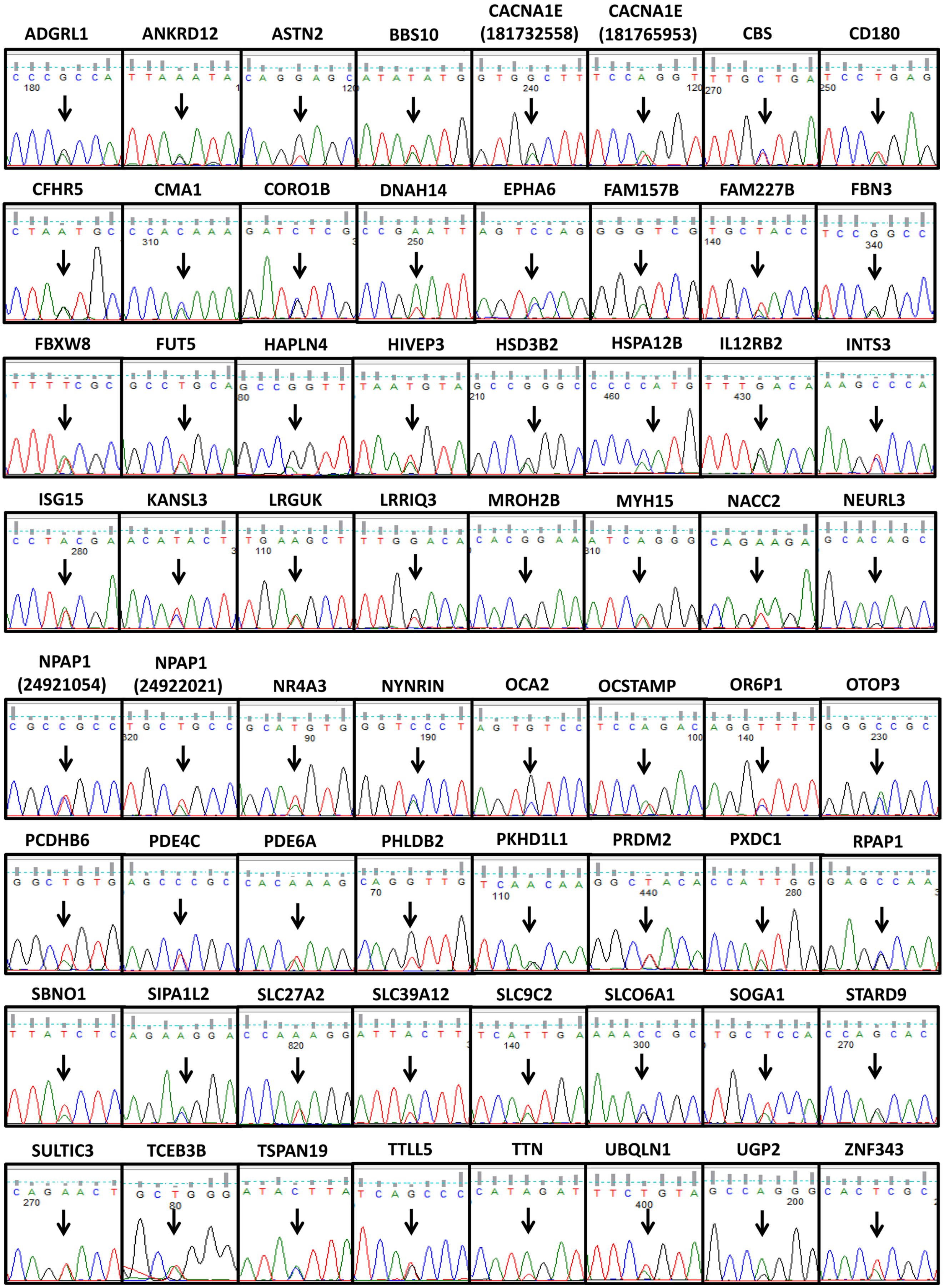
ADGRL1
ANKRD12
ASTN2
BBS10
CACNA1E (181732558)
CACNA1E (181765953)
CBS
CD180
CFHR5
CMA1
CORO1B
DNAH14
EPHA6
FAM157B
FAM227B
FBN3
FBXW8
FUT5
HAPLN4
HIVEP3
HSD3B2
HSPA12B
IL12RB2
INTS3
ISG15
KANSL3
LRGUK
LRRIQ3
MROH2B
MYH15
NACC2
NEURL3
NPAP1 (24921054)
NPAP1 (24922021)
NR4A3
NYNRIN
OCA2
OCSTAMP
OR6P1
OTOP3
PCDHB6
PDE4C
PDE6A
PHLDB2
PKHD1L1
PRDM2
PXDC1
RPAP1
SBNO1
SIPA1L2
SLC27A2
SLC39A12
SLC9C2
SLCO6A1
SOGA1
STARD9
SULTIC3
TCEB3B
TSPAN19
TTLL5
TTN
UBQLN1
UGP2
ZNF343

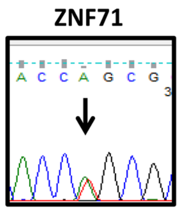
ZNF71

P1T5

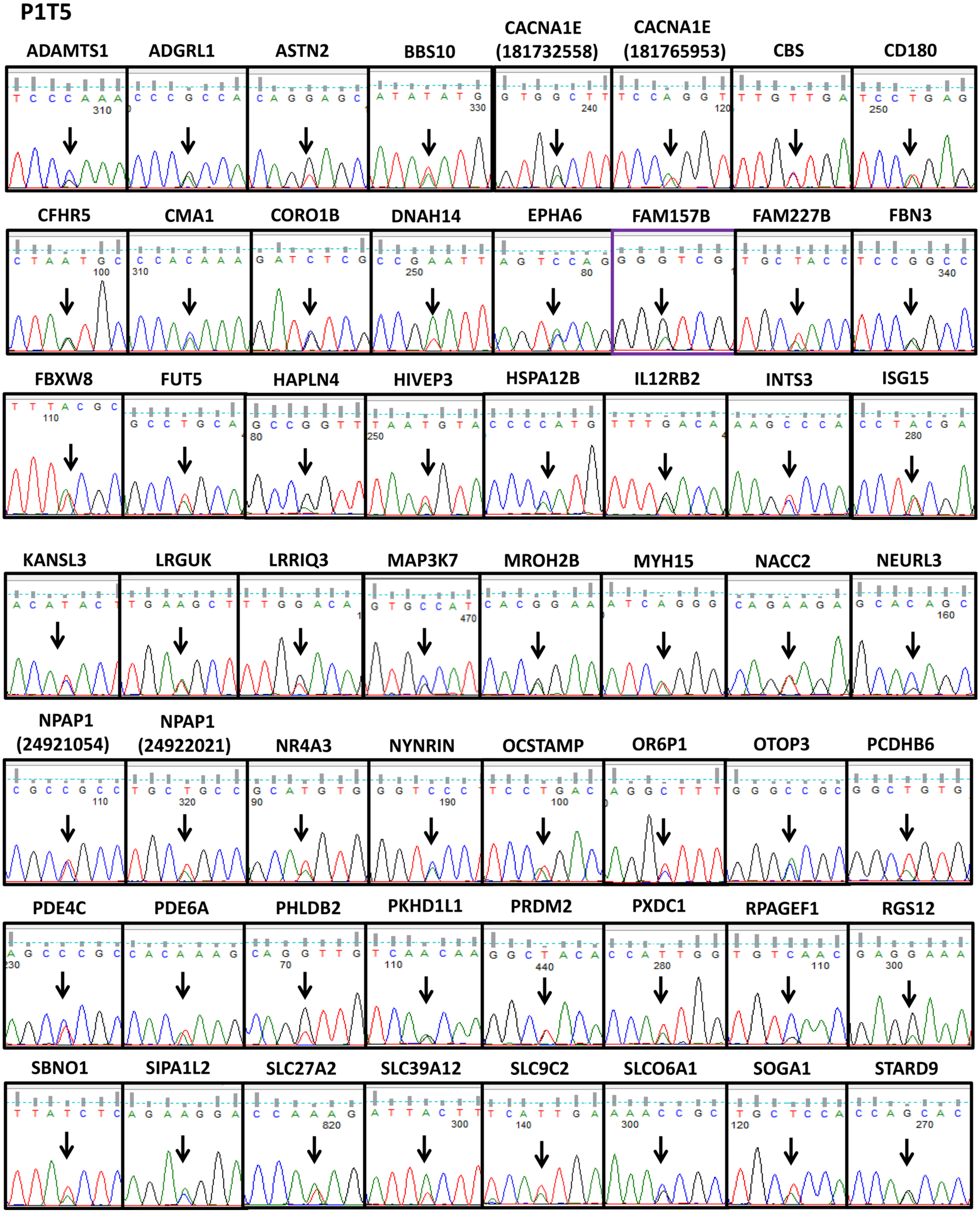
ADAMTS1
ADGRL1
ASTN2
BBS10
CACNA1E (181732558)
CACNA1E (181765953)
CBS
CD180
CFHR5
CMA1
CORO1B
DNAH14
EPHA6
FAM157B
FAM227B
FBN3
FBXW8
FUT5
HAPLN4
HIVEP3
HSPA12B
IL12RB2
INTS3
ISG15
KANSL3
LRGUK
LRRIQ3
MAP3K7
MROH2B
MYH15
NACC2
NEURL3
NPAP1 (24921054)
NPAP1 (24922021)
NR4A3
NYNRIN
OCSTAMP
OR6P1
OTOP3
PCDHB6
PDE4C
PDE6A
PHLDB2
PKHD1L1
PRDM2
PXDC1
RPAGEF1
RGS12
SBNO1
SIPA1L2
SLC27A2
SLC39A12
SLC9C2
SLCO6A1
SOGA1
STARD9

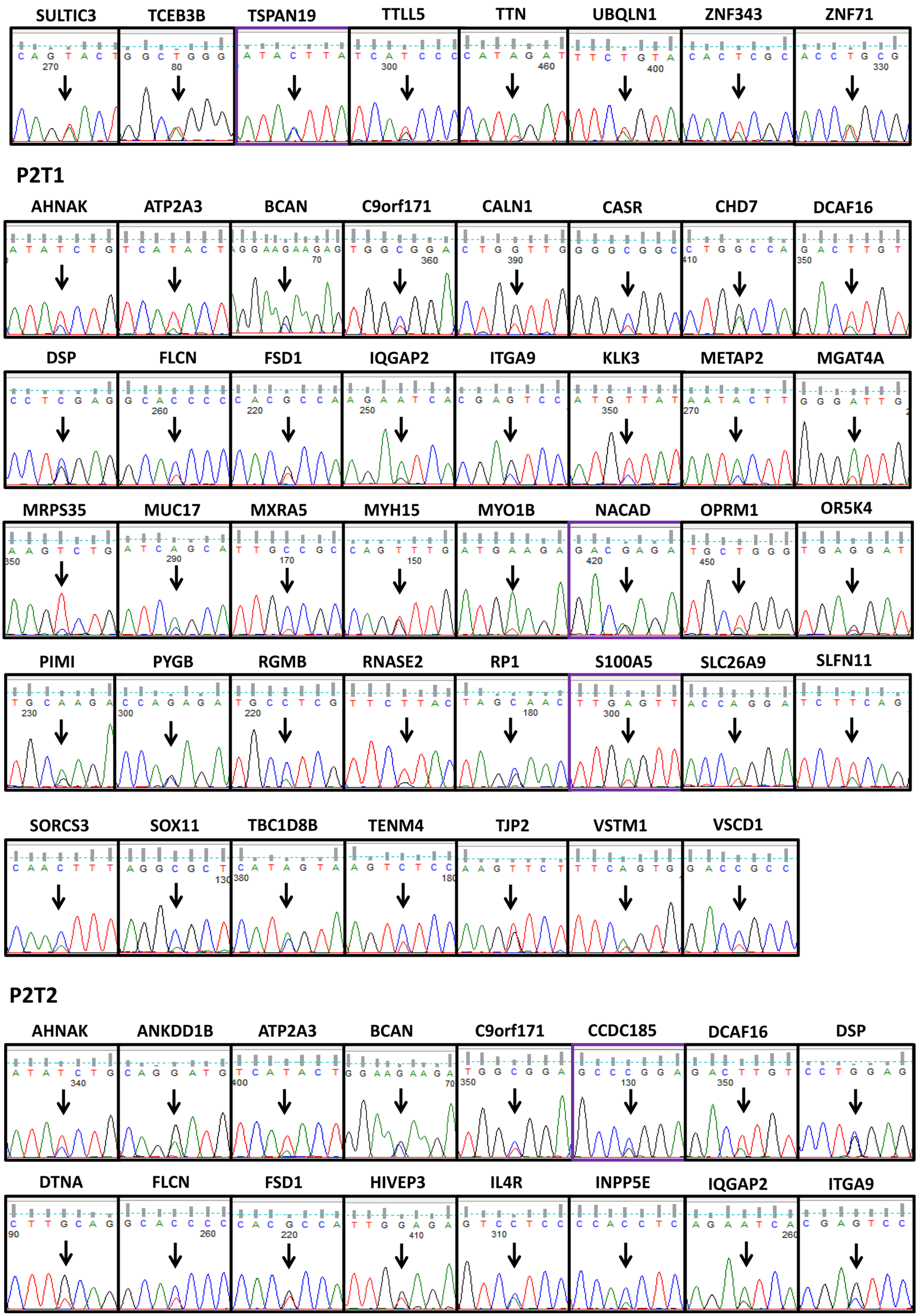
SULTIC3
TCEB3B
TSPAN19
TTLL5
TTN
UBQLN1
ZNF343
ZNF71
P2T1
AHNAK
ATP2A3
BCAN
C9orf171
CALN1
CASR
CHD7
DCAF16
DSP
FLCN
FSD1
IQGAP2
ITGA9
KLK3
METAP2
MGAT4A
MRPS35
MUC17
MXRA5
MYH15
MYO1B
NACAD
OPRM1
OR5K4
PIMI
PYGB
RGMB
RNASE2
RP1
S100A5
SLC26A9
SLFN11
SORCS3
SOX11
TBC1D8B
TENM4
TJP2
VSTM1
VSCD1
P2T2
AHNAK
ANKDD1B
ATP2A3
BCAN
C9orf171
CCDC185
DCAF16
DSP
DTNA
FLCN
FSD1
HIVEP3
IL4R
INPP5E
IQGAP2
ITGA9

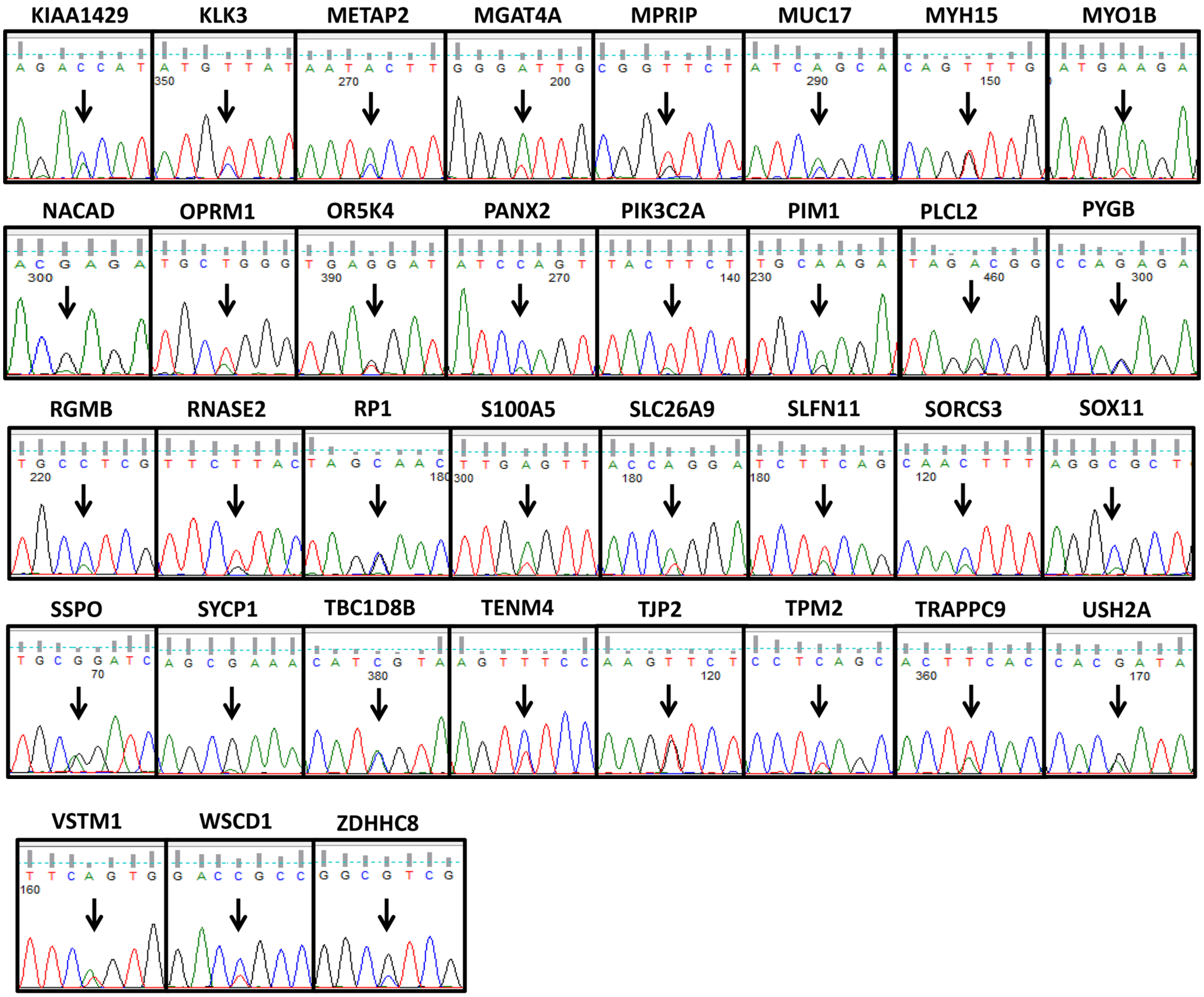
KIAA1429
KLK3
METAP2
MGAT4A
MPRIP
MUC17
MYH15
MYO1B
NACAD
OPRM1
OR5K4
PANX2
PIK3C2A
PIM1
PLCL2
PYGB
RGMB
RNASE2
RP1
S100A5
SLC26A9
SLFN11
SORCS3
SOX11
SSPO
SYCP1
TBC1D8B
TENM4
TJP2
TPM2
TRAPPC9
USH2A
VSTM1
WSCD1
ZDHHC8

P2T3

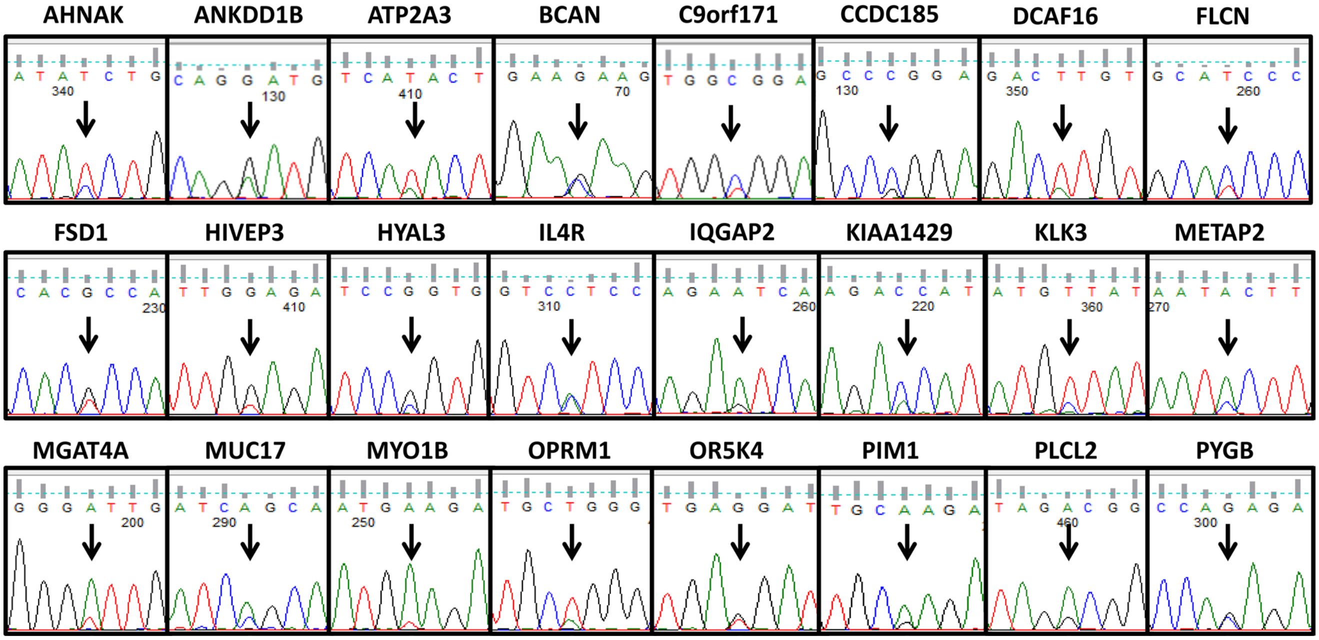
AHNAK
ANKDD1B
ATP2A3
BCAN
C9orf171
CCDC185
DCAF16
FLCN
FSD1
HIVEP3
HYAL3
IL4R
IQGAP2
KIAA1429
KLK3
METAP2
MGAT4A
MUC17
MYO1B
OPRM1
OR5K4
PIM1
PLCL2
PYGB

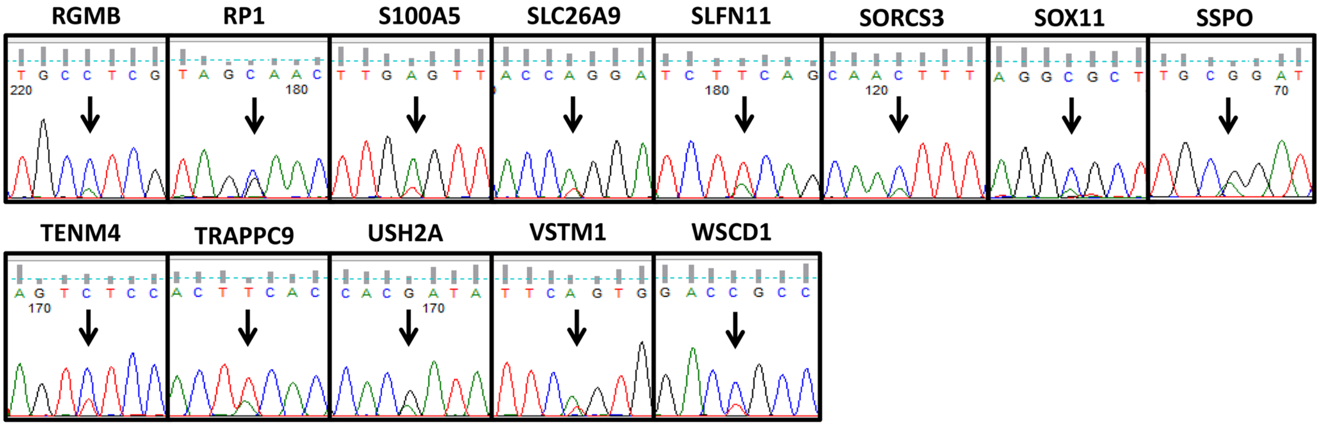
RGMB
RP1
S100A5
SLC26A9
SLFN11
SORCS3
SOX11
SSPO
TENM4
TRAPPC9
USH2A
VSTM1
WSCD1

P2T4

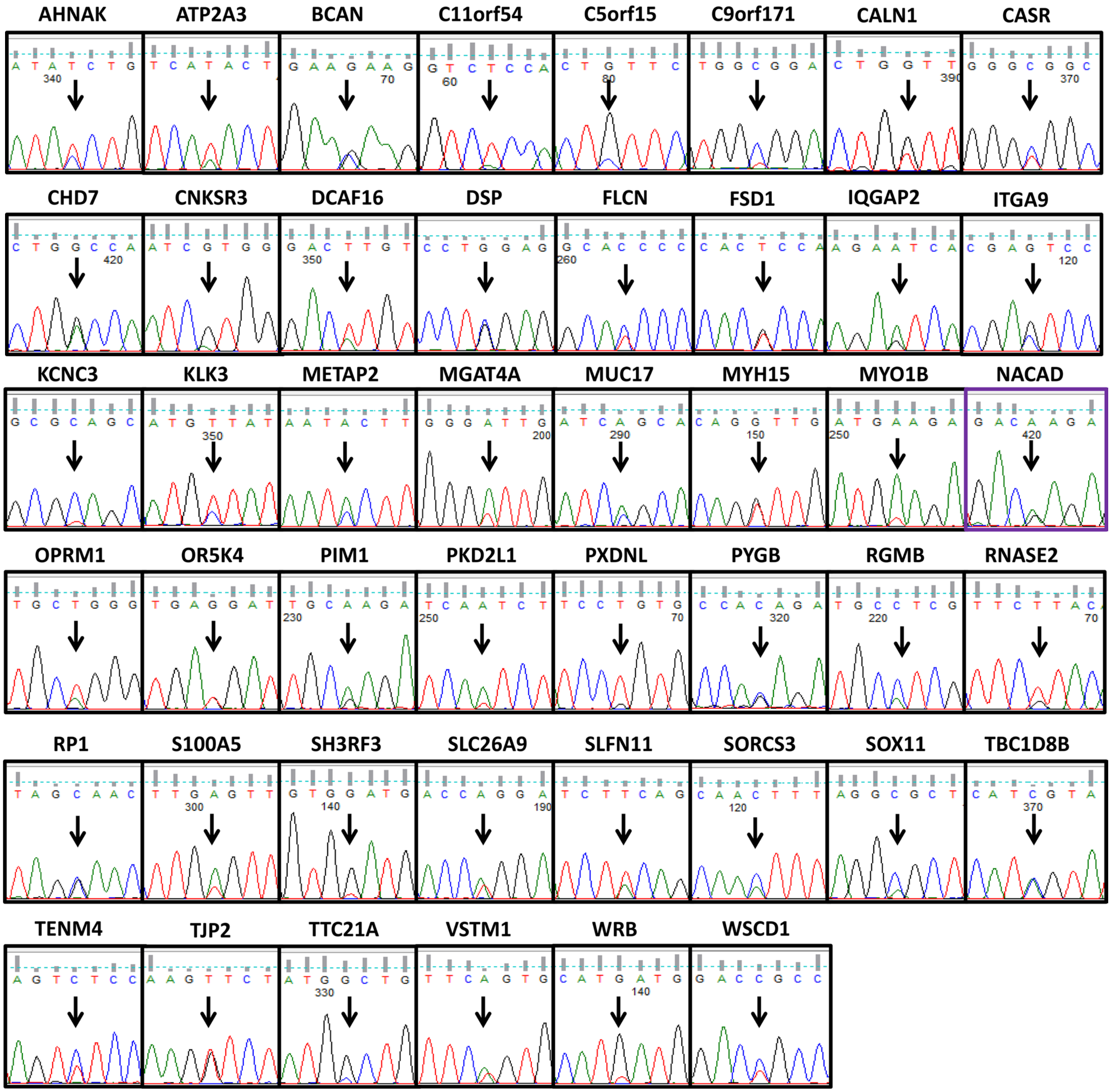
AHNAK
ATP2A3
BCAN
C11orf54
C5orf15
C9orf171
CALN1
CASR
CHD7
CNKSR3
DCAF16
DSP
FLCN
FSD1
IQGAP2
ITGA9
KCNC3
KLK3
METAP2
MGAT4A
MUC17
MYH15
MYO1B
NACAD
OPRM1
OR5K4
PIM1
PKD2L1
PXDNL
PYGB
RGMB
RNASE2
RP1
S100A5
SH3RF3
SLC26A9
SLFN11
SORCS3
SOX11
TBC1D8B
TENM4
TJP2
TTC21A
VSTM1
WRB
WSCD1

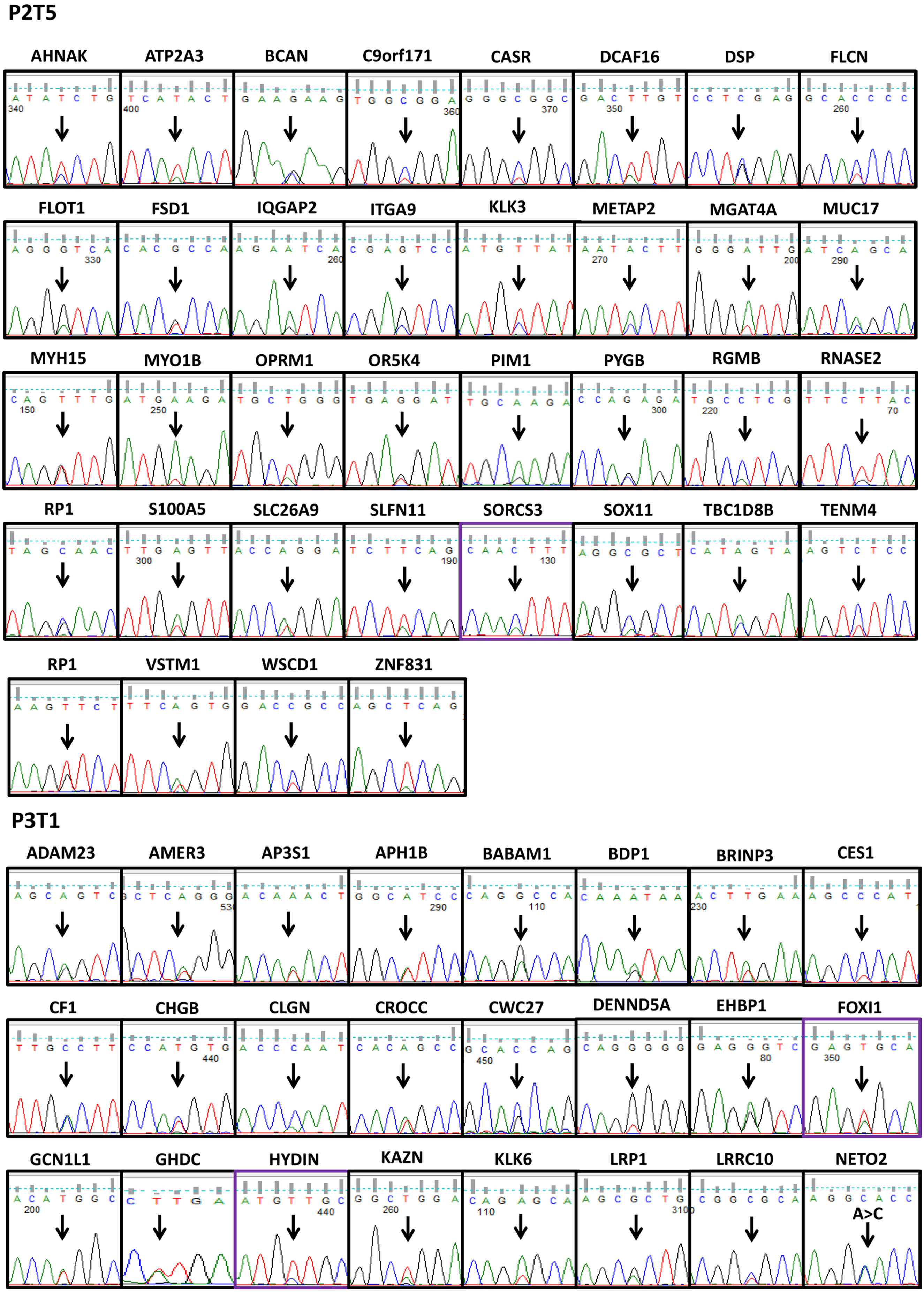
P2T5
AHNAK
ATP2A3
BCAN
C9orf171
CASR
DCAF16
DSP
FLCN
FLOT1
FSD1
IQGAP2
ITGA9
KLK3
METAP2
MGAT4A
MUC17
MYH15
MYO1B
OPRM1
OR5K4
PIM1
PYGB
RGMB
RNASE2
RP1
S100A5
SLC26A9
SLFN11
SORCS3
SOX11
TBC1D8B
TENM4
RP1
VSTM1
WSCD1
ZNF831
P3T1
ADAM23
AMER3
AP3S1
APH1B
BABAM1
BDP1
BRINP3
CES1
CF1
CHGB
CLGN
CROCC
CWC27
DENND5A
EHBP1
FOXI1
GCN1L1
GHDC
HYDIN
KAZN
KLK6
LRP1
LRRC10
NETO2
A>C

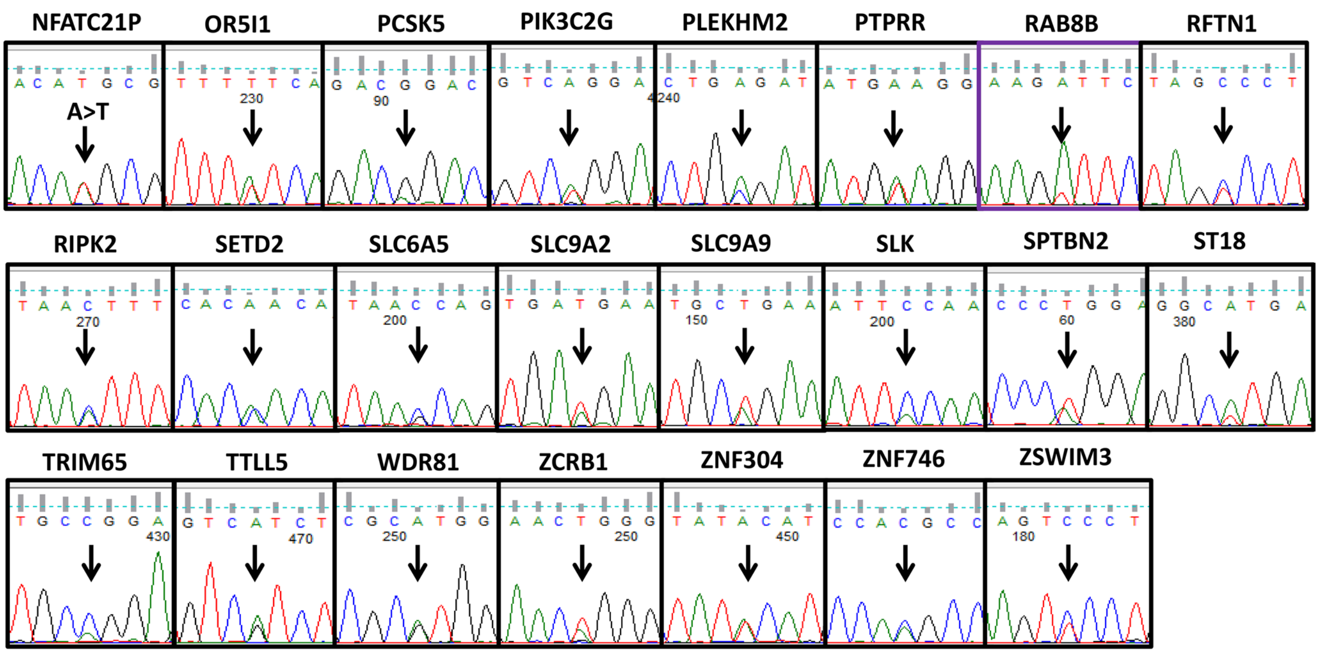
NFATC21P
OR5I1
PCSK5
PIK3C2G
PLEKHM2
PTPRR
RAB8B
RFTN1
A>T
RIPK2
SETD2
SLC6A5
SLC9A2
SLC9A9
SLK
SPTBN2
ST18
TRIM65
TTLL5
WDR81
ZCRB1
ZNF304
ZNF746
ZSWIM3

P3T2

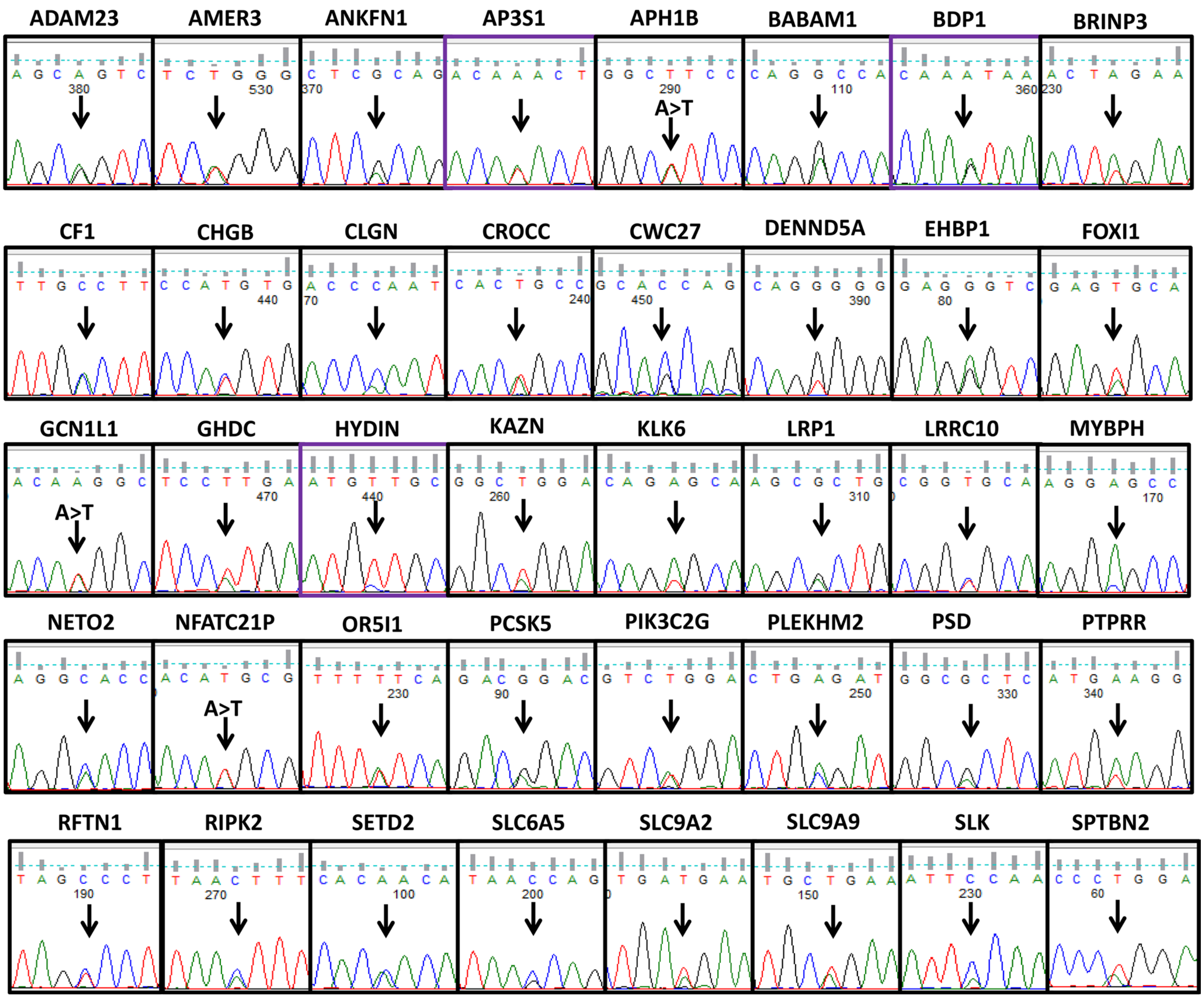
ADAM23
AMER3
ANKFN1
AP3S1
APH1B
BABAM1
BDP1
BRINP3
A>T
CF1
CHGB
CLGN
CROCC
CWC27
DENND5A
EHBP1
FOXI1
GCN1L1
GHDC
HYDIN
KAZN
KLK6
LRP1
LRRC10
MYBPH
A>T
NETO2
NFATC21P
OR5I1
PCSK5
PIK3C2G
PLEKHM2
PSD
PTPRR
A>T
RFTN1
RIPK2
SETD2
SLC6A5
SLC9A2
SLC9A9
SLK
SPTBN2

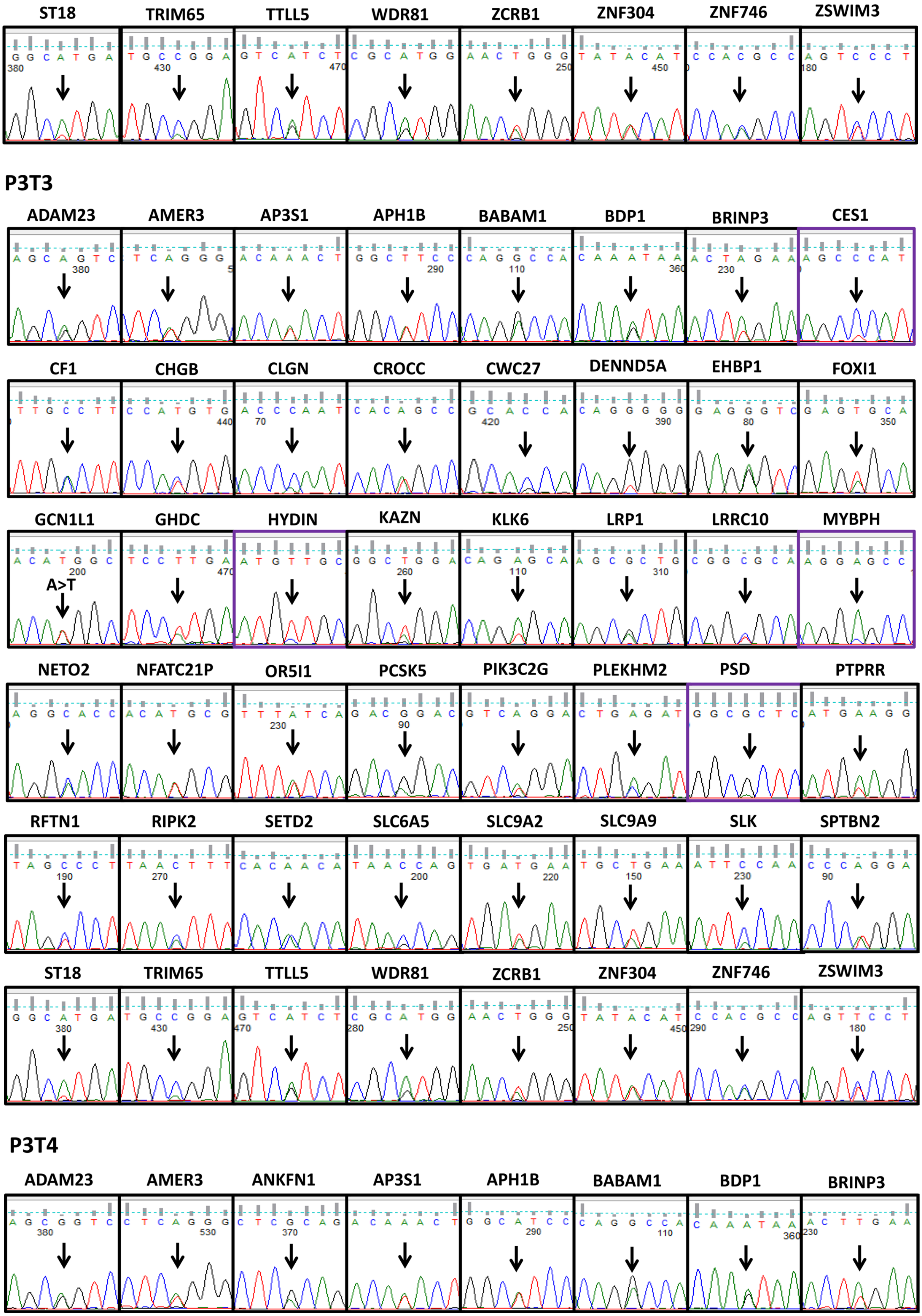

ST18
TRIM65
TTLL5
WDR81
ZCRB1
ZNF304
ZNF746
ZSWIM3
P3T3
ADAM23
AMER3
AP3S1
APH1B
BABAM1
BDP1
BRINP3
CES1
CF1
CHGB
CLGN
CROCC
CWC27
DENND5A
EHBP1
FOXI1
GCN1L1
A>T
GHDC
HYDIN
KAZN
KLK6
LRP1
LRRC10
MYBPH
NETO2
NFATC21P
OR5I1
PCSK5
PIK3C2G
PLEKHM2
PSD
PTPRR
RFTN1
RIPK2
SETD2
SLC6A5
SLC9A2
SLC9A9
SLK
SPTBN2
ST18
TRIM65
TTLL5
WDR81
ZCRB1
ZNF304
ZNF746
ZSWIM3
P3T4
ADAM23
AMER3
ANKFN1
AP3S1
APH1B
BABAM1
BDP1
BRINP3

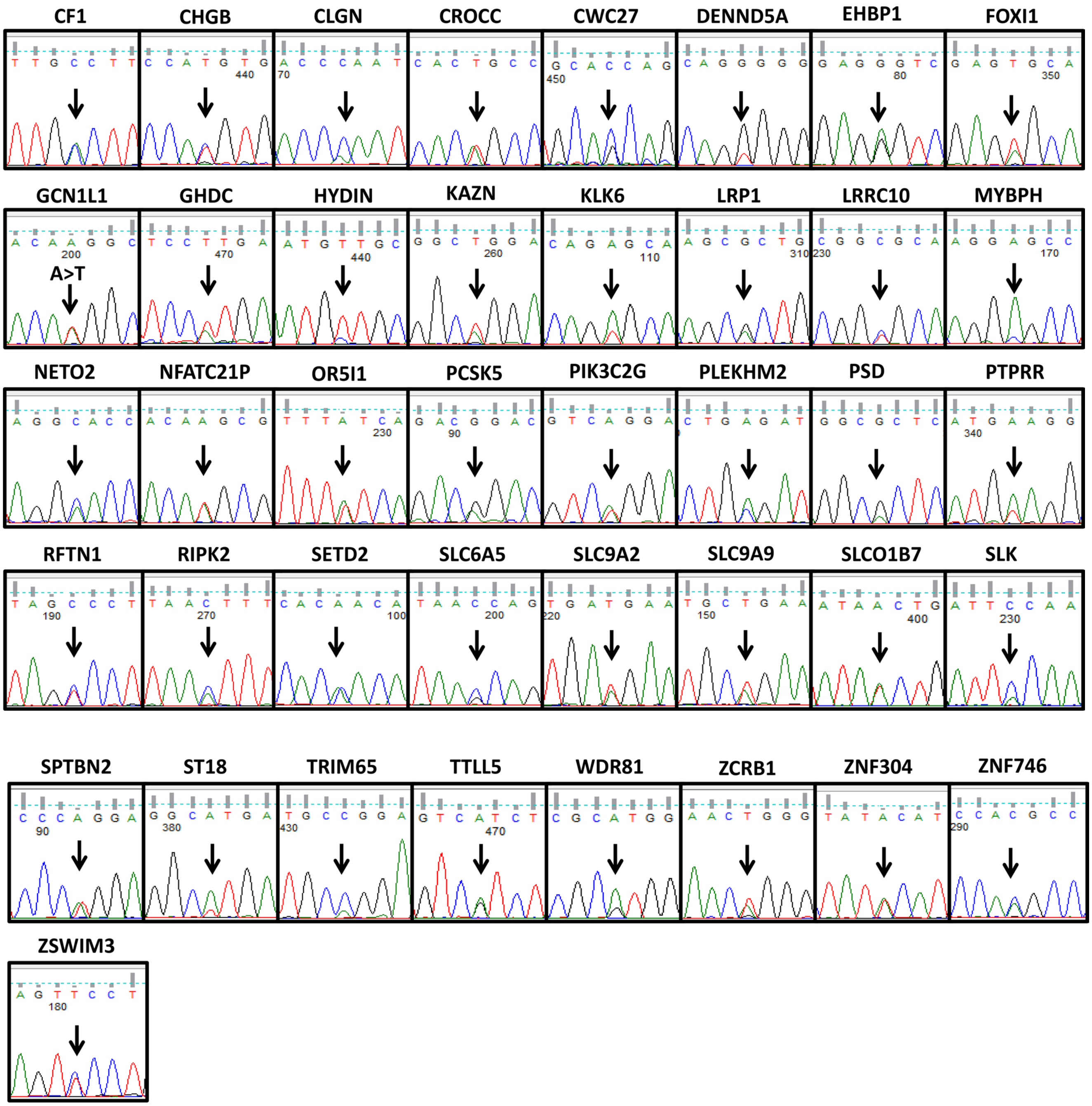
CF1
CHGB
CLGN
CROCC
CWC27
DENND5A
EHBP1
FOXI1
GCN1L1
A>T
GHDC
HYDIN
KAZN
KLK6
LRP1
LRRC10
MYBPH
NETO2
NFATC21P
OR5I1
PCSK5
PIK3C2G
PLEKHM2
PSD
PTPRR
RFTN1
RIPK2
SETD2
SLC6A5
SLC9A2
SLC9A9
SLCO1B7
SLK
SPTBN2
ST18
TRIM65
TTLL5
WDR81
ZCRB1
ZNF304
ZNF746
ZSWIM3

P3T5

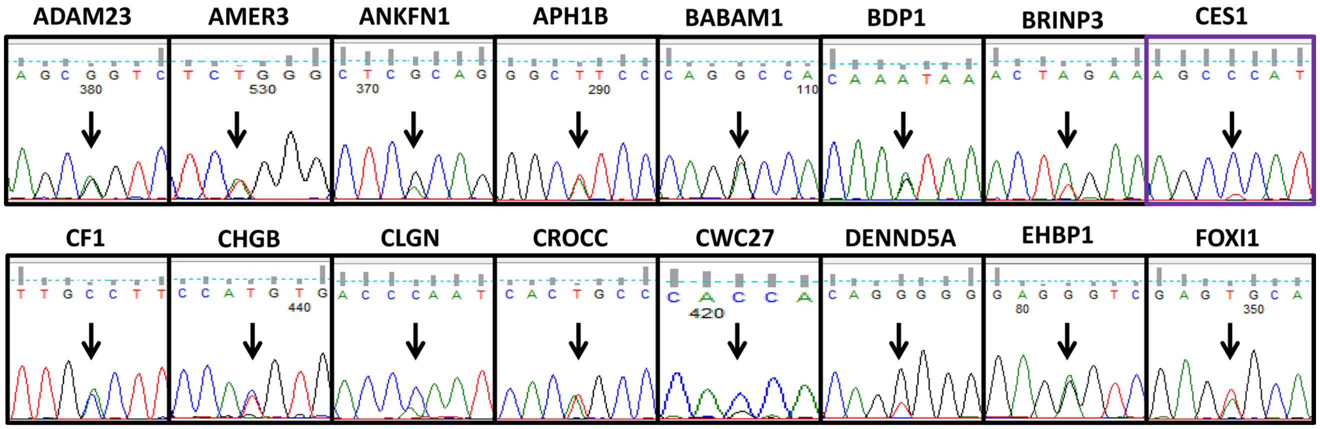
ADAM23
AMER3
ANKFN1
APH1B
BABAM1
BDP1
BRINP3
CES1
CF1
CHGB
CLGN
CROCC
CWC27
DENND5A
EHBP1
FOXI1

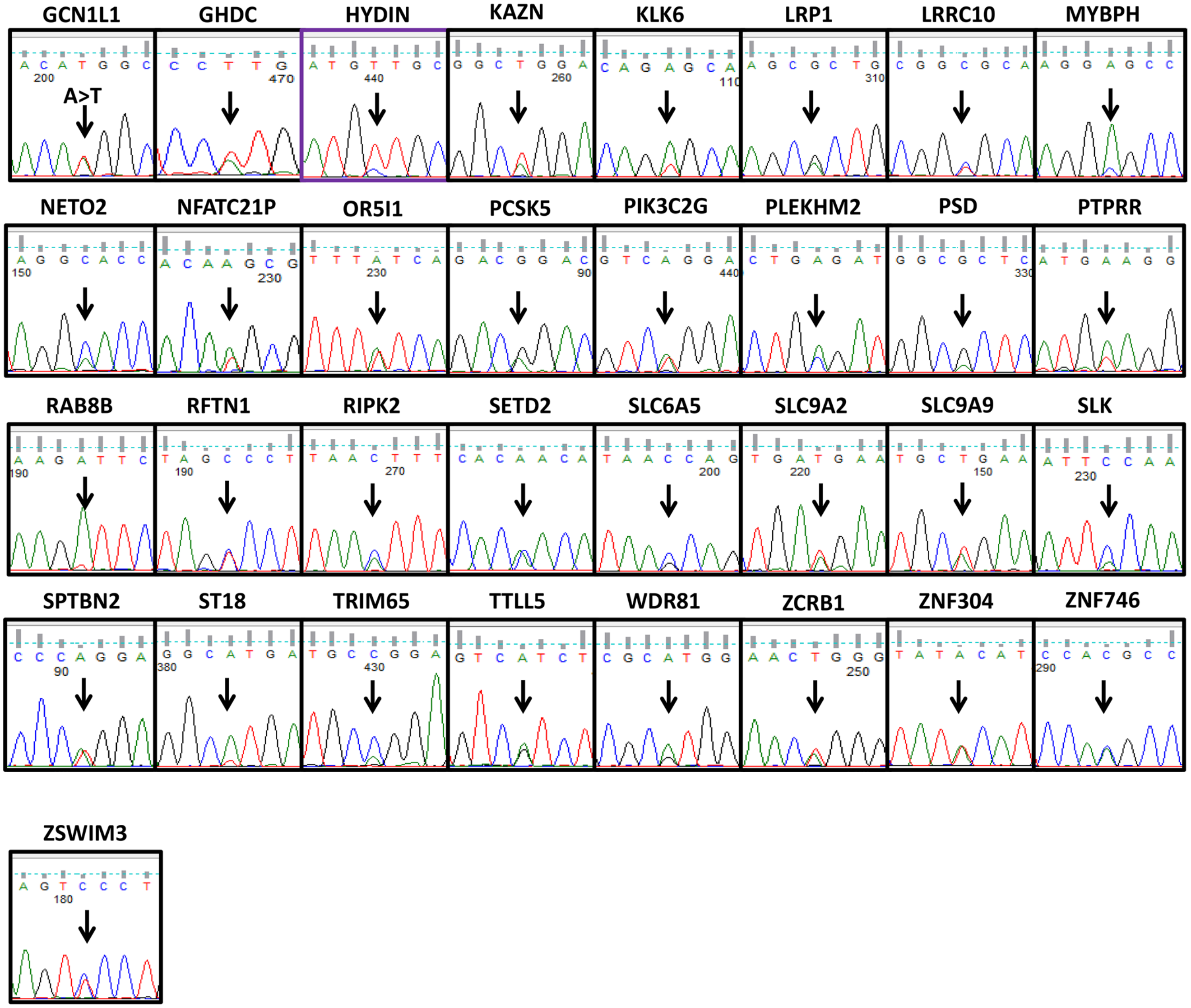
GCN1L1
A>T
GHDC
HYDIN
KAZN
KLK6
LRP1
LRRC10
MYBPH
NETO2
NFATC21P
OR5I1
PCSK5
PIK3C2G
PLEKHM2
PSD
PTPRR
RAB8B
RFTN1
RIPK2
SETD2
SLC6A5
SLC9A2
SLC9A9
SLK
SPTBN2
ST18
TRIM65
TTLL5
WDR81
ZCRB1
ZNF304
ZNF746
ZSWIM3

P4T1

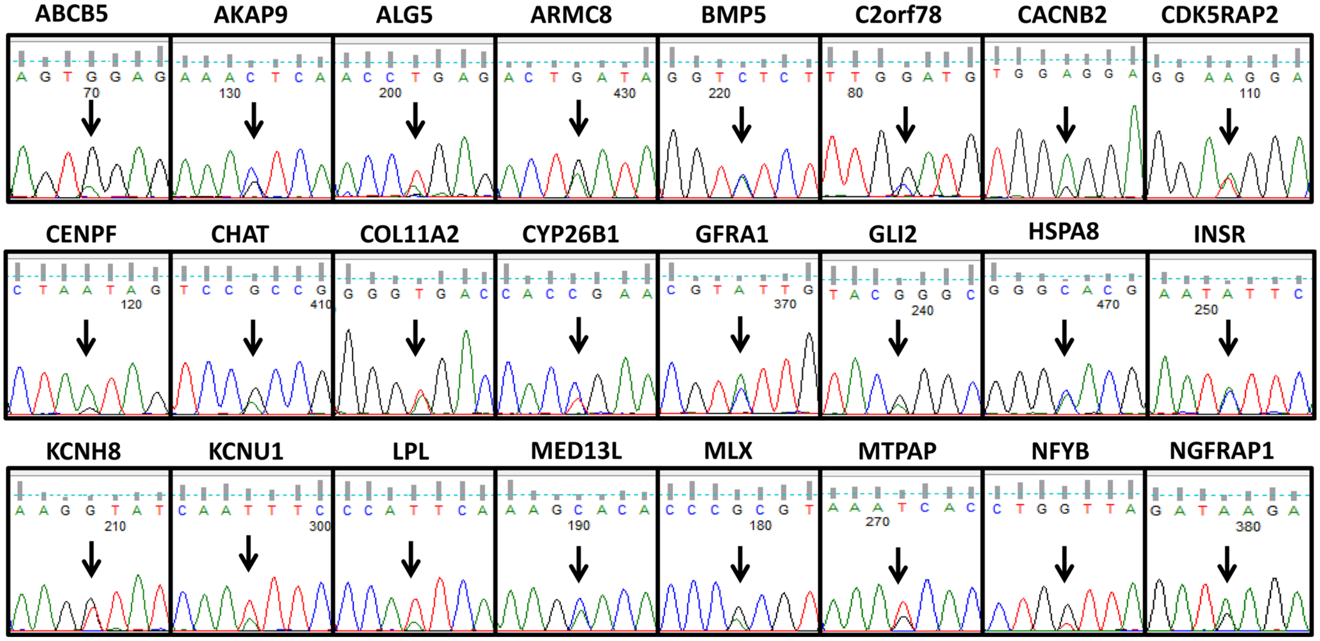
ABCB5
AKAP9
ALG5
ARMC8
BMP5
C2orf78
CACNB2
CDK5RAP2
CENPF
CHAT
COL11A2
CYP26B1
GFRA1
GLI2
HSPA8
INSR
KCNH8
KCNU1
LPL
MED13L
MLX
MTPAP
NFYB
NGFRAP1

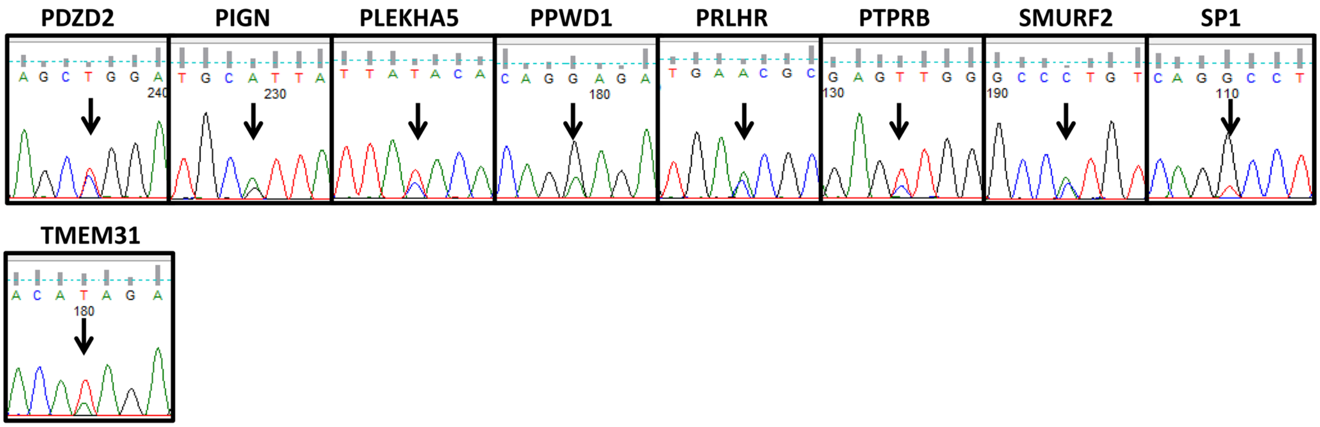
PDZD2
PIGN
PLEKHA5
PPWD1
PRLHR
PTPRB
SMURF2
SP1
TMEM31

P4T2

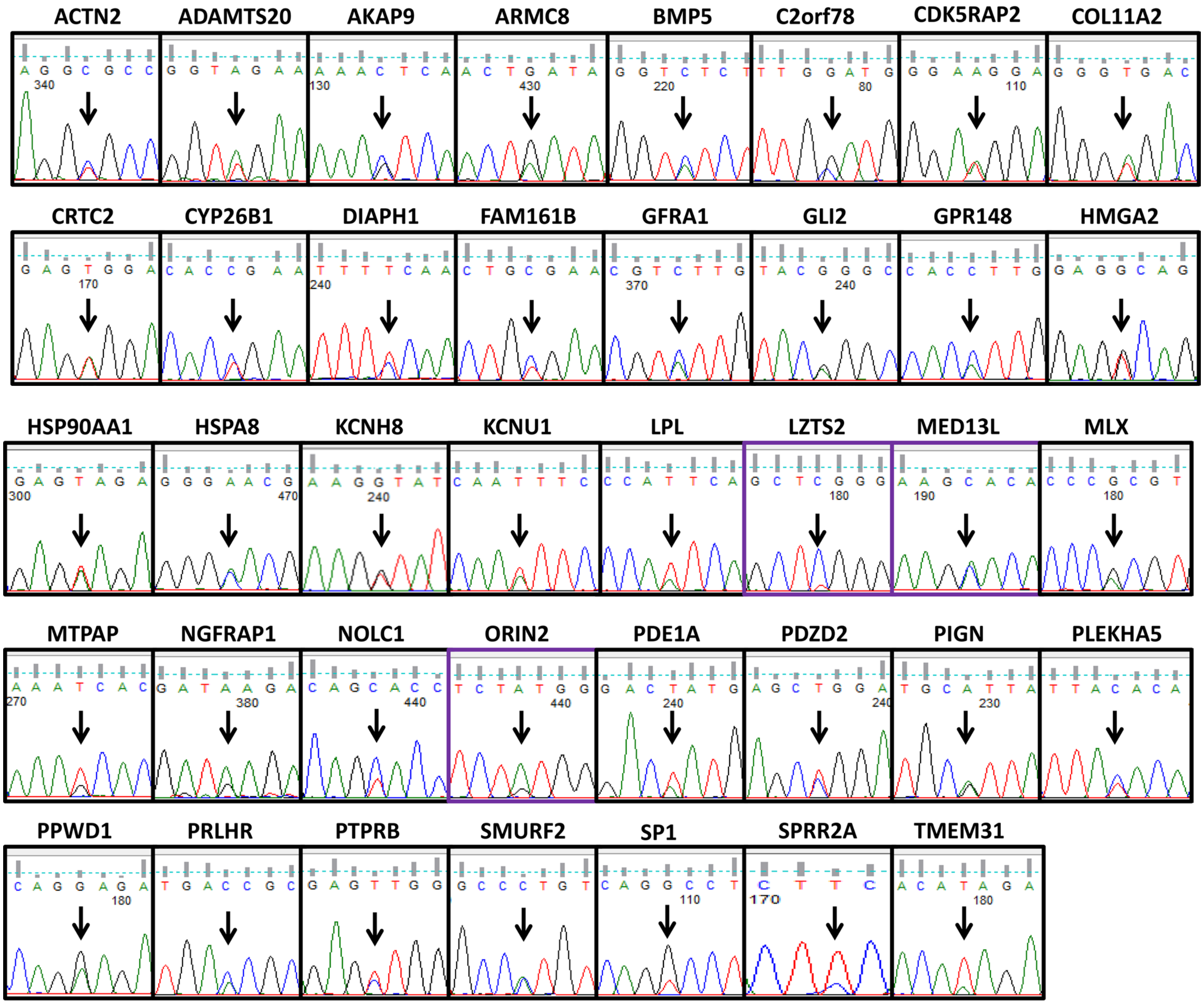
ACTN2
ADAMTS20
AKAP9
ARMC8
BMP5
C2orf78
CDK5RAP2
COL11A2
CRTC2
CYP26B1
DIAPH1
FAM161B
GFRA1
GLI2
GPR148
HMGA2
HSP90AA1
HSPA8
KCNH8
KCNU1
LPL
LZTS2
MED13L
MLX
MTPAP
NGFRAP1
NOLC1
ORIN2
PDE1A
PDZD2
PIGN
PLEKHA5
PPWD1
PRLHR
PTPRB
SMURF2
SP1
SPRR2A
TMEM31

P4T3

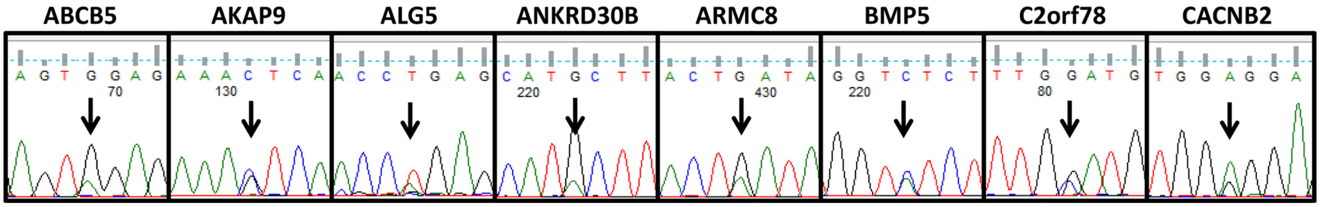
ABCB5
AKAP9
ALG5
ANKRD30B
ARMC8
BMP5
C2orf78
CACNB2

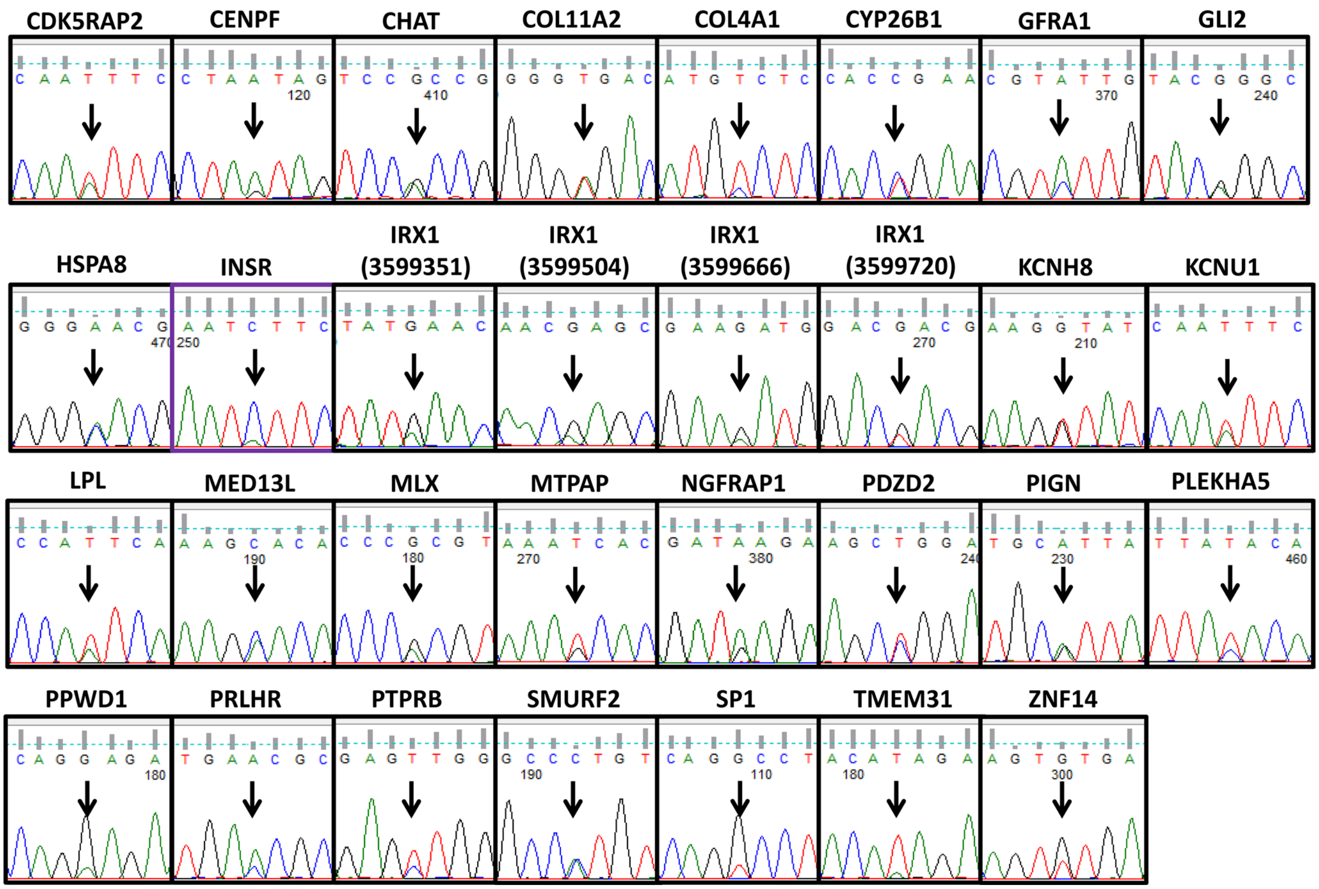
CDK5RAP2
CENPF
CHAT
COL11A2
COL4A1
CYP26B1
GFRA1
GLI2
HSPA8
INSR
IRX1 (3599351)
IRX1 (3599504)
IRX1 (3599666)
IRX1 (3599720)
KCNH8
KCNU1
LPL
MED13L
MLX
MTPAP
NGFRAP1
PDZD2
PIGN
PLEKHA5
PPWD1
PRLHR
PTPRB
SMURF2
SP1
TMEM31
ZNF14

P4T4

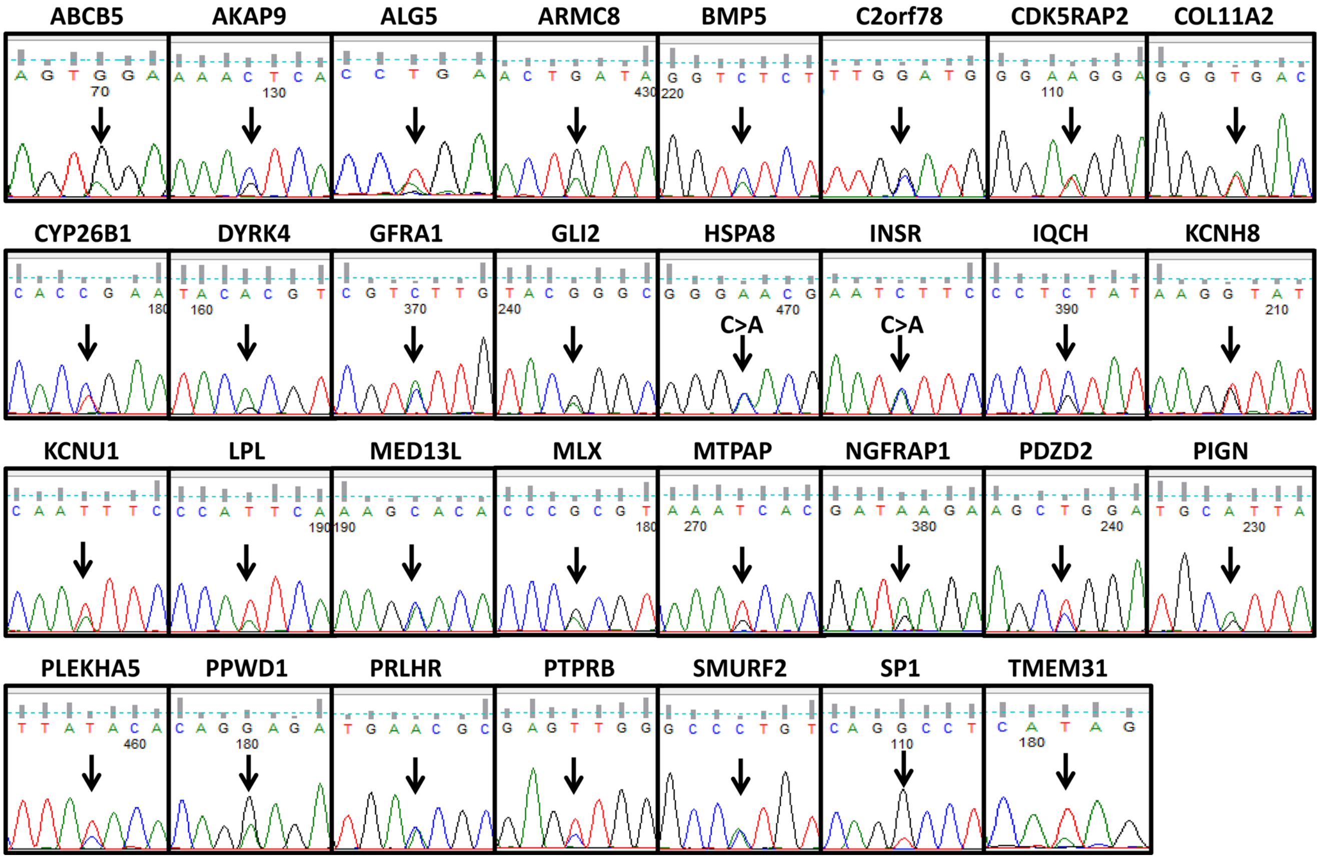
ABCB5
AKAP9
ALG5
ARMC8
BMP5
C2orf78
CDK5RAP2
COL11A2
CYP26B1
DYRK4
GFRA1
GLI2
HSPA8
C>A
INSR
C>A
IQCH
KCNH8
KCNU1
LPL
MED13L
MLX
MTPAP
NGFRAP1
PDZD2
PIGN
PLEKHA5
PPWD1
PRLHR
PTPRB
SMURF2
SP1
TMEM31

P4T5

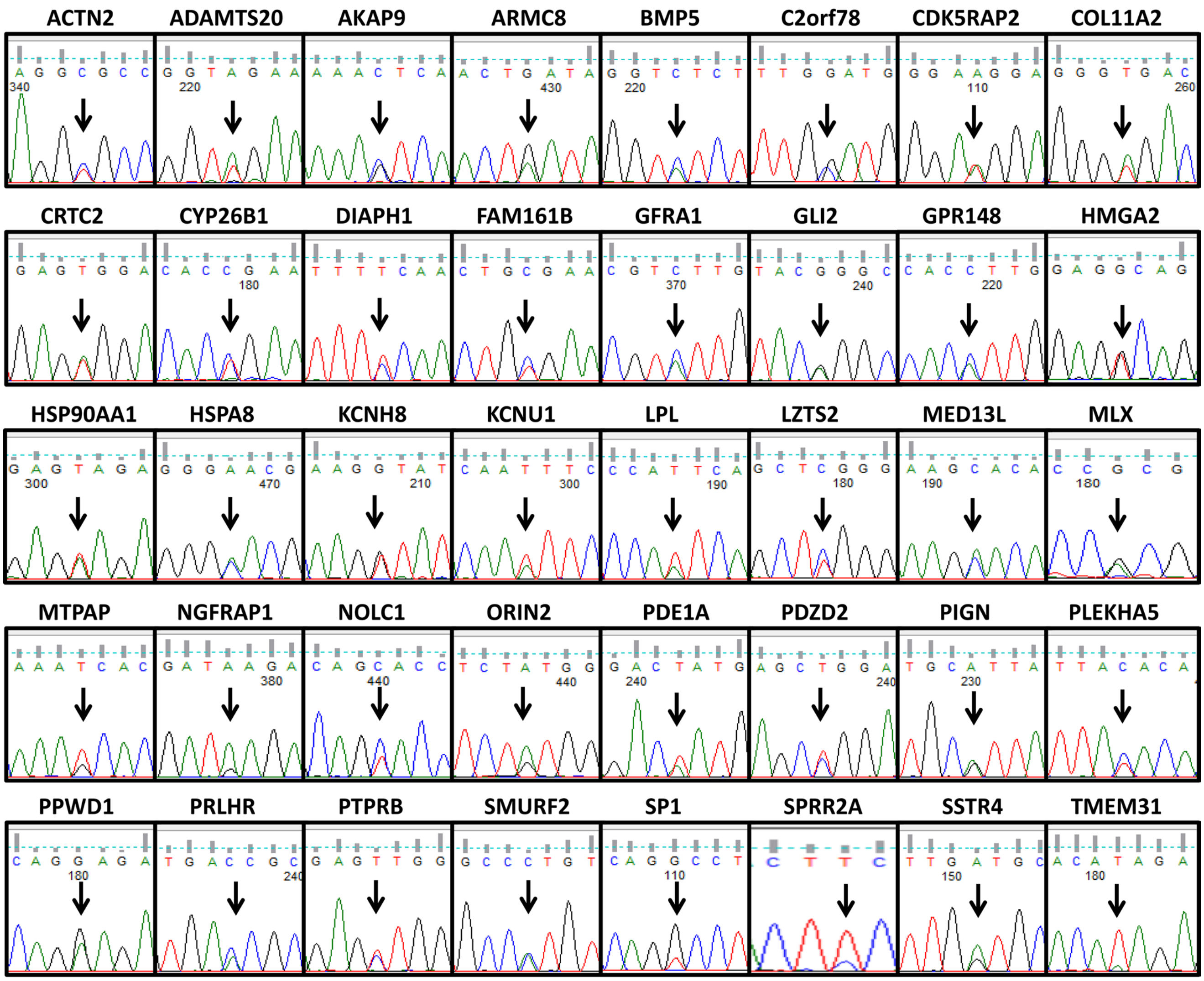
ACTN2
ADAMTS20
AKAP9
ARMC8
BMP5
C2orf78
CDK5RAP2
COL11A2
CRTC2
CYP26B1
DIAPH1
FAM161B
GFRA1
GLI2
GPR148
HMGA2
HSP90AA1
HSPA8
KCNH8
KCNU1
LPL
LZTS2
MED13L
MLX
MTPAP
NGFRAP1
NOLC1
ORIN2
PDE1A
PDZD2
PIGN
PLEKHA5
PPWD1
PRLHR
PTPRB
SMURF2
SP1
SPRR2A
SSTR4
TMEM31

P5T1

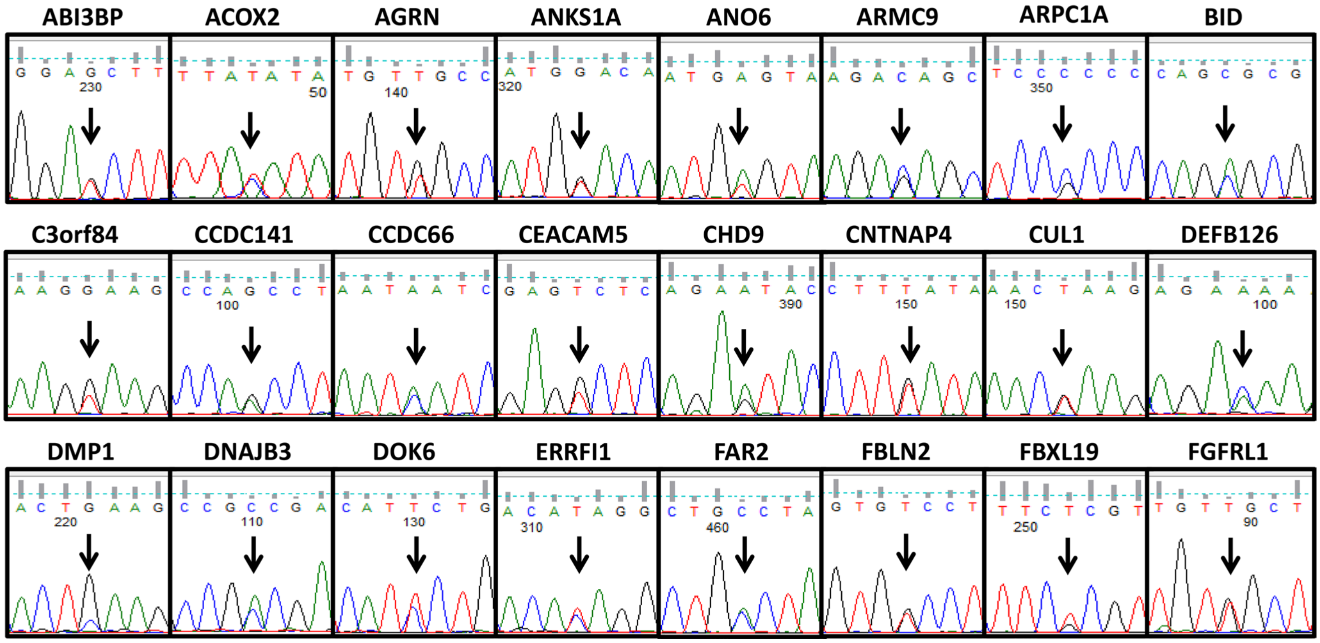
ABI3BP
ACOX2
AGRN
ANKS1A
ANO6
ARMC9
ARPC1A
BID
C3orf84
CCDC141
CCDC66
CEACAM5
CHD9
CNTNAP4
CUL1
DEFB126
DMP1
DNAJB3
DOK6
ERRFI1
FAR2
FBLN2
FBXL19
FGFRL1

P4T5

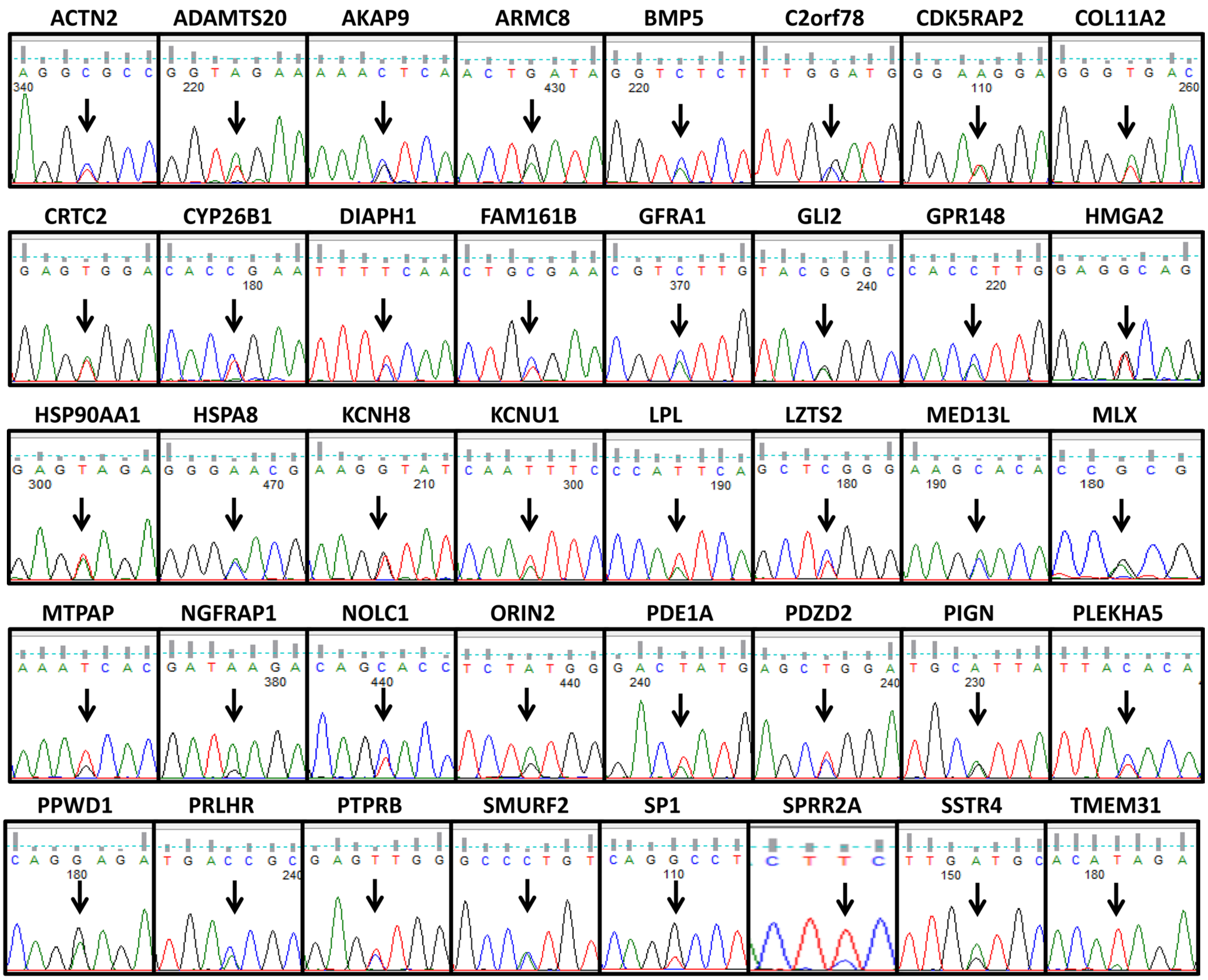
ACTN2
ADAMTS20
AKAP9
ARMC8
BMP5
C2orf78
CDK5RAP2
COL11A2
CRTC2
CYP26B1
DIAPH1
FAM161B
GFRA1
GLI2
GPR148
HMGA2
HSP90AA1
HSPA8
KCNH8
KCNU1
LPL
LZTS2
MED13L
MLX
MTPAP
NGFRAP1
NOLC1
ORIN2
PDE1A
PDZD2
PIGN
PLEKHA5
PPWD1
PRLHR
PTPRB
SMURF2
SP1
SPRR2A
SSTR4
TMEM31

P5T1

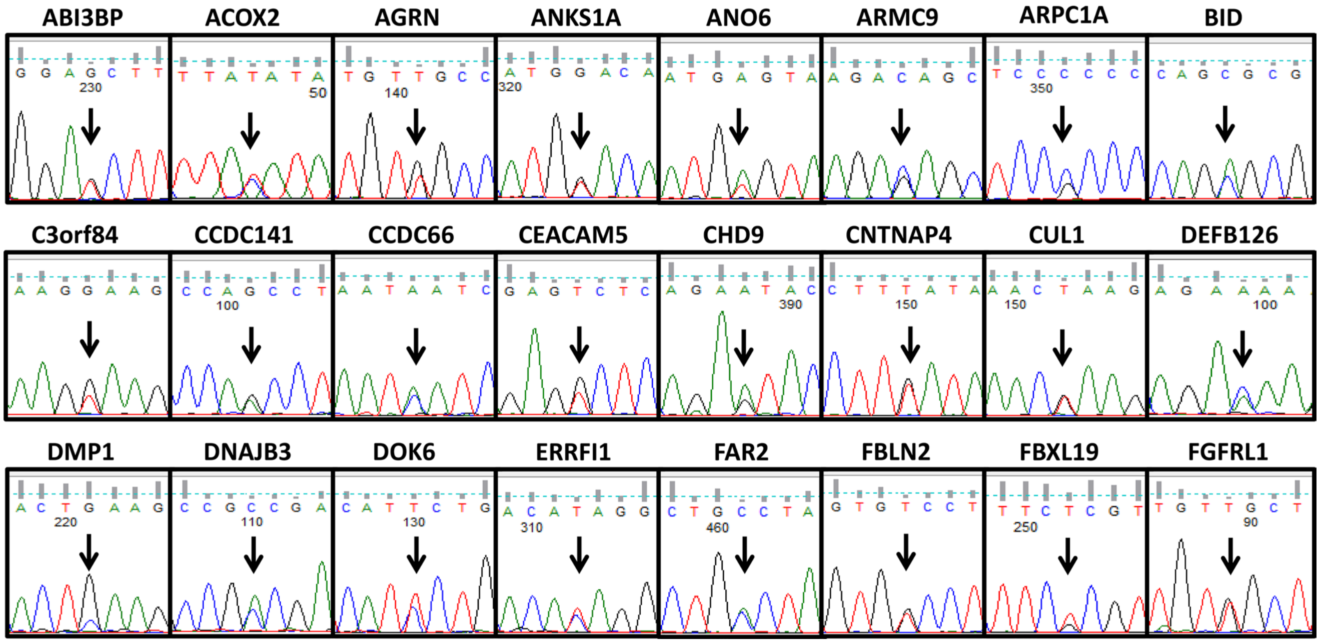
ABI3BP
ACOX2
AGRN
ANKS1A
ANO6
ARMC9
ARPC1A
BID
C3orf84
CCDC141
CCDC66
CEACAM5
CHD9
CNTNAP4
CUL1
DEFB126
DMP1
DNAJB3
DOK6
ERRFI1
FAR2
FBLN2
FBXL19
FGFRL1

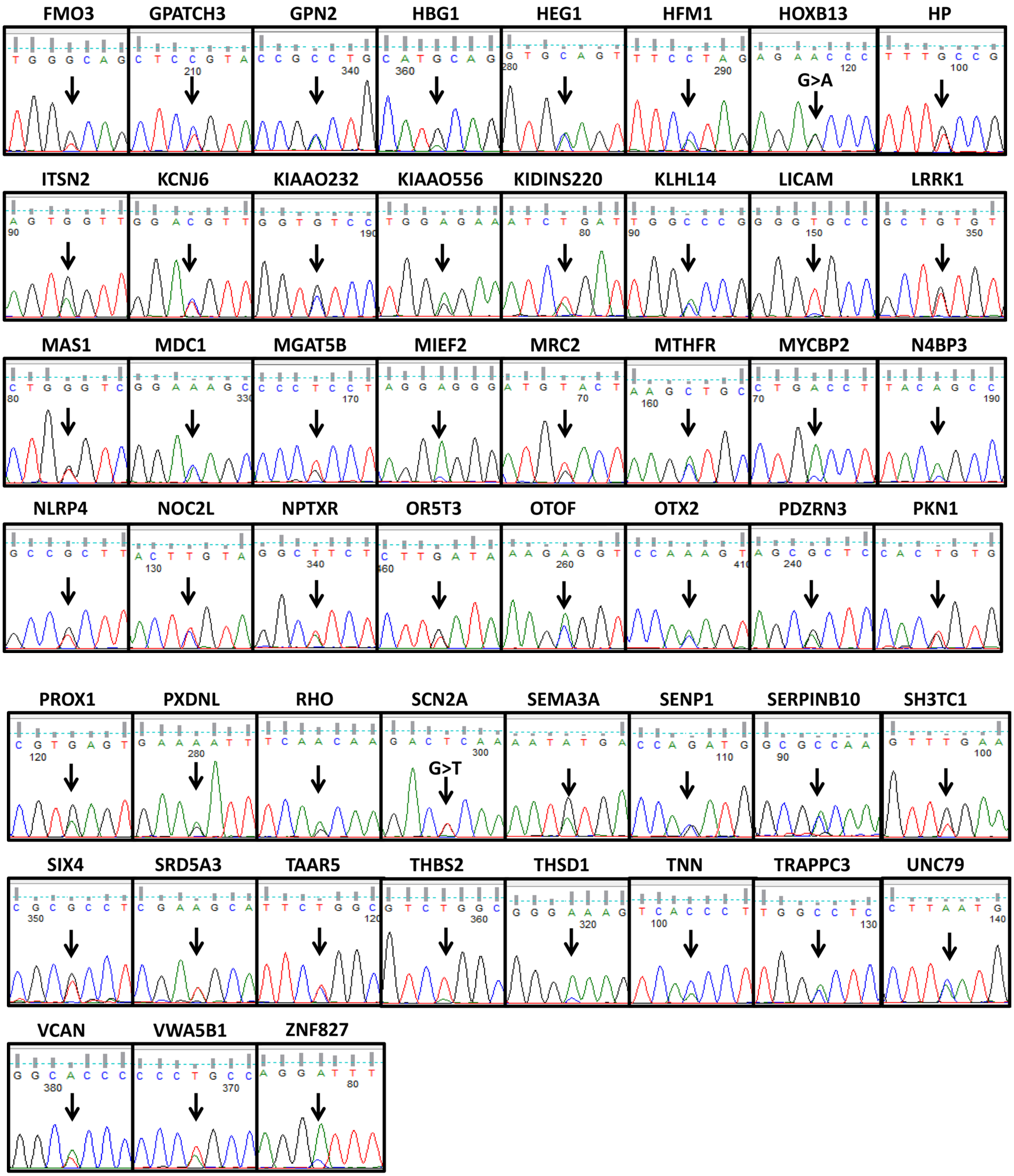
FMO3
GPATCH3
GPN2
HBG1
HEG1
HFM1
HOXB13
HP
G>A
ITSN2
KCNJ6
KIAAO232
KIAAO556
KIDINS220
KLHL14
LICAM
LRRK1
MAS1
MDC1
MGAT5B
MIEF2
MRC2
MTHFR
MYCBP2
N4BP3
NLRP4
NOC2L
NPTXR
OR5T3
OTOF
OTX2
PDZRN3
PKN1
PROX1
PXDNL
RHO
SCN2A
SEMA3A
SENP1
SERPINB10
SH3TC1
G>T
SIX4
SRD5A3
TAAR5
THBS2
THSD1
TNN
TRAPPC3
UNC79
VCAN
VWA5B1
ZNF827

P5T2

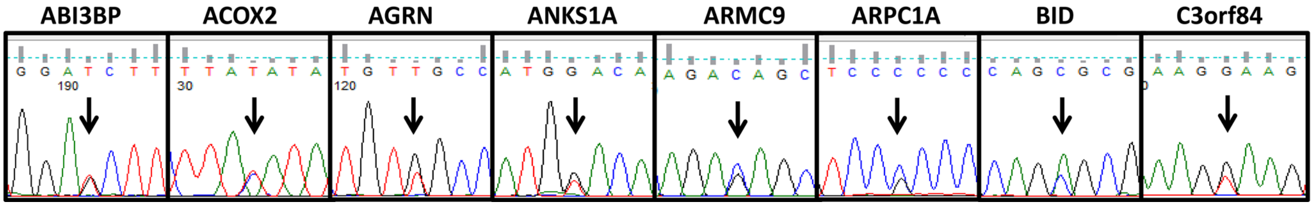
ABI3BP
ACOX2
AGRN
ANKS1A
ARMC9
ARPC1A
BID
C3orf84

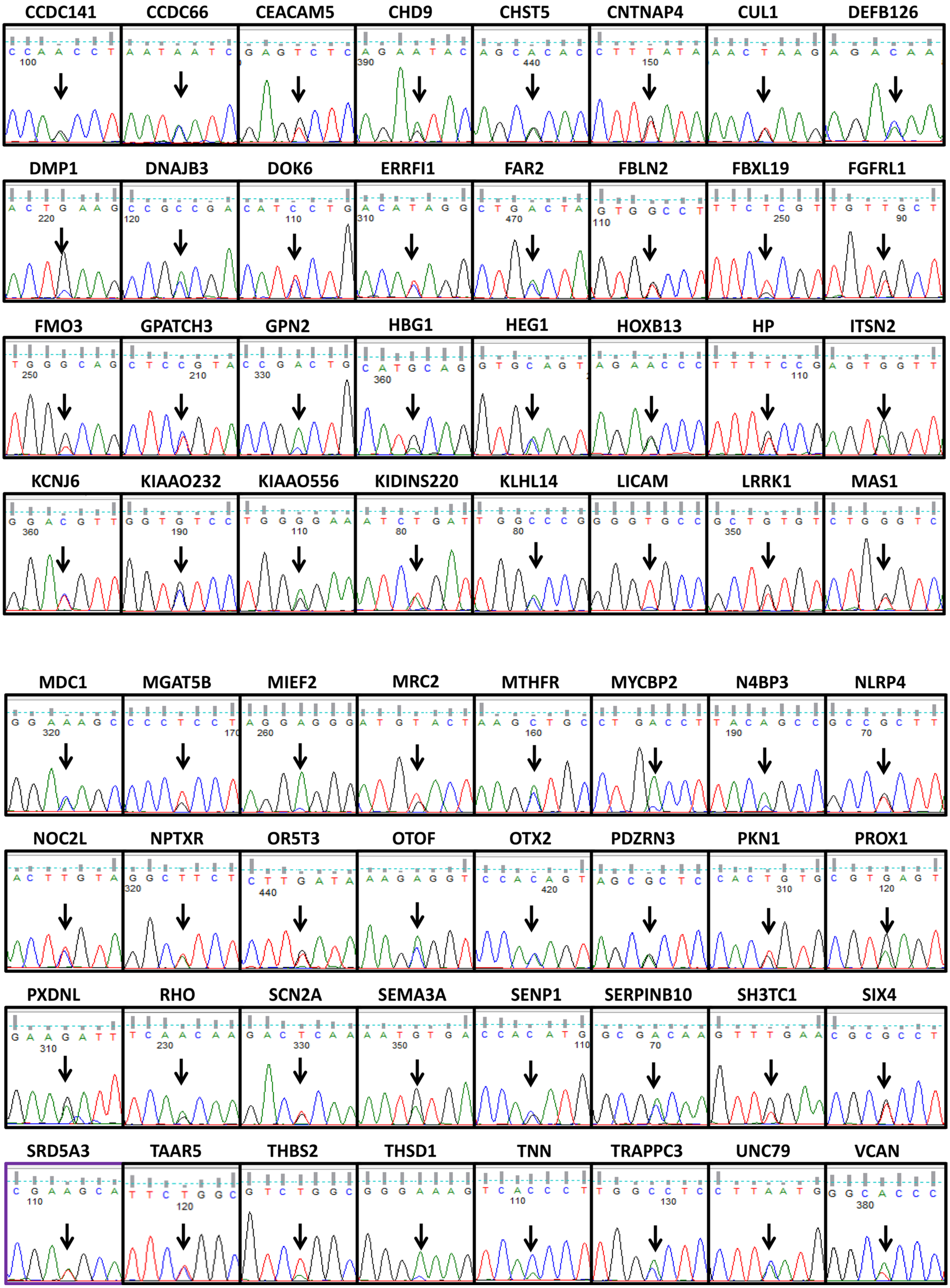
CCDC141
CCDC66
CEACAM5
CHD9
CHST5
CNTNAP4
CUL1
DEFB126
DMP1
DNAJB3
DOK6
ERRFI1
FAR2
FBLN2
FBXL19
FGFRL1
FMO3
GPATCH3
GPN2
HBG1
HEG1
HOXB13
HP
ITSN2
KCNJ6
KIAAO232
KIAAO556
KIDINS220
KLHL14
LICAM
LRRK1
MAS1
MDC1
MGAT5B
MIEF2
MRC2
MTHFR
MYCBP2
N4BP3
NLRP4
NOC2L
NPTXR
OR5T3
OTOF
OTX2
PDZRN3
PKN1
PROX1
PXDNL
RHO
SCN2A
SEMA3A
SENP1
SERPINB10
SH3TC1
SIX4
SRD5A3
TAAR5
THBS2
THSD1
TNN
TRAPPC3
UNC79
VCAN

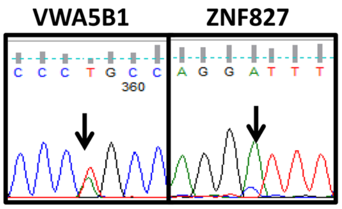
VWA5B1
ZNF827

P5T3

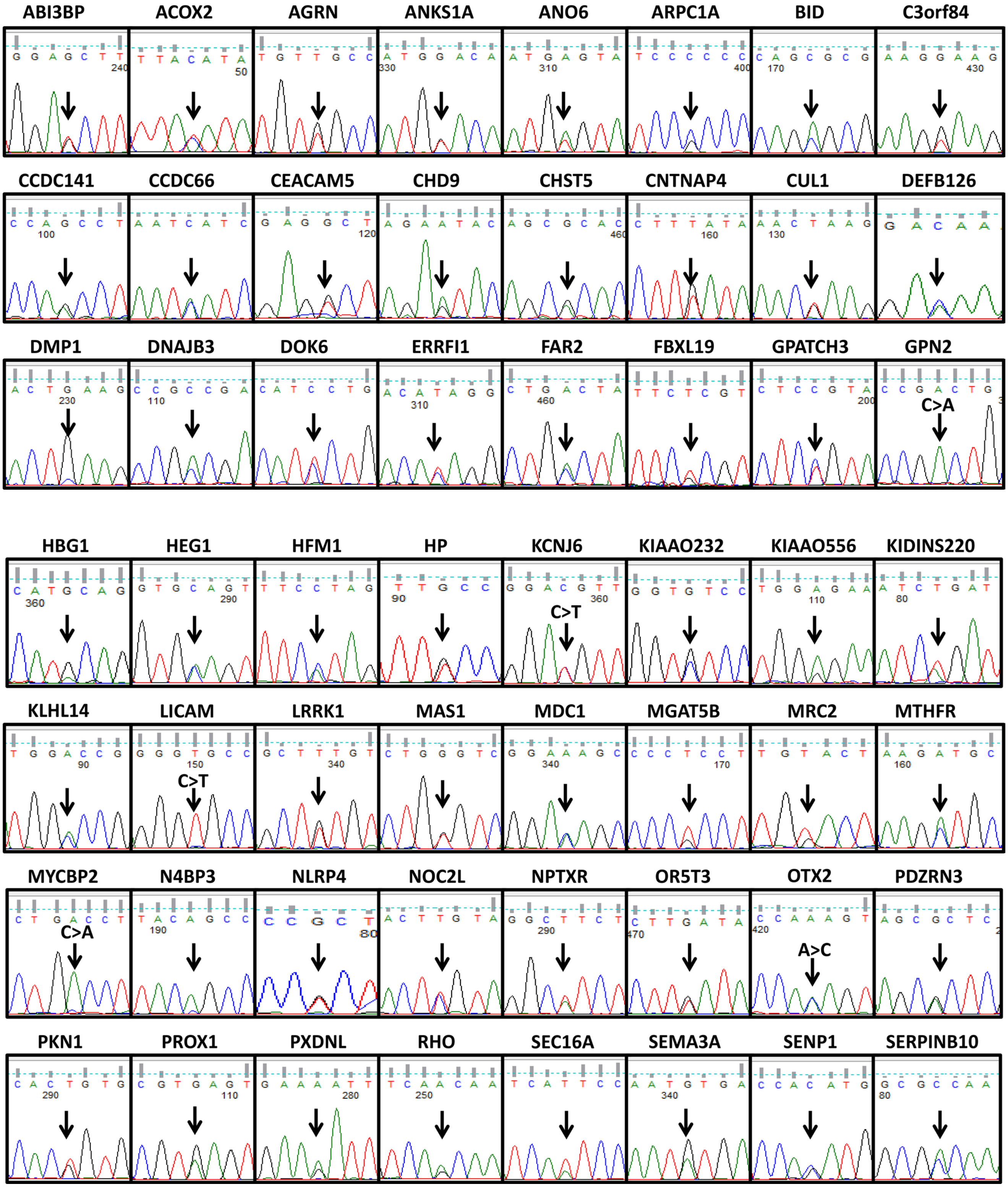
ABI3BP
ACOX2
AGRN
ANKS1A
ANO6
ARPC1A
BID
C3orf84
CCDC141
CCDC66
CEACAM5
CHD9
CHST5
CNTNAP4
CUL1
DEFB126
DMP1
DNAJB3
DOK6
ERRFI1
FAR2
FBXL19
GPATCH3
GPN2
C>A
HBG1
HEG1
HFM1
HP
KCNJ6
C>T
KIAAO232
KIAAO556
KIDINS220
KLHL14
LICAM
C>T
LRRK1
MAS1
MDC1
MGAT5B
MRC2
MTHFR
MYCBP2
C>A
N4BP3
NLRP4
NOC2L
NPTXR
OR5T3
OTX2
A>C
PDZRN3
PKN1
PROX1
PXDNL
RHO
SEC16A
SEMA3A
SENP1
SERPINB10

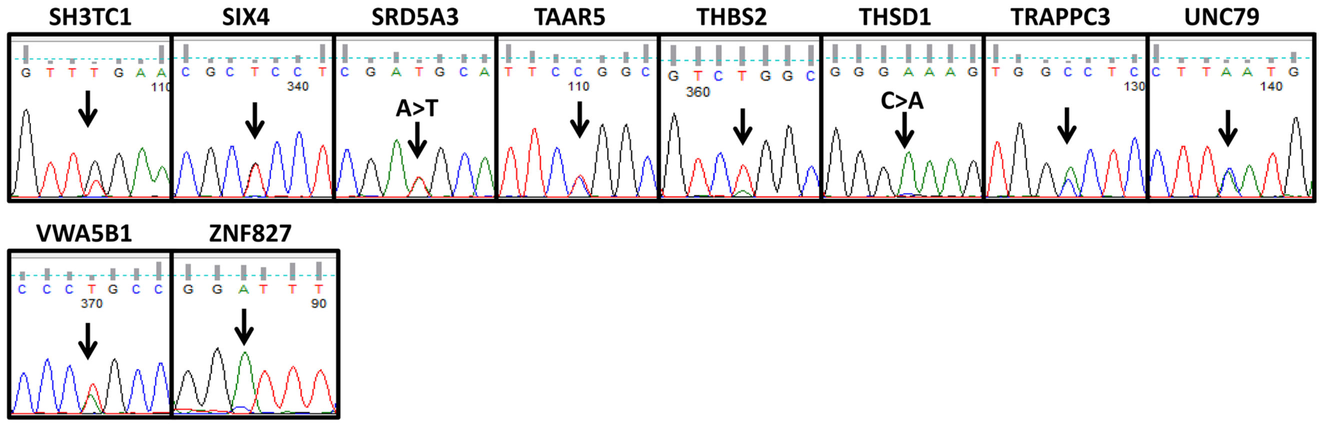
SH3TC1
SIX4
SRD5A3
A>T
TAAR5
THBS2
THSD1
C>A
TRAPPC3
UNC79
VWA5B1
ZNF827

P5T4

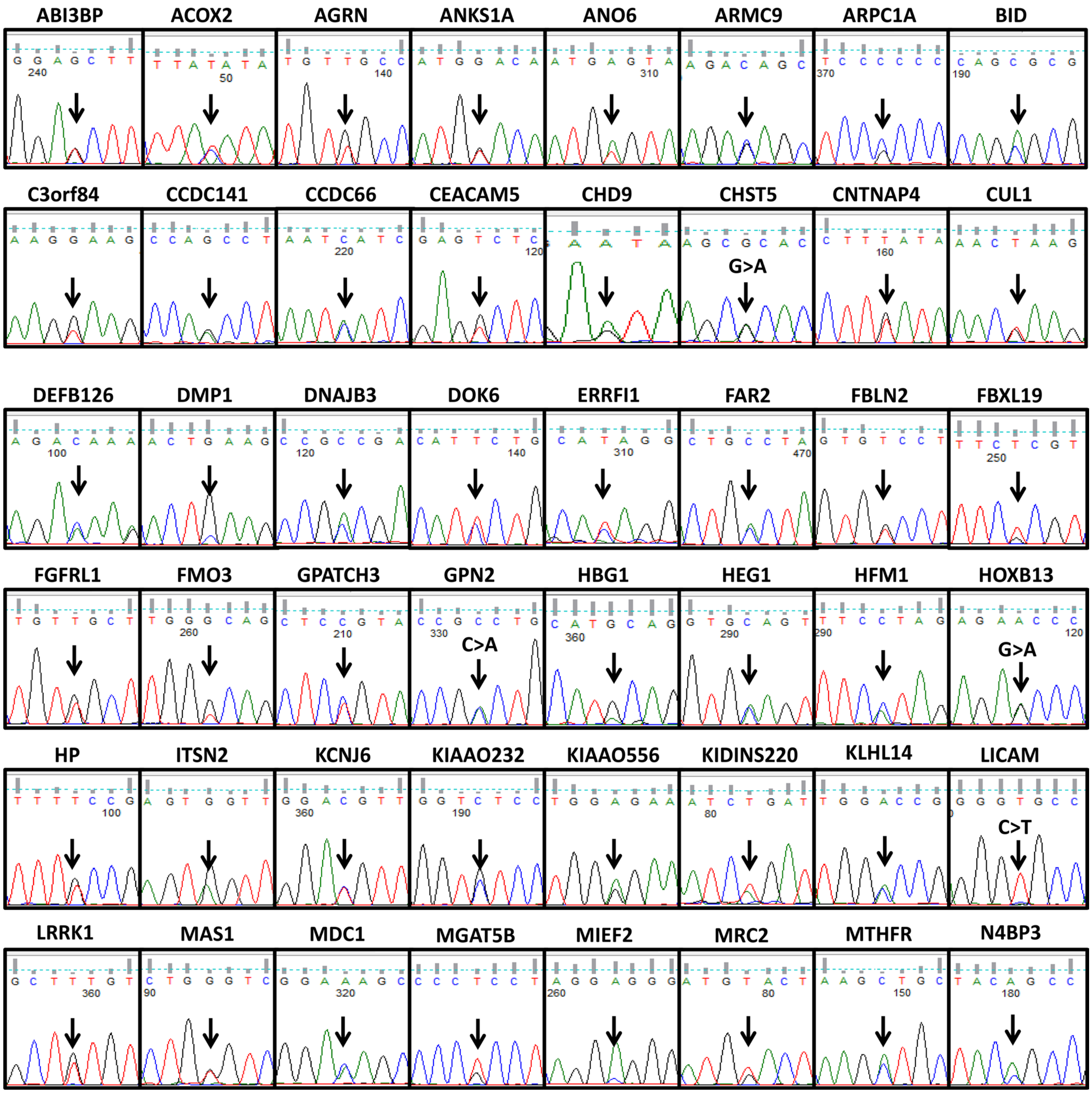
ABI3BP
ACOX2
AGRN
ANKS1A
ANO6
ARMC9
ARPC1A
BID
C3orf84
CCDC141
CCDC66
CEACAM5
CHD9
CHST5
G>A
CNTNAP4
CUL1
DEFB126
DMP1
DNAJB3
DOK6
ERRFI1
FAR2
FBLN2
FBXL19
FGFRL1
FMO3
GPATCH3
GPN2
C>A
HBG1
HEG1
HFM1
HOXB13
G>A
HP
ITSN2
KCNJ6
KIAAO232
KIAAO556
KIDINS220
KLHL14
LICAM
C>T
LRRK1
MAS1
MDC1
MGAT5B
MIEF2
MRC2
MTHFR
N4BP3

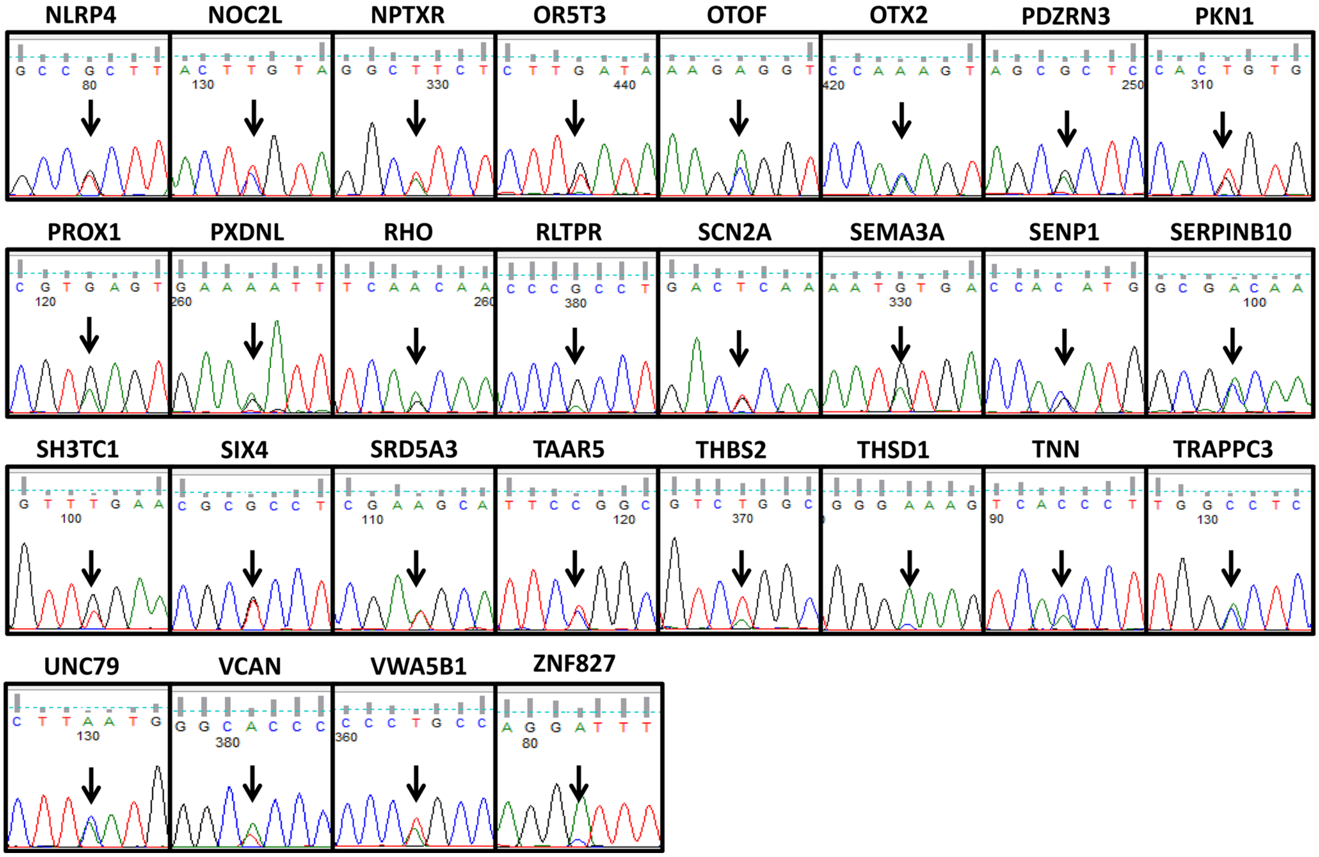
NLRP4
NOC2L
NPTXR
OR5T3
OTOF
OTX2
PDZRN3
PKN1
PROX1
PXDNL
RHO
RLTPR
SCN2A
SEMA3A
SENP1
SERPINB10
SH3TC1
SIX4
SRD5A3
TAAR5
THBS2
THSD1
TNN
TRAPPC3
UNC79
VCAN
VWA5B1
ZNF827

P5T5

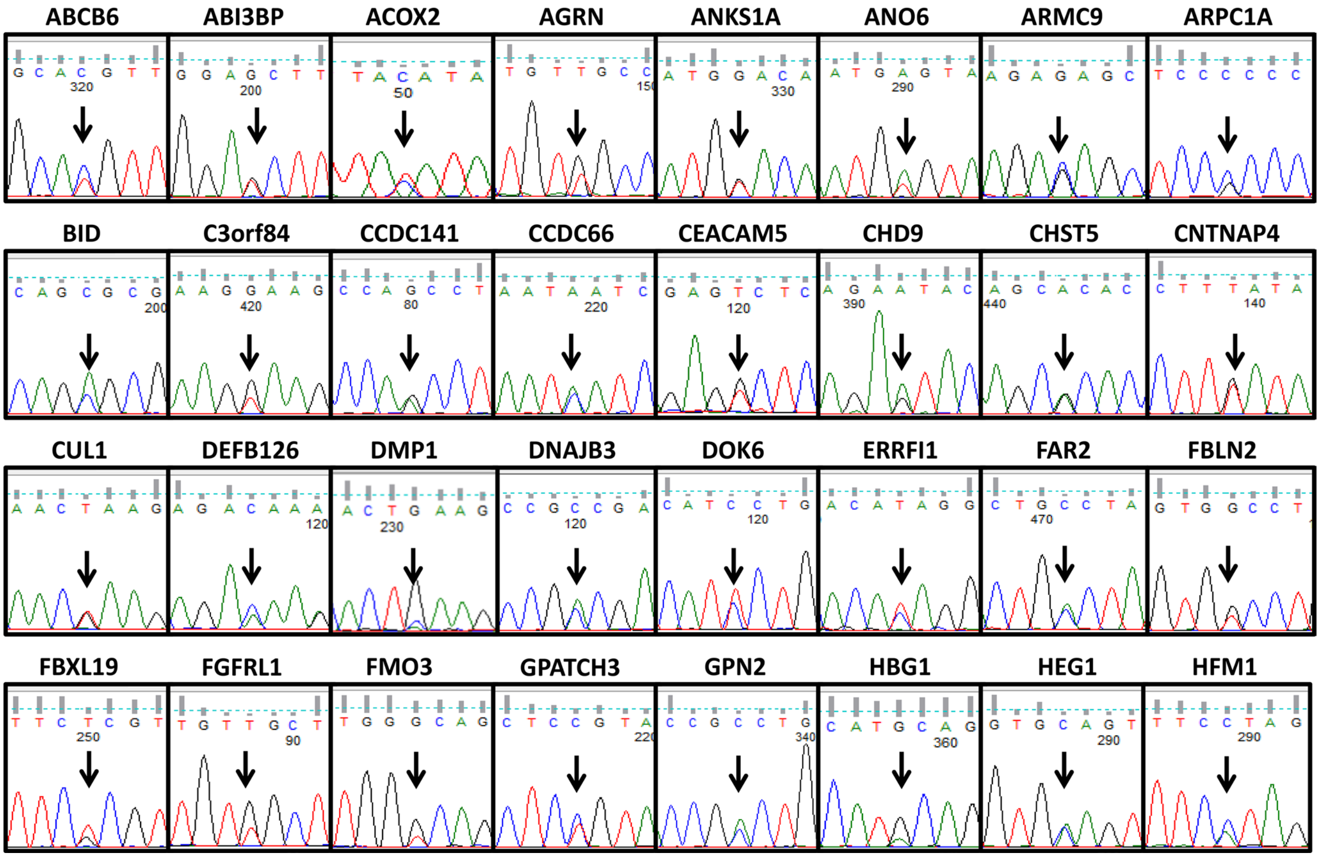
ABCB6
ABI3BP
ACOX2
AGRN
ANKS1A
ANO6
ARMC9
ARPC1A
BID
C3orf84
CCDC141
CCDC66
CEACAM5
CHD9
CHST5
CNTNAP4
CUL1
DEFB126
DMP1
DNAJB3
DOK6
ERRFI1
FAR2
FBLN2
FBXL19
FGFRL1
FMO3
GPATCH3
GPN2
HBG1
HEG1
HFM1

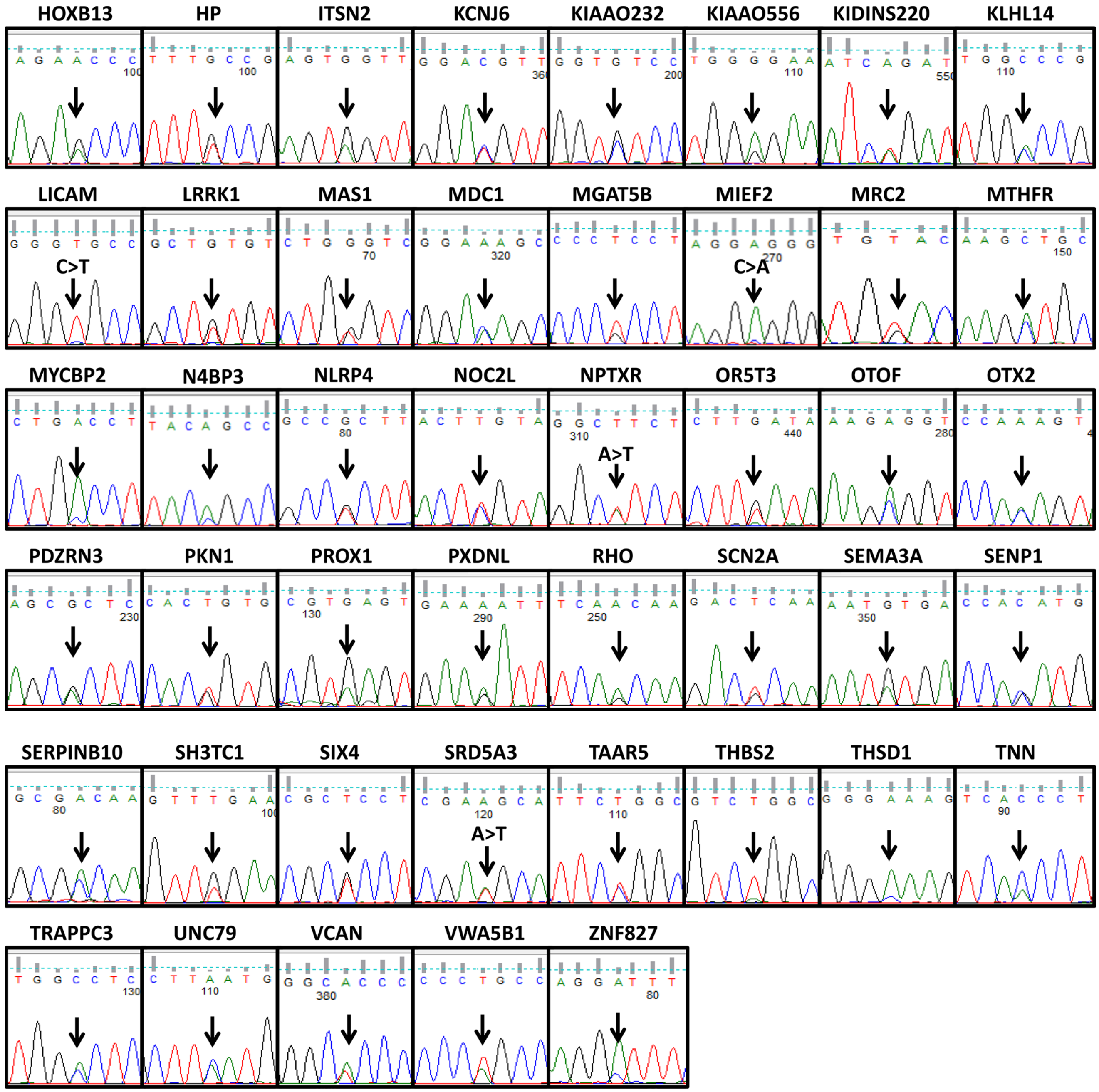

Supplementary Figure 3: Sanger sequencing validation of mutations identified by whole-exome sequencing in five regions of each tumor. Mutations are indicated with an arrow. The color of the border indicates whether the mutations were identified by NGS, black imply mutation was identified by NGS and Sanger sequencing, purple imply mutation was identified by Sanger sequencing, but not NGS. Sample names in the form of patient identification and lesion site, such as P1T1 represent tumor region 1 of patient 1.

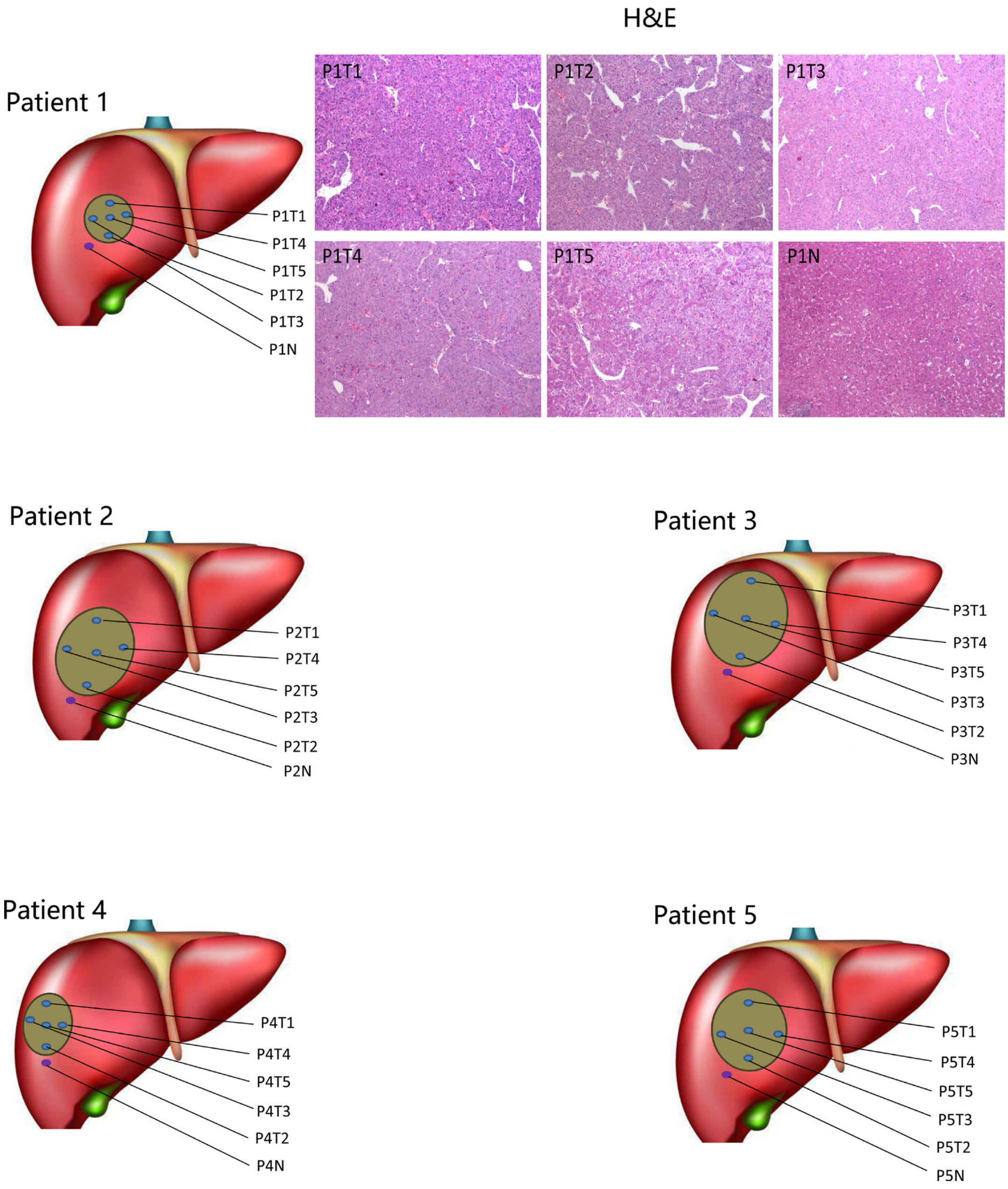

Supplementary Figure 4: Schematic of spatial distribution of five tumor regions in five PLC patients. The brown circle on behalf of the lesions, and the position of blue circles represent spatial distribution of five tumor specimens from the same tumor, adjacent normal tissues are indicated in purple. Sample names are marked on the right. HE staining of samples from patient 1 (×10) shown in the upper right.

Supplementary Table 1: TCRβ CDR3 sequencing metrics in five PLC patients

Patient	Region	Productive TCRβ reads	Unique TCRβ reads	Highest frequency clones (%)	Top 250 clones (%)	ShannonDI
Patient 1	T1	5360036	987605	3.86	29.13	27406.56
	T2	4392858	403867	2.17	38.51	7616.34
	T3	5167306	715877	4.21	33.14	14638.81
	T4	4662092	416706	2.03	35.86	8544.04
	T5	4096513	412691	3.14	40.93	7187.40
	N	3935084	332686	7.96	49.06	2879.83
	B	4141342	840685	2.52	19.81	57176.64
Patient 2	T1	3651721	518939	0.98	30.95	18096
	T2	5458844	664799	1.56	32.15	15372.41
	T3	5874530	711594	1.26	29.16	19349.52
	T4	5780239	592861	1.9	35.51	11369.95
	T5	4721377	608839	0.99	30.78	17516.28
	N	5437841	751031	1.64	24.90	24790.03
	B	4774968	806320	1.81	21.72	38342.37
Patient 3	T1	5791071	280919	6.89	63.24	1278.49
	T2	5721249	286599	4.79	61.1	1534.50
	T3	4506728	292206	7.43	56.47	2281.84
	T4	5802227	281650	7.37	62.43	1312.64
	T5	5818104	307188	5.05	57.78	2011.46
	N	5544156	380251	9.0	54.77	2215.72
	B	4260951	831968	6.51	24.86	37514.41
Patient 4	T1	5916386	613842	4.28	37.37	9356.73
	T2	4874918	431783	3.79	46.51	4786.17
	T3	3895671	370977	3.62	43.99	5218.94
	T4	5780239	584445	8.28	43.34	5233.54
	T5	5203030	400495	6.09	48.96	3541.83
	T1_RE	4999086	804699	3.28	31.31	9632.66
	T2_RE	4140723	591676	4.10	39.03	4917.59
	T3_RE	4339141	666721	2.62	34.67	6011.33
	T4_RE	5378560	797385	7.42	37.48	6192.03
	T5_RE	4515047	592156	4.70	40.72	4494.97
	N	4795596	507332	8.27	43.15	6010.56
	B	5998093	474193	45.53	71.31	234.52
Patient 5	T1	4538512	254916	8.64	58.68	1521.37
	T2	5578762	379031	22.56	61.27	949.64
	T3	4677002	285741	16.69	59.06	1317.05
	T4	5526088	429977	7.85	51.73	2593.43
	T5	5057312	320041	7.08	55.39	2202.19
	N	4382572	465318	7.1	42.40	6184.12
	B	7131196	1267771	1.91	18.55	57895.63

Supplementary Table 2: The distribution of TCRβ clones with different frequency

Patient	Region	Unique TCRB reads (n)					
		< 0.0001%	0.0001–0.001%	0.001–0.01%	0.01–0.1%	0.1–1%	> 1%
Patient 1	T1	92348	54922	8250	928	56	1
	T2	353327	43598	5585	1263	92	3
	T3	660022	47820	7006	963	62	4
	T4	354469	56554	4165	1405	110	2
	T5	365603	40223	5659	1099	101	5
	N	282919	44087	4466	1146	58	6
	B	754882	74349	10752	669	32	1
Patient 2	T1	451880	58714	7281	984	80	0
	T2	603907	52826	6644	1340	79	3
	T3	645580	57626	7168	1133	86	1
	T4	534404	49434	7704	1214	101	4
	T5	544798	54751	7956	1254	80	0
	N	684390	56252	8781	1544	63	1
	B	729994	65396	9884	1002	43	1
Patient 3	T1	248008	27512	4555	744	88	12
	T2	250548	30679	4520	724	99	11
	T3	251348	34939	4969	830	111	9
	T4	247193	29341	4212	801	90	13
	T5	267054	34784	4315	903	120	10
	N	341843	31999	5544	781	75	9
	B	743387	76398	11594	560	25	4
Patient 4	T1	557998	46921	7583	1260	75	5
	T2	383495	40549	6708	938	86	7
	T3	316280	47687	5977	942	81	10
	T4	540664	36203	6348	1156	67	7
	T5	361007	32133	6265	1002	80	8
	T1_RE	737552	57707	8307	1066	62	5
	T2_RE	539613	43855	7193	934	76	5
	T3_RE	606530	52771	6371	983	61	5
	T4_RE	740588	49616	6103	1018	54	6
	T5_RE	538252	45968	6863	998	68	7
	N	455048	44726	6622	865	67	4
	B	438425	31298	4113	299	56	2
Patient 5	T1	216861	33048	3940	954	104	9
	T2	341425	32382	4413	723	82	6
	T3	245995	34909	3797	940	93	7
	T4	384494	39659	4915	830	72	7
	T5	283815	30732	4562	825	95	9
	N	415023	42159	7090	927	70	4
	B	1191750	64476	10811	737	43	1

Supplementary Table 3: comparison of duplicate samples of each tumor region from patient 4

Sample ID[a]	The most expensive clones	Common T cell clones of the duplicate samples (%)	R	*P*
P4T1 vs P4T1_RE	100	88 (88.0)	0.986	2.2e-16
	250	222 (88.8)	0.987	2.2e-16
	500	442 (88.4)	0.988	2.2e-16
	1000	864 (86.4)	0.988	2.2e-16
	2500	2330 (93.2)	0.988	2.2e-16
	5000	4515 (90.3)	0.989	2.2e-16
	7500	6652 (88.7)	0.989	2.2e-16
	10000	8974 (89.74)	0.989	2.2e-16
P4T2 vs P4T2_RE	100	95 (95.0)	0.981	2.2e-16
	250	230 (92.0)	0.983	2.2e-16
	500	446 (89.2)	0.984	2.2e-16
	1000	921 (92.1)	0.985	2.2e-16
	2500	2253 (90.1)	0.985	2.2e-16
	5000	4421 (88.4)	0.985	2.2e-16
	7500	6754 (90.1)	0.985	2.2e-16
	10000	8908 (89.1)	0.986	2.2e-16
P4T3 vs P4T3_RE	100	85 (85.0)	0.982	2.2e-16
	250	221 (88.4)	0.984	2.2e-16
	500	449 (89.8)	0.985	2.2e-16
	1000	916 (91.6)	0.986	2.2e-16
	2500	2222 (88.9)	0.986	2.2e-16
	5000	4482 (89.6)	0.987	2.2e-16
	7500	6623 (88.3)	0.987	2.2e-16
	10000	8459 (84.6)	0.987	2.2e-16
P4T4_1 vs P4T4_2	100	87 (87.0)	0.999	2.2e-16
	250	228 (91.2)	0.999	2.2e-16
	500	455 (91.0)	0.999	2.2e-16
	1000	880 (88.0)	0.999	2.2e-16
	2500	2253 (90.1)	0.999	2.2e-16
	5000	4368 (87.4)	0.999	2.2e-16
	7500	6330 (84.4)	0.999	2.2e-16
	10000	8420 (84.2)	0.999	2.2e-16
P4T5_1 vs P4T5_2	100	93 (93.0)	0.993	2.2e-16
	250	227 (90.8)	0.994	2.2e-16
	500	451 (90.2)	0.994	2.2e-16
	1000	915 (91.5)	0.994	2.2e-16
	2500	2257 (90.28)	0.994	2.2e-16
	5000	4413 (88.3)	0.994	2.2e-16
	7500	6674 (88.9)	0.994	2.2e-16
	10000	8883 (88.8)	0.994	2.2e-16

[a]P: patient, T: tumor, RE:duplicated sample, T1 and T1_RE are duplicate samples of T1, P4T1 represent tumor region 1 of patient 4.

Supplementary Table 4: Exome-capture sequencing statistics in five PLC patients

Sample ID[a]	Total clean reads count	Fraction of uniquely mapped on target	Average sequencing depth on target
P1T1	65,814,500	98.3%	102.71
P1T2	66,880,972	98.67%	104.37
P1T3	65,419,166	98.78%	101.8
P1T4	84,390,186	98.4%	116.47
P1T5	112,964,310	98.31%	148.57
P1B	86,212,742	98.5%	132.34
P2T1	85,105,156	98.46%	129.98
P2T2	76,539,942	98.64%	114.33
P2T3	73,344,058	99%	114.88
P2T4	58,418,786	97.94%	91.18
P2T5	81,787,546	98.31%	110.4
P2B	86,984,966	97.56%	138.11
P3T1	80,146,504	98.99%	127.41
P3T2	86,694,488	98.97%	137.09
P3T3	80,690,118	98.85%	128.22
P3T4	82,881,802	98.84%	130.52
P3T5	88,595,180	98.64%	136.84
P3B	78,039,534	98.53%	126
P4T1	90,428,994	99.07%	142.8
P4T2	76,947,750	98.58%	117.86
P4T3	84,269,948	98.52%	128.47
P4T4	107,154,858	99.02%	168.67
P4T5	107,965,250	98.68%	165.7
P4B	87,596,086	97.23%	135.55
P5T1	83,422,780	98.89%	128.31
P5T2	75,018,196	98.74%	116.96
P5T3	75,814,054	98.89%	117.77
P5T4	66,171,166	98.72%	105.98
P5T5	100,051,398	98.35%	155.22
P5B	83,748,418	97.85%	131.64

[a]P: patient, T: tumor, B: peripheral blood, P1T1 represent tumor region 1 of patient 1.

Supplementary Table 5: Summary of non-synonymous somatic mutations identified in the five PLC patients. See Supplementary_Table_5

Supplementary Table 6: Mutations verification by Sanger sequencing in all the patients

Patient	P1	P2	P3	P4	P5
N0. of successfully sequenced mutations	98	91	67	68	106
N0. of mutations were selection to verify	75	70	52	56	80
Total N0. of samples for verifying	375	350	260	280	400
Total No. of successfully verified samples	368	341	247	276	384
Verification rate %	98.13%	97.43%	95%	98.57%	96%
Average verification rate	97.03%				

Supplementary Table 7: TCR sequencing primers. See Supplementary_Table_7
