# Supplementary material for: Multi-omics study revealing the complexity and spatial heterogeneity of tumor-infiltrating lymphocytes in primary liver carcinoma
Source: Oncotarget. 2017 Mar 31;8(21):34844–57. doi: 10.18632/oncotarget.16758 (PMC5471016; doi:10.18632/oncotarget.16758)
Supplement: Supplementary file 2 [file oncotarget-08-34844-s002.docx]

**Supplementary Table S5: Summary of non-synonymous somatic mutations identified in the five PLC patients**

**Patient1**

| **Gene** | **Chr** | **Position** | **Nucleotide**  **Variant** | **Amino**  **Acid Change** | **Regions found**  **Mutated by NGS (P1T1/P1T2/P1T3**  **/P1T4/P1T5)** | **Regions found**  **Mutated by SS**  **(P1T1/P1T2/P1T3**  **/P1T4/P1T5)** |
| --- | --- | --- | --- | --- | --- | --- |
| ADAMTS1 | chr21 | 28212022 | C>G | G638R | 0 / 1 / 0 / 0 / 1 | 0 / 1 / 0 / 0 / 1 |
| ADCY1 | chr7 | 45650091 | T>A | N301K | 1 / 1 / 1 / 1 / 1 | NA |
| ADGRL1 | chr19 | 14263364 | G>A | A1167V | 1 / 1 / 1 / 1 / 1 | 1 / 1 / 1 / 1 / 1 |
| AK9 | chr6 | 109867081 | T>A | T1072S | 1 / 1 / 1 / 1 / 1 | NA |
| ANKRD12 | chr18 | 9257871 | G>A | D1513N | 0 / 0 / 0 / 1 / 0 | 0 / 0 / 0 / 1 / 0 |
| APCDD1 | chr18 | 10471673 | T>A | L130H | 0 / 1 / 0 / 0 / 0 | 0 / 1 / 0 / 0 / 0 |
| ASTN2 | chr9 | 119568074 | G>T | P694T | 1 / 1 / 1 / 1 / 1 | 1 / 1 / 1 / 1 / 1 |
| BBS10 | chr12 | 76739792 | T>A | Y658F | 1 / 1 / 1 / 1 / 1 | 1 / 1 / 1 / 1 / 1 |
| BCL11B | chr14 | 99640894 | A>T | L759Q | 1 / 1 / 1 / 1 / 1 | NA |
| CACNA1E | chr1 | 181732558 | G>A | G1569D | 1 / 1 / 1 / 1 / 1 | 1 / 1 / 1 / 1 / 1 |
| CACNA1E | chr1 | 181765953 | A>T | R2120W | 1 / 1 / 1 / 1 / 1 | 1 / 1 / 1 / 1 / 1 |
| CALM3 | chr19 | 47112398 | G>C | .M146I | 1 / 0 / 0 / 0 / 0 | 1 / 0 / 0 / 0 / 0 |
| CBS | chr21 | 44488657 | T>C | N93S | 1 / 1 / 1 / 1 / 1 | 1 / 1 / 1 / 1 / 1 |
| CCDC129 | chr7 | 31614244 | G>A | M172I | 1 / 1 / 0 / 1 / 1 | NA |
| CD180 | chr5 | 66480084 | A>T | L196Q | 1 / 1 / 1 / 1 / 1 | 1 / 1 / 1 / 1 / 1 |
| CFHR5 | chr1 | 196971654 | A>G | N397S | 1 / 1 / 1 / 1 / 1 | 1 / 1 / 1 / 1 / 1 |
| CLIC6 | chr21 | 36042492 | A>G | S269G | 0 / 1 / 0 / 0 / 0 | NA |
| CMA1 | chr14 | 24976619 | C>A | C51F | 1 / 1 / 1 / 1 / 1 | 1 / 1 / 1 / 1 / 1 |
| COL28A1 | chr7 | 7514263 | C>A | G424V | 1 / 1 / 1 / 1 / 1 | NA |
| CORO1B | chr11 | 67207597 | C>G | E333D | 1 / 1 / 1 / 1 / 1 | 1 / 1 / 1 / 1 / 1 |
| CREB3L2 | chr7 | 137612929 | T>A | T96S | 0 / 1 / 1 / 1 / 1 | NA |
| CT47A7 | chrX | 120088532 | C>G | A261P | 1 / 0 / 0 / 0 / 0 | 1 / 0 / 0 / 0 / 0 |
| DAZ2 | chrY | 25419044 | C>A | T419N | 0 / 0 / 1 / 0 / 0 | NA |
| DNAH14 | chr1 | 225580110 | T>A | V4381E | 1 / 1 / 1 / 1 / 1 | 1 / 1 / 1 / 1 / 1 |
| DST | chr6 | 56600061 | T>G | T40P | 1 / 1 / 1 / 1 / 1 | NA |
| EPHA6 | chr3 | 96706804 | A>C | T361P | 1 / 1 / 1 / 1 / 1 | 1 / 1 / 1 / 1 / 1 |
| FAM157B | chr9 | 141121527 | G>A | G354D | 1 / 0 / 0 / 1 / 0 | 1 / **1** / **1** / 1 / **1** |
| FAM227B | chr15 | 49800474 | T>A | S316C | 1 / 1 / 1 / 1 / 1 | 1 / 1 / 1 / 1 / 1 |
| FBN3 | chr19 | 8131027 | G>A | R2736W | 1 / 1 / 1 / 1 / 1 | 1 / 1 / 1 / 1 / 1 |
| FBXW8 | chr12 | 117426580 | A>T | Y382F | 1 / 1 / 1 / 1 / 1 | 1 / 1 / 1 / 1 / 1 |
| FLG2 | chr1 | 152326044 | A>T | H1406Q | 0 / 0 / 1 / 0 / 0 | 0 / 0 / 1 / 0 / 0 |
| FUT5 | chr19 | 5867057 | T>A | Q227 | 1 / 1 / 1 / 1 / 1 | 1 / 1 / 1 / 1 / 1 |
| GAGE12E | chrX | 49330181 | A>G | M97V | 0 / 1 / 1 / 0 / 0 | NA |
| GLI1 | chr12 | 57863284 | A>G | N460S | 0 / 0 / 1 / 0 / 0 | 0 / 0 / 1 / 0 / 0 |
| GOLGA6L10 | chr15 | 83016288 | C>T | V50I | 0 / 1 / 0 / 0 / 0 | NA |
| GOLGA6L2 | chr15 | 23686253 | T>C | K457E | 1 / 0 / 0 / 1 / 0 | NA |
| GRIA1 | chr5 | 153056702 | T>C | I268T | 1 / 1 / 1 / 1 / 1 | NA |
| HAPLN4 | chr19 | 19369476 | G>A | R225W | 1 / 1 / 1 / 1 / 1 | 1 / 1 / 1 / 1 / 1 |
| HIVEP3 | chr1 | 42045635 | T>A | I1612F | 1 / 1 / 1 / 1 / 1 | 1 / 1 / 1 / 1 / 1 |
| HMGN2 | chr1 | 26800610 | A>T | K42I | 1 / 0 / 1 / 1 / 1 | NA |
| HSD3B2 | chr1 | 119964509 | G>A | G129R | 0 / 0 / 0 / 1 / 0 | 0 / 0 / 0 / 1 / 0 |
| HSPA12B | chr20 | 3730468 | C>A | H336N | 1 / 1 / 1 / 1 / 1 | 1 / 1 / 1 / 1 / 1 |
| IL12RB2 | chr1 | 67787428 | G>A | D74N | 1 / 1 / 1 / 1 / 1 | 1 / 1 / 1 / 1 / 1 |
| INTS3 | chr1 | 153745175 | C>T | A961V | 1 / 1 / 1 / 1 / 1 | 1 / 1 / 1 / 1 / 1 |
| ISG15 | chr1 | 949647 | A>T | Y96F | 1 / 1 / 1 / 1 / 1 | 1 / 1 / 1 / 1 / 1 |
| KANSL3 | chr2 | 97276859 | T>C | Y368C | 1 / 1 / 1 / 1 / 1 | 1 / 1 / 1 / 1 / 1 |
| LRGUK | chr7 | 133868540 | A>T | E423D | 1 / 1 / 1 / 1 / 1 | 1 / 1 / 1 / 1 / 1 |
| LRRIQ3 | chr1 | 74648431 | G>T | P122T | 1 / 1 / 1 / 1 / 1 | 1 / 1 / 1 / 1 / 1 |
| MAP3K7 | chr6 | 91228218 | C>G | pA503P | 1 / 1 / 0 / 0 / 1 | 1 / 1 / 0 / 0 / 1 |
| MAZ | chr16 | 29818321 | C>A | T72K | 0 / 0 / 1 / 0 / 0 | NA |
| MROH2B | chr5 | 41058242 | G>A | R227C | 1 / 1 / 1 / 1 / 1 | 1 / 1 / 1 / 1 / 1 |
| MYH15 | chr3 | 108110647 | A>T | L1817Q | 1 / 1 / 1 / 1 / 1 | 1 / 1 / 1 / 1 / 1 |
| MYO1G | chr7 | 45007484 | T>A | S550C | 1 / 1 / 1 / 1 / 1 | NA |
| NACC2 | chr9 | 138942282 | T>A | Y29F | 1 / 1 / 1 / 1 / 1 | 1 / 1 / 1 / 1 / 1 |
| NEURL3 | chr2 | 97165130 | C>G | V191L | 1 / 1 / 1 / 1 / 1 | 1 / 1 / 1 / 1 / 1 |
| NPAP1 | chr15 | 24921054 | C>T | R14C | 1 / 1 / 1 / 1 / 1 | 1 / 1 / 1 / 1 / 1 |
| NPAP1 | chr15 | 24922021 | T>A | L336Q | 1 / 1 / 1 / 1 / 1 | 1 / 1 / 1 / 1 / 1 |
| NPIPB5 | chr16 | 22546799 | G>A | R832Q | 0 / 0 / 0 / 1 / 0 | NA |
| NR4A3 | chr9 | 102606975 | T>A | H433Q | 1 / 1 / 1 / 1 / 1 | 1 / 1 / 1 / 1 / 1 |
| NYNRIN | chr14 | 24886500 | C>A | P1849T | 1 / 1 / 1 / 1 / 1 | 1 / 1 / 1 / 1 / 1 |
| OCA2 | chr15 | 28090189 | G>C | T783R | 0 / 0 / 0 / 1 / 0 | 0 / 0 / 0 / 1 / 0 |
| OCSTAMP | chr20 | 45169951 | T>A | R555W | 1 / 1 / 1 / 1 / 1 | 1 / 1 / 1 / 1 / 1 |
| OR6P1 | chr1 | 158532686 | C>T | A237T | 1 / 1 / 1 / 1 / 1 | 1 / 1 / 1 / 1 / 1 |
| OTOP3 | chr17 | 72942971 | C>A | P341T | 1 / 1 / 1 / 1 / 1 | 1 / 1 / 1 / 1 / 1 |
| PCDHB6 | chr5 | 140531970 | T>A | L711Q | 1 / 1 / 1 / 1 / 1 | 1 / 1 / 1 / 1 / 1 |
| PDE4C | chr19 | 18343839 | C>T | G55S | 1 / 1 / 1 / 1 / 1 | 1 / 1 / 1 / 1 / 1 |
| PDE6A | chr5 | 149314261 | A>T | F165L | 1 / 1 / 1 / 1 / 1 | 1 / 1 / 1 / 1 / 1 |
| PDS5A | chr4 | 39927465 | G>A | S173L | 1 / 1 / 1 / 1 / 1 | NA |
| PHLDB2 | chr3 | 111603698 | G>T | R258S | 1 / 1 / 1 / 1 / 1 | 1 / 1 / 1 / 1 / 1 |
| PKHD1L1 | chr8 | 110530633 | G>A | S3976N | 1 / 1 / 1 / 1 / 1 | 1 / 1 / 1 / 1 / 1 |
| PRDM2 | chr1 | 14105510 | G>T | R407L | 1 / 1 / 1 / 1 / 1 | 1 / 1 / 1 / 1 / 1 |
| PREX2 | chr8 | 68950466 | A>G | I260V | 1 / 1 / 1 / 1 / 1 | NA |
| PXDC1 | chr6 | 3727881 | T>A | N161I | 1 / 1 / 1 / 1 / 1 | 1 / 1 / 1 / 1 / 1 |
| RAB3IP | chr12 | 70150441 | A>T | R186W | 1 / 1 / 0 / 1 / 1 | NA |
| RAPGEF1 | chr9 | 134464361 | C>G | L791F | 0 / 0 / 0 / 0 / 1 | 0 / 0 / 0 / 0 / 1 |
| RGS12 | chr4 | 3318315 | G>A | E140K | 0 / 0 / 0 / 0 / 1 | 0 / 0 / 0 / 0 / 1 |
| RPAP1 | chr15 | 41815529 | C>G | W820C | 0 / 0 / 0 / 1 / 0 | 0 / 0 / 0 / 1 / 0 |
| SB01 | chr12 | 123804451 | T>A | D932V | 1 / 1 / 1 / 1 / 1 | 1 / 1 / 1 / 1 / 1 |
| SIPA1L2 | chr1 | 232649717 | C>A | V457F | 1 / 1 / 1 / 1 / 1 | 1 / 1 / 1 / 1 / 1 |
| SLC27A2 | chr15 | 50518273 | A>T | K366I | 1 / 1 / 1 / 1 / 1 | 1 / 1 / 1 / 1 / 1 |
| SLC39A12 | chr10 | 18254508 | A>T | T214S | 1 / 1 / 1 / 1 / 1 | 1 / 1 / 1 / 1 / 1 |
| SLC9C2 | chr1 | 173552720 | A>T | L189M | 1 / 1 / 1 / 1 / 1 | 1 / 1 / 1 / 1 / 1 |
| SLCO6A1 | chr5 | 101834361 | C>G | G63A | 1 / 1 / 1 / 1 / 1 | 1 / 1 / 1 / 1 / 1 |
| SLITRK2 | chrX | 144905376 | T>A | L478Q | 1 / 1 / 1 / 1 / 1 | NA |
| SOGA1 | chr20 | 35431296 | T>A | E863V | 1 / 1 / 1 / 1 / 1 | 1 / 1 / 1 / 1 / 1 |
| STARD9 | chr15 | 42979423 | G>A | A1883T | 1 / 1 / 1 / 1 / 1 | 1 / 1 / 1 / 1 / 1 |
| SULT1C3 | chr2 | 108872115 | A>T | N163Y | 1 / 1 / 1 / 1 / 1 | 1 / 1 / 1 / 1 / 1 |
| TCEB3B | chr18 | 44559499 | A>T | C713S | 1 / 1 / 1 / 1 / 1 | 1 / 1 / 1 / 1 / 1 |
| TSPAN19 | chr12 | 85423699 | A>C | I9S | 0 / 1 / 0 / 1 / 0 | **1** / 1 / 0 / 1 / **1** |
| TTLL5 | chr14 | 76174022 | A>G | I238V | 1 / 1 / 1 / 1 / 1 | 1 / 1 / 1 / 1 / 1 |
| TTN | chr2 | 179582451 | T>A | N8067Y | 1 / 1 / 1 / 1 / 1 | 1 / 1 / 1 / 1 / 1 |
| UBQLN1 | chr9 | 86276850 | T>A | Q541L | 1 / 1 / 1 / 1 / 1 | 1 / 1 / 1 / 1 / 1 |
| UBTFL1 | chr11 | 89819328 | G>A | G71S | 1 / 1 / 1 / 1 / 1 | NA |
| UGP2 | chr2 | 64109627 | A>G | R84G | 0 / 0 / 0 / 1 / 0 | **1** / 0 / 0 / 1 / 0 |
| UNC13C | chr15 | 54306741 | T>A | S547R | 1 / 1 / 1 / 1 / 1 | NA |
| USP17L19 | chr4 | 9256230 | G>C | S376T | 0 / 0 / 0 / 0 / 1 | 0 / 0 / 0 / 0 / **0** |
| ZNF343 | chr20 | 2464537 | T>A | E398V | 1 / 1 / 1 / 1 / 1 | 1 / 1 / 1 / 1 / 1 |
| ZNF71 | chr19 | 57133437 | A>T | Q261L | 1 / 1 / 1 / 1 / 1 | 1 / 1 / 1 / 1 / 1 |

**Patient2**

| **Gene** | **Chr** | **Position** | **Nucleot-ide Variant** | **Amino**  **Acid**  **Change** | **Regions found**  **Mutated by NGS (P1T1/P1T2/P1T3**  **/P1T4/P1T5)** | **Regions found**  **Mutated by SS (P1T1/P1T2/P1T3**  **/P1T4/P1T5)** |
| --- | --- | --- | --- | --- | --- | --- |
| AHNAK | chr11 | 62294432 | T>C | D2486G | 1 / 1 / 1 / 1 / 1 | 1 / 1 / 1 / 1 / 1 |
| ANKDD1B | chr5 | 74921676 | G>A | D166N | 0 / 1 / 1 / 0 / 0 | 0 / 1 / 1 / 0 / 0 |
| ATP2A3 | chr17 | 3854643 | T>A | Y122F | 1 / 1 / 1 / 1 / 1 | 1 / 1 / 1 / 1 / 1 |
| BCAN | chr1 | 156622108 | G>C | E456Q | 1 / 1 / 1 / 1 / 1 | 1 / 1 / 1 / 1 / 1 |
| C11orf54 | chr11 | 93493013 | T>A | S255T | 0 / 0 / 0 / 1 / 0 | 0 / 0 / 0 / 1 / 0 |
| C22orf24 | chr22 | 32334219 | G>T | A12D | 0 / 0 / 0 / 1 / 0 | NA |
| C5orf15 | chr5 | 133295452 | G>C | N133K | 0 / 0 / 0 / 1 / 0 | 0 / 0 / 0 / 1 / 0 |
| C9orf171 | chr9 | 135285774 | C>T | A39V | 1 / 1 / 1 / 1 / 1 | 1 / 1 / 1 / 1 / 1 |
| CALN1 | chr7 | 71252787 | G>T | N253K | 1 / 0 / 0 / 1 / 1 | 1 / 0 / 0 / 1 / **0** |
| CASR | chr3 | 121980540 | C>T | R220W | 1 / 0 / 0 / 1 / 1 | 1 / 0 / 0 / 1 / 1 |
| CCDC171 | chr9 | 15744735 | A>T | L838F | 1 / 1 / 1 / 1 / 0 | NA |
| CCDC185 | chr1 | 223568114 | C>G | R433G | 0 / 0 / 1 / 0 / 0 | 0 / **1** / 1 / 0 / 0 |
| CCNJL | chr5 | 159680758 | G>A | A312V | 0 / 1 / 1 / 1 / 1 | NA |
| CDRT1 | chr17 | 15501890 | C>G | C504S | 0 / 1 / 0 / 0 / 0 | NA |
| CHD7 | chr8 | 61757898 | G>A | A1714T | 1 / 0 / 0 / 1 / 1 | 1 / 0 / 0 / 1 / **0** |
| CNKSR3 | chr6 | 154727609 | G>A | T516M | 0 / 0 / 0 / 1 / 0 | 0 / 0 / 0 / 1 / 0 |
| DCAF16 | chr4 | 17805377 | T>A | S130C | 1 / 1 / 1 / 1 / 1 | 1 / 1 / 1 / 1 / 1 |
| DDX4 | chr5 | 55062785 | A>T | N10Y | 1 / 1 / 1 / 1 / 0 | NA |
| DSP | chr6 | 7559514 | C>G | R160G | 1 / 1 / 0 / 1 / 1 | 1 / 1 / 0 / 1 / 1 |
| DTNA | chr18 | 32374201 | G>T | A117S | 0 / 1 / 0 / 0 / 0 | 0 / 1 / 0 / 0 / 0 |
| FLCN | chr17 | 17124839 | C>T | V295M | 1 / 1 / 1 / 1 / 1 | 1 / 1 / 1 / 1 / 1 |
| FLOT1 | chr6 | 30709461 | G>A | T47I | 0 / 0 / 0 / 0 / 1 | 0 / 0 / 0 / 0 / 1 |
| FRG2 | chr4 | 190946748 | T>G | T270P | 0 / 0 / 0 / 1 / 0 | NA |
| FSD1 | chr19 | 4323165 | G>T | A408S | 1 / 1 / 1 / 1 / 1 | 1 / 1 / 1 / 1 / 1 |
| GALNT7 | chr4 | 174219279 | G>T | V327L | 1 / 0 / 0 / 0 / 0 | NA |
| HIVEP3 | chr1 | 41978734 | G>T | S2053Y | 0 / 1 / 1 / 0 / 0 | 0 / 1 / 1 / 0 / 0 |
| HLA-B | chr6 | 31324184 | C>G | V127L | 1 / 0 / 0 / 0 / 0 | NA |
| HYAL3 | chr3 | 50332166 | G>C | R290G | 0 / 0 / 1 / 0 / 0 | 0 / 0 / 1 / 0 / 0 |
| IL4R | chr16 | 27367188 | A>C | I229L | 0 / 1 / 1 / 0 / 0 | 0 / 1 / 1 / 0 / 0 |
| INPP5E | chr9 | 139327525 | C>A | V388L | 0 / 1 / 0 / 0 / 0 | 0 / 1 / 0 / 0 / 0 |
| IQGAP2 | chr5 | 75896740 | A>G | N392S | 1 / 1 / 1 / 1 / 1 | 1 / 1 / 1 / 1 / 1 |
| ITGA9 | chr3 | 37785450 | G>C | E786D | 1 / 1 / 0 / 1 / 1 | 1 / 1 / 0 / 1 / 1 |
| KCNC3 | chr19 | 50824009 | C>T | A671T | 0 / 0 / 0 / 1 / 0 | 0 / 0 / 0 / 1 / 0 |
| KIAA1429 | chr8 | 95508686 | C>A | G1418V | 0 / 1 / 1 / 0 / 0 | 0 / 1 / 1 / 0 / 0 |
| KLK3 | chr19 | 51361754 | T>C | V178A | 1 / 1 / 1 / 1 / 1 | 1 / 1 / 1 / 1 / 1 |
| KMT2C | chr7 | 151919690 | C>T | S1134N | 0 / 0 / 0 / 0 / 1 | NA |
| METAP2 | chr12 | 95907526 | A>C | Y428S | 1 / 1 / 1 / 1 / 1 | 1 / 1 / 1 / 1 / 1 |
| MGAT4A | chr2 | 99242249 | A>T | N506K | 1 / 1 / 1 / 1 / 1 | 1 / 1 / 1 / 1 / 1 |
| MPRIP | chr17 | 16981340 | T>G | F73V | 0 / 1 / 0 / 0 / 0 | 0 / 1 / 0 / 0 / 0 |
| MRPS35 | chr12 | 27908294 | T>C | S295P | 1 / 0 / 0 / 0 / 0 | 1 / 0 / 0 / 0 / 0 |
| MUC17 | chr7 | 100679819 | A>C | S1708R | 1 / 1 / 1 / 1 / 1 | 1 / 1 / 1 / 1 / 1 |
| MXRA5 | chrX | 3229003 | C>T | G2414D | 1 / 0 / 0 / 0 / 0 | 1 / 0 / 0 / 0 / 0 |
| MYH15 | chr3 | 108156484 | T>G | K1066N | 1 / 1 / 0 / 1 / 1 | 1 / 1 / 0 / 1 / 1 |
| MYO1B | chr2 | 192250641 | A>T | E462V | 1 / 1 / 1 / 1 / 1 | 1 / 1 / 1 / 1 / 1 |
| NACAD | chr7 | 45123338 | G>A | S814L | 0 / 1 / 0 / 0 / 0 | **1** / 1 / 0 / **1** / 0 |
| ODF2 | chr9 | 131243910 | G>A | E280K | 1 / 1 / 0 / 1 / 1 | NA |
| OPRM1 | chr6 | 154414432 | T>A | W398R | 1 / 1 / 1 / 1 / 1 | 1 / 1 / 1 / 1 / 1 |
| OR5K4 | chr3 | 98072961 | G>T | E88D | 1 / 1 / 1 / 1 / 1 | 1 / 1 / 1 / 1 / 1 |
| PANX2 | chr22 | 50615682 | C>A | Q181K | 0 / 1 / 0 / 0 / 0 | 0 / 1 / 0 / 0 / 0 |
| PDGFRA | chr4 | 55154975 | C>G | P895R | 1 / 1 / 1 / 1 / 1 | NA |
| PHYHIP | chr8 | 22085800 | C>A | W24L | 1 / 0 / 0 / 0 / 0 | NA |
| PIK3C2A | chr11 | 17135997 | T>A | S1078C | 0 / 1 / 0 / 0 / 0 | 0 / 1 / 0 / 0 / 0 |
| PIM1 | chr6 | 37139040 | A>G | Q127R | 1 / 1 / 1 / 1 / 1 | 1 / 1 / 1 / 1 / 1 |
| PKD2L1 | chr10 | 102050257 | A>T | I629N | 0 / 0 / 0 / 1 / 0 | 0 / 0 / 0 / 1 / 0 |
| PLCL2 | chr3 | 17052046 | A>G | D277G | 0 / 1 / 1 / 0 / 0 | 0 / 1 / 1 / 0 / 0 |
| POTEJ | chr2 | 131414563 | G>A | D744N | 0 / 0 / 1 / 0 / 0 | NA |
| PRAMEF20 | chr1 | 13744674 | G>A | R122H | 0 / 1 / 0 / 0 / 0 | NA |
| PXDNL | chr8 | 52284498 | T>G | Q1279P | 0 / 0 / 0 / 1 / 0 | 0 / 0 / 0 / 1 / 0 |
| PYGB | chr20 | 25262697 | G>C | E478Q | 1 / 1 / 1 / 1 / 1 | 1 / 1 / 1 / 1 / 1 |
| RGMB | chr5 | 98129156 | C>A | P379H | 1 / 1 / 1 / 1 / 1 | 1 / 1 / 1 / 1 / 1 |
| RNASE2 | chr14 | 21424145 | T>G | L72R | 1 / 1 /0 / 1 / 1 | 1 / 1 / 0 / 1 / 1 |
| RP1 | chr8 | 55542796 | C>G | S2118R | 1 / 1 / 1 / 1 / 1 | 1 / 1 / 1 / 1 / 1 |
| S100A5 | chr1 | 153512567 | A>T | L34H | 0 / 1 / 1 / 1 / 1 | **1** / 1 / 1 / 1 / 1 |
| SEZ6L | chr22 | 26565679 | C>G | S15W | 0 / 1 / 1 / 0 / 1 | NA |
| SH3RF3 | chr2 | 109964238 | G>T | D228Y | 0 / 0 / 0 / 1 / 0 | 0 / 0 / 0 / 1 / 0 |
| SLC26A9 | chr1 | 205898405 | A>T | L266Q | 1 / 1 / 1 / 1 / 1 | 1 / 1 / 1 / 1 / 1 |
| SLFN11 | chr17 | 33690477 | T>A | E117V | 1 / 1 / 1 / 1 / 1 | 1 / 1 / 1 / 1 / 1 |
| SORCS3 | chr10 | 106974349 | C>A | T842N | 1 / 1 / 1 / 1 / 0 | 1 / 1 / 1 / 1 / **1** |
| SOX11 | chr2 | 5832906 | C>A | A18E | 1 / 1 / 1 / 1 / 1 | 1 / 1 / 1 / 1 / 1 |
| SP9 | chr2 | 175201871 | T>A | L353Q | 1 / 1 / 1 / 1 / 0 | NA |
| SSPO | chr7 | 149529921 | G>A | R5112Q | 0 / 1 / 1 / 0 / 0 | 0 / 1 / 1 / 0 / 0 |
| SYCP1 | chr1 | 115401310 | G>A | R145Q | 0 / 1 / 0 / 1 / 0 | 0 / 1 / 0 / **0** / 0 |
| TBC1D8B | chrX | 106097461 | A>C | S763R | 1 / 1 / 0 / 1 / 1 | 1 / 1 / 0 / 1 / 1 |
| TENM4 | chr11 | 78412845 | C>T | D1605N | 1 / 1 / 1 / 1 / 1 | 1 / 1 / 1 / 1 / 1 |
| TJP2 | chr9 | 71855002 | T>G | S835R | 1 / 1 / 0 / 1 / 1 | 1 / 1 / 0 / 1 / 1 |
| TMEM59L | chr19 | 18723922 | G>T | G52C | 0 / 0 / 0 / 0 / 1 | NA |
| TNFRSF21 | chr6 | 47220997 | T>A | T502S | 1 / 1 / 1 / 1 / 1 | NA |
| TPM2 | chr9 | 35685279 | C>T | E184K | 0 / 1 / 0 / 0 / 0 | 0 / 1 / 0 / 0 / 0 |
| TRAPPC9 | chr8 | 141301147 | T>A | E698V | 0 / 1 / 1 / 0 / 0 | 0 / 1 / 1 / 0 / 0 |
| TRPV3 | chr17 | 3417907 | G>A | P753L | 0 / 1 / 1 / 0 / 0 | 0 / 1 / 1 / 0 / 0 |
| TSEN2 | chr3 | 12544912 | A>T | N154Y | 1 / 1 / 0 / 1 / 1 | NA |
| TTC21A | chr3 | 39162668 | G>C | A320P | 0 / 0 / 0 / 1 / 0 | 0 / 0 / 0 / 1 / 0 |
| USH2A | chr1 | 216144050 | G>A | R2292C | 0 / 1 / 1 / 0 / 0 | 0 / 1 / 1 / 0 / 0 |
| VSTM1 | chr19 | 54544225 | A>T | L234Q | 1 / 1 / 1 / 1 / 1 | 1 / 1 / 1 / 1 / 1 |
| WASH1 | chr9 | 15978 | C>T | G376S | 1 / 0 / 0 / 0 / 0 | NA |
| WDR33 | chr2 | 128522085 | G>C | Q315E | 0 / 1 / 0 / 1 / 0 | NA |
| WRB | chr21 | 40762716 | G>T | M65I | 0 / 0 / 0 / 1 / 0 | 0 / 0 / 0 / 1 / 0 |
| WSCD1 | chr17 | 5984358 | C>T | P127L | 1 / 1 / 1 / 1 / 1 | 1 / 1 / 1 / 1 / 1 |
| ZDHHC8 | chr22 | 20127345 | G>C | V163L | 0 / 1 / 0 / 0 / 0 | 0 / 1 / 0 / 0 / 0 |
| ZNF814 | chr19 | 58385748 | G>A | A337V | 0 / 0 / 0 / 0 / 1 | 0 / 0 / 0 / 0 / **0** |
| ZNF831 | chr20 | 57768076 | T>A | S668T | 0 / 0 / 0 / 0 / 1 | 0 / 0 / 0 / 0 / 1 |

**Patient3**

| **Gene** | **Chr** | **Position** | **Nucleot-ide**  **Variant** | **Amino Acid Change** | **Regions found**  **Mutated by NGS (P1T1/P1T2/P1T3**  **/P1T4/P1T5)** | **Regions found**  **Mutated by SS (P1T1/P1T2/P1T3**  **/P1T4/P1T5)** |
| --- | --- | --- | --- | --- | --- | --- |
| ADAM23 | chr2 | 207425880 | A>G | S400G | 1 / 1 / 1 / 1 / 1 | 1 / 1 / 1 / 1 / 1 |
| AGAP9 | chr10 | 47213411 | C>A | G35V | 0 / 0 / 1 / 0 / 0 | NA |
| AMER3 | chr2 | 131520475 | A>T | Q277L | 1 / 1 / 1 / 1 / 1 | 1 / 1 / 1 / 1 / 1 |
| ANKFN1 | chr17 | 54535320 | G>A | A516T | 0 / 1 / 0 / 1 / 1 | 0 / 1 / 0 / 1 / 1 |
| ANKRD20A4 | chr9 | 69416170 | C>G | Q373E | 1 / 0 / 0 / 0 / 1 | NA |
| A09 | chr11 | 430142 | G>A | P238S | 1 / 1 / 1 / 1 / 1 | NA |
| AP3S1 | chr5 | 115205737 | A>T | K62I | 1 / 0 / 1 / 1 / 0 | 1 / **1** / 1 / 1 / 0 |
| APH1B | chr15 | 63594627 | A>T | I147F | 1 / 1 / 1 / 1 / 1 | 1 / 1 / 1 / 1 / 1 |
| ARMCX4 | chrX | 100749038 | C>T | A1821V | 0 / 0 / 1 / 0 / 0 | NA |
| BABAM1 | chr19 | 17379742 | G>A | A43T | 1 / 1 / 1 / 1 / 1 | 1 / 1 / 1 / 1 / 1 |
| BDP1 | chr5 | 70805315 | A>G | N799S | 1 / 0 / 1 / 1 / 1 | 1 / 1 / 1 / 1 / 1 |
| BRINP3 | chr1 | 190067449 | T>A | Q667L | 1 / 1 / 1 / 1 / 1 | 1 / 1 / 1 / 1 / 1 |
| CES1 | chr16 | 55857537 | C>T | G154E | 1 / 0 / 0 / 0 / 0 | 1 / 0 / **1** / 0 / **1** |
| CFI | chr4 | 110681527 | C>A | .G261V | 1 / 1 / 1 / 1 / 1 | 1 / 1 / 1 / 1 / 1 |
| CHGB | chr20 | 5904069 | C>T | R427C | 1 / 1 / 1 / 1 / 1 | 1 / 1 / 1 / 1 / 1 |
| CLGN | chr4 | 141317062 | C>A | G354W | 1 / 1 / 1 / 1 / 1 | 1 / 1 / 1 / 1 / 1 |
| COL4A6 | chrX | 107418361 | G>T | S972Y | 1 / 1 / 1 / 1 / 1 | NA |
| CROCC | chr1 | 17280781 | A>T | S1084C | 1 / 1 / 1 / 1 / 1 | 1 / 1 / 1 / 1 / 1 |
| CWC27 | chr5 | 64097145 | C>G | P256A | 1 / 1 / 1 / 1 / 1 | 1 / 1 / 1 / 1 / 1 |
| DENND5A | chr11 | 9202505 | G>T | P422T | 1 / 1 / 1 / 1 / 1 | 1 / 1 / 1 / 1 / 1 |
| EHBP1 | chr2 | 63175744 | A>G | E623G | 1 / 1 / 1 / 1 / 1 | 1 / 1 / 1 / 1 / 1 |
| FOXE3 | chr1 | 47882193 | G>A | R69H | 0 / 1 / 1 / 1 / 1 | NA |
| FOXI1 | chr5 | 169533233 | T>A | V91E | 0 / 1 / 1 / 1 / 1 | **1** / 1 / 1 / 1 / 1 |
| GAGE12E | chrX | 49330181 | A>G | M97V | 1 / 0 / 0 / 0 / 0 | **0** / 0 / 0 / 0 / 0 |
| GCN1L1 | chr12 | 120608012 | A>T | L494M | 1 / 1 / 1 / 1 / 1 | 1 / 1 / 1 / 1 / 1 |
| GHDC | chr17 | 40343138 | T>A | K327M | 1 / 1 / 1 / 1 / 1 | 1 / 1 / 1 / 1 / 1 |
| GOLGA6L1 | chr15 | 22743051 | G>A | R479K | 0 / 0 / 1 / 0 / 0 | NA |
| GRM6 | chr5 | 178421737 | T>A | E70V | 1 / 1 / 1 / 1 / 1 | NA |
| GSX1 | chr13 | 28367197 | T>A | Y124N | 1 / 1 / 1 / 1 / 1 | NA |
| HYDIN | chr16 | 70894024 | C>T | A4026T | 0 / 0 / 0 / 1 / 0 | **1** / **1** / **1** / 1 / **1** |
| KAZN | chr1 | 15441079 | T>A | L759Q | 1 / 1 / 1 / 1 / 1 | 1 / 1 / 1 / 1 / 1 |
| KLK6 | chr19 | 51470485 | A>T | L46H | 1 / 1 / 1 / 1 / 1 | 1 / 1 / 1 / 1 / 1 |
| LRP1 | chr12 | 57598955 | G>A | R3753H | 1 / 1 / 1 / 1 / 1 | 1 / 1 / 1 / 1 / 1 |
| LRRC10 | chr12 | 70003915 | C>T | R235H | 1 / 1 / 1 / 1 / 1 | 1 / 1 / 1 / 1 / 1 |
| MAGEC1 | chrX | 140995256 | T>A | L689Q | 1 / 1 / 1 / 1 / 1 | NA |
| MAPK6 | chr15 | 52356597 | T>G | N522K | 0 / 0 / 0 / 1 / 0 | NA |
| MYBPH | chr1 | 203140298 | A>C | L275R | 0 / 1 / 0 / 1 / 1 | 0 / 1 / **1** / 1 / 1 |
| NACAD | chr7 | 45123338 | G>A | S814L | 1 / 1 / 0 / 1 / 0 | NA |
| NADK | chr1 | 1688652 | G>A | P121S | 1 / 1 / 1 / 1 / 1 | NA |
| NETO2 | chr16 | 47143609 | A>C | F223C | 1 / 1 / 1 / 1 / 1 | 1 / 1 / 1 / 1 / 1 |
| NFATC2IP | chr16 | 28967483 | A>T | K224M | 1 / 1 / 1 / 1 / 1 | 1 / 1 / 1 / 1 / 1 |
| OR5I1 | chr11 | 55703058 | A>T | D273E | 1 / 1 / 1 / 1 / 1 | 1 / 1 / 1 / 1 / 1 |
| PCSK5 | chr9 | 78686767 | G>A | G283R | 1 / 1 / 1 / 1 / 1 | 1 / 1 / 1 / 1 / 1 |
| PIK3C2G | chr12 | 18650572 | A>T | Q969L | 1 / 1 / 1 / 1 / 1 | 1 / 1 / 1 / 1 / 1 |
| PLEKHM2 | chr1 | 16053757 | A>C | E397A | 1 / 1 / 1 / 1 / 1 | 1 / 1 / 1 / 1 / 1 |
| PLSCR3 | chr17 | 7297132 | C>T | A11T | 1 / 0 / 0 / 0 / 0 | NA |
| PSD | chr10 | 104176192 | G>A | R202C | 0 / 1 / 0 / 1 / 1 | 0 / 1 / **1** / 1 / 1 |
| PTPRR | chr12 | 71078019 | A>T | F256Y | 1 / 1 / 1 / 1 / 1 | 1 / 1 / 1 / 1 / 1 |
| RAB8B | chr15 | 63541865 | A>T | R69S | 0 / 0 / 0 / 0 / 1 | **1** / 0 / 0 / 0 / 1 |
| RFTN1 | chr3 | 16399479 | C>T | G369D | 1 / 1 / 1 / 1 / 1 | 1 / 1 / 1 / 1 / 1 |
| RIPK2 | chr8 | 90802619 | A>C | N533T | 1 / 1 / 1 / 1 / 1 | 1 / 1 / 1 / 1 / 1 |
| SETD2 | chr3 | 47127746 | A>C | L1779W | 1 / 1 / 1 / 1 / 1 | 1 / 1 / 1 / 1 / 1 |
| SLC6A5 | chr11 | 20657892 | C>G | T555S | 1 / 1 / 1 / 1 / 1 | 1 / 1 / 1 / 1 / 1 |
| SLC9A2 | chr2 | 103318913 | T>A | D599E | 1 / 1 / 1 / 1 / 1 | 1 / 1 / 1 / 1 / 1 |
| SLC9A9 | chr3 | 143212515 | T>A | Q432L | 1 / 1 / 1 / 1 / 1 | 1 / 1 / 1 / 1 / 1 |
| SLCO1B7 | chr12 | 21207396 | A>T | N456I | 0 / 0 / 0 / 1 / 0 | 0 / 0 / 0 / 1 / 0 |
| SLK | chr10 | 105761224 | C>A | S296Y | 1 / 1 / 1 / 1 / 1 | 1 / 1 / 1 / 1 / 1 |
| SPTBN2 | chr11 | 66468046 | T>A | Q1175L | 1 / 1 / 1 / 1 / 1 | 1 / 1 / 1 / 1 / 1 |
| ST18 | chr8 | 53028864 | T>A | S992C | 1 / 1 / 1 / 1 / 1 | 1 / 1 / 1 / 1 / 1 |
| TRIM65 | chr17 | 73888548 | C>A | G182C | 1 / 1 / 1 / 1 / 1 | 1 / 1 / 1 / 1 / 1 |
| TTLL5 | chr14 | 76174022 | A>G | I238V | 1 / 1 / 1 / 1 / 1 | 1 / 1 / 1 / 1 / 1 |
| VIM | chr10 | 17271477 | G>T | G19V | 1 / 0 / 0 / 0 / 0 | NA |
| WDR81 | chr17 | 1628566 | G>A | V105M | 1 / 1 / 1 / 1 / 1 | 1 / 1 / 1 / 1 / 1 |
| ZCRB1 | chr12 | 42706961 | T>A | S188C | 1 / 1 / 1 / 1 / 1 | 1 / 1 / 1 / 1 / 1 |
| ZNF304 | chr19 | 57868159 | A>T | T355S | 1 / 1 / 1 / 1 / 1 | 1 / 1 / 1 / 1 / 1 |
| ZNF746 | chr7 | 149174757 | C>A | V204L | 1 / 1 / 1 / 1 / 1 | 1 / 1 / 1 / 1 / 1 |
| ZSWIM3 | chr20 | 44505628 | C>T | S144F | 1 / 1 / 1 / 1 / 1 | 1 / 1 / 1 / 1 / 1 |

**Patient4**

| **Gene** | **Chr** | **Position** | **Nucleot-ide**  **Variant** | **Amino Acid Change** | **Regions found**  **Mutated by NGS (P1T1/P1T2/P1T3**  **/P1T4/P1T5)** | **Regions found**  **Mutated by SS (P1T1/P1T2/P1T3**  **/P1T4/P1T5)** |
| --- | --- | --- | --- | --- | --- | --- |
| ABCB5 | chr7 | 20698171 | G>A | G527R | 1 / 0 / 1 / 1 / 0 | 1 / 0 / 1 / 1 / 0 |
| ACR | chr22 | 51183193 | A>G | Y275C | 0 / 0 / 0 / 1 / 0 | NA |
| ACTN2 | chr1 | 236917325 | C>T | R640C | 0 / 1 / 0 / 0 / 1 | 0 / 1 / 0 / 0 / 1 |
| ADAMTS20 | chr12 | 43846157 | A>T | Y667N | 0 / 1 / 0 / 0 / 1 | 0 / 1 / 0 / 0 / 1 |
| AKAP9 | chr7 | 91630185 | C>G | N318K | 1 / 1 / 1 / 1 / 1 | 1 / 1 / 1 / 1 / 1 |
| ALG5 | chr13 | 37546115 | T>A | Q177L | 1 / 0 / 1 / 1 / 0 | 1 / 0 / 1 / 1 / 0 |
| ANKRD30B | chr18 | 14757865 | G>A | M223I | 0 / 0 / 1 / 0 / 0 | 0 / 0 / 1 / 0 / 0 |
| ARMC8 | chr3 | 137960814 | G>A | D329N | 1 / 1 / 1 / 1 / 1 | 1 / 1 / 1 / 1 / 1 |
| BMP5 | chr6 | 55739340 | C>A | E108D | 1 / 1 / 1 / 1 / 1 | 1 / 1 / 1 / 1 / 1 |
| C2orf78 | chr2 | 74042437 | G>C | D363H | 1 / 1 / 1 / 1 / 1 | 1 / 1 / 1 / 1 / 1 |
| CACNB2 | chr10 | 18690905 | A>G | E35G | 1 / 0 / 1 / 0 / 0 | 1 / 0 / 1 / 0 / 0 |
| CCDC18 | chr1 | 93691953 | T>G | L747V | 1 / 0 / 0 / 1 / 0 | NA |
| CDK5RAP2 | chr9 | 123169323 | T>A | I1414F | 1 / 1 / 1 / 1 / 1 | 1 / 1 / 1 / 1 / 1 |
| CENPF | chr1 | 214794224 | A>G | N267S | 1 / 0 / 1 / 0 / 0 | 1 / 0 / 1 / 0 / 0 |
| CFTR | chr7 | 117188736 | C>A | N417K | 1 / 0 / 1 / 0 / 0 | NA |
| CHAT | chr10 | 50835685 | G>A | R240H | 1 / 0 / 1 / 0 / 0 | 1 / 0 / 1 / 0 / 0 |
| CIC | chr19 | 42798866 | G>T | D1480Y | 1 / 1 / 1 / 1 / 1 | NA |
| COL11A2 | chr6 | 33140374 | T>A | H944L | 1 / 1 / 1 / 1 / 1 | 1 / 1 / 1 / 1 / 1 |
| COL4A1 | chr13 | 110833715 | T>C | D706G | 0 / 0 / 1 / 0 / 0 | 0 / 0 / 1 / 0 / 0 |
| CRTC2 | chr1 | 153927455 | T>A | H89L | 0 / 1 / 0 / 0 / 1 | 0 / 1 / 0 / 0 / 1 |
| CYP26B1 | chr2 | 72359592 | C>T | G435S | 1 / 1 / 1 / 1 / 1 | 1 / 1 / 1 / 1 / 1 |
| DIAPH1 | chr5 | 140957894 | T>C | E345G | 0 / 1 / 0 / 0 / 1 | 0 / 1 / 0 / 0 / 1 |
| DYRK4 | chr12 | 4714085 | A>G | T263A | 0 / 0 / 0 / 1 / 0 | 0 / 0 / 0 / 1 / 0 |
| EPS15L1 | chr19 | 16472679 | C>A | D833Y | 0 / 1 / 1 / 0 / 1 | NA |
| FAM157B | chr9 | 141121505 | A>G | T347A | 0 / 1 / 1 / 1 / 0 | NA |
| FAM161B | chr14 | 74413287 | C>T | A89T | 0 / 1 / 0 / 0 / 1 | 0 / 1 / 0 / 0 / 1 |
| GFRA1 | chr10 | 117884749 | C>A | K246N | 1 / 1 / 1 / 1 / 1 | 1 / 1 / 1 / 1 / 1 |
| GLI2 | chr2 | 121726466 | G>A | G274R | 1 / 1 / 1 / 1 / 1 | 1 / 1 / 1 / 1 / 1 |
| GPR148 | chr2 | 131486744 | C>A | P7H | 0 / 1 / 0 / 0 / 1 | 0 / 1 / 0 / 0 / 1 |
| HMGA2 | chr12 | 66232333 | G>T | G78V | 0 / 1 / 0 / 0 / 1 | 0 / 1 / 0 / 0 / 1 |
| HSP90AA1 | chr14 | 102552603 | T>A | Y38F | 0 / 1 / 0 / 0 / 1 | 0 / 1 / 0 / 0 / 1 |
| HSPA8 | chr11 | 122930403 | C>A | A300S | 1 / 1 / 1 / 1 / 1 | 1 / 1 / 1 / 1 / 1 |
| INSR | chr19 | 7152891 | C>A | D693Y | 1 / 0 / 0 / 1 / 0 | 1 / 0 / **1** / 1 / 0 |
| IQCH | chr15 | 67665808 | C>G | S297C | 0 / 0 / 0 / 1 / 0 | 0 / 0 / 0 / 1 / 0 |
| IRX1 | chr5 | 3599351 | G>A | E79K | 0 / 0 / 1 / 0 / 0 | 0 / 0 / 1 / 0 / 0 |
| IRX1 | chr5 | 3599504 | G>A | E148K | 0 / 0 / 1 / 0 / 0 | 0 / 0 / 1 / 0 / 0 |
| IRX1 | chr5 | 3599666 | G>A | D202N | 0 / 0 / 1 / 0 / 0 | 0 / 0 / 1 / 0 / 0 |
| IRX1 | chr5 | 3599720 | G>T | D220Y | 0 / 0 / 1 / 0 / 0 | 0 / 0 / 1 / 0 / 0 |
| KCNH8 | chr3 | 19432021 | T>G | V287G | 1 / 1 / 1 / 1 / 1 | 1 / 1 / 1 / 1 / 1 |
| KCNU1 | chr8 | 36644887 | T>A | F87I | 1 / 1 / 1 / 1 / 1 | 1 / 1 / 1 / 1 / 1 |
| LPL | chr8 | 19813391 | T>A | I272N | 1 / 1 / 1 / 1 / 1 | 1 / 1 / 1 / 1 / 1 |
| LZTS2 | chr10 | 102766419 | C>T | R502W | 0 / 0 / 0 / 0 / 1 | 0 / 1 / 0 / 0 / 1 |
| MED13L | chr12 | 116460390 | C>A | A166S | 1 / 0 / 1 / 1 / 1 | 1 / **1** / 1 / 1 / 1 |
| MLX | chr17 | 40719335 | G>A | A65T | 1 / 1 / 1 / 1 / 1 | 1 / 1 / 1 / 1 / 1 |
| MMEL1 | chr1 | 2524101 | G>T | A687D | 0 / 0 / 1 / 0 / 0 | NA |
| MTPAP | chr10 | 30615371 | T>G | D325A | 1 / 1 / 1 / 1 / 1 | 1 / 1 / 1 / 1 / 1 |
| MUCL1 | chr12 | 55250668 | A>G | D72G | 0 / 0 / 0 / 0 / 1 | NA |
| NFYB | chr12 | 104514253 | G>T | N172K | 1 / 0 / 0 / 0 / 0 | 1 / 0 / 0 / 0 / 0 |
| NGFRAP1 | chrX | 102632641 | G>A | M154I | 1 / 1 / 1 / 1 / 1 | 1 / 1 / 1 / 1 / 1 |
| 0LC1 | chr10 | 103918959 | C>T | A207V | 0 / 1 / 0 / 0 / 1 | 0 / 1 / 0 / 0 / 1 |
| OR1N2 | chr9 | 125316254 | A>G | Y269C | 0 / 0 / 0 / 0 / 1 | 0 / **1** / 0 / 0 / 1 |
| PDE1A | chr1 | 183095801 | T>A | S175C | 0 / 1 / 0 / 0 / 1 | 0 / 1 / 0 / 0 / 1 |
| PDZD2 | chr5 | 32087855 | T>C | L1434P | 1 / 1 / 1 / 1 / 1 | 1 / 1 / 1 / 1 / 1 |
| PIGN | chr18 | 59825030 | A>G | M78T | 1 / 1 / 1 / 1 / 1 | 1 / 1 / 1 / 1 / 1 |
| PLEKHA5 | chr12 | 19427814 | T>C | Y398H | 1 / 1 / 1 / 1 / 1 | 1 / 1 / 1 / 1 / 1 |
| PPWD1 | chr5 | 64859258 | G>A | E41K | 1 / 1 / 1 / 1 / 1 | 1 / 1 / 1 / 1 / 1 |
| PRLHR | chr10 | 120354693 | C>A | V22F | 1 / 1 / 1 / 1 / 1 | 1 / 1 / 1 / 1 / 1 |
| PTPRB | chr12 | 71029684 | T>C | N73S | 1 / 1 / 1 / 1 / 1 | 1 / 1 / 1 / 1 / 1 |
| SMURF2 | chr17 | 62574632 | A>C | W279G | 1 / 1 / 1 / 1 / 1 | 1 / 1 / 1 / 1 / 1 |
| SP1 | chr12 | 53777314 | G>T | G528V | 1 / 1 / 1 / 1 / 1 | 1 / 1 / 1 / 1 / 1 |
| SPRR2A | chr1 | 153029121 | T>C | K31E | 0 / 1 / 0 / 0 / 1 | 0 / 1 / 0 / 0 / 1 |
| SSTR4 | chr20 | 23016986 | A>G | D289G | 0 / 0 / 0 / 0 / 1 | 0 / 0 / 0 / 0 / 1 |
| TMEM31 | chrX | 102968548 | A>T | Q43H | 1 / 1 / 1 / 1 / 1 | 1 / 1 / 1 / 1 / 1 |
| TPRN | chr9 | 140094097 | C>A | G356V | 0 / 0 / 0 / 0 / 1 | NA |
| TRIM49D1 | chr11 | 89650162 | C>T | M144I | 0 / 0 / 0 / 1 / 0 | NA |
| TRIM49D2 | chr11 | 89660646 | G>A | M144I | 0 / 0 / 1 / 0 / 0 | NA |
| ZNF14 | chr19 | 19822263 | G>T | H609Q | 0 / 0 / 1 / 0 / 0 | 0 / 0 / 1 / 0 / 0 |
| ZNF727 | chr7 | 63537704 | G>T | D93Y | 1 / 0 / 1 / 1 / 0 | NA |

**Patient5**

| **Gene** | **Chr** | **Position** | **Nucleot-ide**  **Variant** | **Amino Acid Change** | **Regions found**  **Mutated by NGS (P1T1/P1T2/P1T3**  **/P1T4/P1T5)** | **Regions found**  **Mutated by SS (P1T1/P1T2/P1T3**  **/P1T4/P1T5)** |
| --- | --- | --- | --- | --- | --- | --- |
| ABCB6 | chr2 | 220077985 | C>T | V595M | 0 / 0 / 0 / 0 / 1 | 0 / 0 / 0 / 0 / 1 |
| ABI3BP | chr3 | 100594375 | G>T | A272D | 1 / 1 / 1 / 1 / 1 | 1 / 1 / 1 / 1 / 1 |
| ACOX2 | chr3 | 58490989 | T>C | I670V | 1 / 1 / 1 / 1 / 1 | 1 / 1 / 1 / 1 / 1 |
| ADAD2 | chr16 | 84229555 | C>G | A396G | 1 / 1 / 1 / 1 / 1 | NA |
| AGAP9 | chr10 | 48215939 | G>T | G35V | 0 / 0 / 0 / 0 / 1 | NA |
| AGRN | chr1 | 957753 | G>T | W125L | 1 / 1 / 1 / 1 / 1 | 1 / 1 / 1 / 1 / 1 |
| ANKS1A | chr6 | 34949749 | G>T | D240Y | 1 / 1 / 1 / 1 / 1 | 1 / 1 / 1 / 1 / 1 |
| ANO6 | chr12 | 45803270 | A>T | I671F | 1 / 1 / 1 / 1 / 1 | 1 / **0** / 1 / 1 / 1 |
| ARMC9 | chr2 | 232141363 | C>G | T450R | 1 / 1 / 1 / 1 / 1 | 1 / 1 / **0** / 1 / 1 |
| ARPC1A | chr7 | 98942062 | C>G | P92A | 1 / 1 / 1 / 1 / 1 | 1 / 1 / 1 / 1 / 1 |
| ATXN3 | chr14 | 92537379 | T>C | T85A | 0 / 0 / 1 / 0 / 0 | NA |
| BID | chr22 | 18232894 | C>A | A43S | 1 / 1 / 1 / 1 / 1 | 1 / 1 / 1 / 1 / 1 |
| C3orf84 | chr3 | 49215925 | G>T | S63Y | 1 / 1 / 1 / 1 / 1 | 1 / 1 / 1 / 1 / 1 |
| CBWD6 | chr9 | 69238258 | C>T | D212N | 0 / 1 / 1 / 0 / 0 | NA |
| CCDC141 | chr2 | 179914601 | G>A | A23V | 1 / 1 / 1 / 1 / 1 | 1 / 1 / 1 / 1 / 1 |
| CCDC66 | chr3 | 56651532 | A>C | N746H | 1 / 1 / 1 / 1 / 1 | 1 / 1 / 1 / 1 / 1 |
| CCDC78 | chr16 | 775254 | G>T | H159N | 1 / 1 / 1 / 1 / 1 | NA |
| CEACAM5 | chr19 | 42212701 | G>T | R17S | 1 / 1 / 1 / 1 / 1 | 1 / 1 / 1 / 1 / 1 |
| CHD9 | chr16 | 53243446 | A>G | N502S | 1 / 1 / 1 / 1 / 1 | 1 / 1 / 1 / 1 / 1 |
| CHST5 | chr16 | 75563403 | G>A | R294C | 1 / 1 / 1 / 1 / 1 | **0** / 1 / 1 / 1 / 1 |
| CLIC3 | chr9 | 139889456 | G>T | R160S | 0 / 0 / 0 / 1 / 0 | NA |
| CNTNAP4 | chr16 | 76573743 | T>G | F1043L | 1 / 1 / 1 / 1 / 1 | 1 / 1 / 1 / 1 / 1 |
| CUL1 | chr7 | 148463681 | G>T | R273L | 1 / 1 / 1 / 1 / 1 | 1 / 1 / 1 / 1 / 1 |
| CYP2D6 | chr22 | 42526726 | A>G | M23T | 1 / 1 / 1 / 1 / 1 | NA |
| DCHS2 | chr4 | 155410754 | G>T | A585D | 1 / 1 / 1 / 1 / 1 | NA |
| DEAF1 | chr11 | 694849 | C>T | A67T | 0 / 0 / 1 / 0 / 0 | NA |
| DEFB126 | chr20 | 126221 | C>A | T75K | 1 / 1 / 1 / 1 / 1 | 1 / 1 / 1 / 1 / 1 |
| DMP1 | chr4 | 88583684 | C>G | Q236E | 1 / 1 / 1 / 1 / 1 | 1 / 1 / 1 / 1 / 1 |
| DNAJB3 | chr2 | 234652249 | C>A | G105V | 1 / 1 / 1 / 1 / 1 | 1 / 1 / 1 / 1 / 1 |
| DOK6 | chr18 | 67406274 | T>C | S225P | 1 / 1 / 1 / 1 / 1 | 1 / 1 / 1 / 1 / 1 |
| ERRFI1 | chr1 | 8073286 | T>C | Y458C | 1 / 1 / 1 / 1 / 1 | 1 / 1 / 1 / 1 / 1 |
| FAR2 | chr12 | 29450049 | C>A | A154D | 1 / 1 / 1 / 1 / 1 | 1 / 1 / 1 / 1 / 1 |
| FBLN2 | chr3 | 13655503 | G>T | G523V | 1 / 1 / 1 / 1 / 1 | 1 / 1 / **0** / 1 / 1 |
| FBXL19 | chr16 | 30941836 | G>T | R431L | 1 / 1 / 1 / 1 / 1 | 1 / 1 / 1 / 1 / 1 |
| FGFRL1 | chr4 | 1018810 | G>T | W397L | 1 / 1 / 1 / 1 / 1 | 1 / 1 / **0** / 1 / 1 |
| FMO3 | chr1 | 171083484 | G>T | A389S | 1 / 1 / 1 / 1 / 1 | 1 / 1 / **0** / 1 / 1 |
| GPATCH3 | chr1 | 27226845 | C>T | R30Q | 1 / 1 / 1 / 1 / 1 | 1 / 1 / 1 / 1 / 1 |
| GPN2 | chr1 | 27216551 | C>A | A13S | 1 / 1 / 1 / 1 / 1 | 1 / 1 / 1 / 1 / 1 |
| GPR155 | chr2 | 175318491 | T>G | E617A | 1 / 1 / 1 / 1 / 1 | NA |
| HBG1 | chr11 | 5270621 | G>A | H98Y | 1 / 1 / 1 / 1 / 1 | 1 / 1 / 1 / 1 / 1 |
| HEG1 | chr3 | 124738328 | C>A | A456S | 1 / 1 / 1 / 1 / 1 | 1 / 1 / 1 / 1 / 1 |
| HFM1 | chr1 | 91844015 | C>A | R394M | 1 / 1 / 1 / 1 / 1 | 1 / **0** / 1 / 1 / 1 |
| HOXB13 | chr17 | 46805438 | G>A | A173V | 1 / 1 / 0 / 1 / 1 | 1 / 1 / 0 / 1 / 1 |
| HP | chr16 | 72094646 | G>T | A301S | 1 / 1 / 1 / 1 / 1 | 1 / 1 / 1 / 1 / 1 |
| HSD11B1L | chr19 | 5687945 | G>T | R172L | 1 / 1 / 1 / 1 / 1 | NA |
| IQSEC2 | chrX | 53264048 | C>T | G1274R | 1 / 1 / 1 / 0 / 1 | NA |
| ITSN2 | chr2 | 24480899 | G>A | H889Y | 1 / 1 / 1 / 1 / 1 | 1 / 1 / **0** / 1 / 1 |
| KCNJ6 | chr21 | 39087429 | C>T | V11I | 1 / 1 / 1 / 1 / 1 | 1 / 1 / 1 / 1 / 1 |
| KIAA0232 | chr4 | 6860182 | C>G | S156C | 1 / 1 / 1 / 1 / 1 | 1 / 1 / 1 / 1 / 1 |
| KIAA0556 | chr16 | 27709757 | A>G | E350G | 1 / 1 / 1 / 1 / 1 | 1 / 1 / 1 / 1 / 1 |
| KIDINS220 | chr2 | 8910941 | T>A | Q957L | 1 / 1 / 1 / 1 / 1 | 1 / 1 / 1 / 1 / 1 |
| KLHL14 | chr18 | 30350194 | C>A | A121S | 1 / 1 / 1 / 1 / 1 | 1 / 1 / 1 / 1 / 1 |
| KRT10 | chr17 | 38975250 | A>G | Y513H | 1 / 0 / 0 / 0 / 1 | NA |
| L1CAM | chrX | 153132271 | C>T | R755H | 1 / 1 / 1 / 1 / 1 | 1 / 1 / 1 / 1 / 1 |
| LRIG2 | chr1 | 113657271 | T>A | M768K | 1 / 1 / 1 / 1 / 1 | NA |
| LRRK1 | chr15 | 101464901 | G>T | V22L | 1 / 1 / 1 / 1 / 1 | 1 / 1 / 1 / 1 / 1 |
| MAS1 | chr6 | 160328098 | G>T | W37C | 1 / 1 / 1 / 1 / 1 | 1 / 1 / 1 / 1 / 1 |
| MDC1 | chr6 | 30680554 | C>A | V389F | 1 / 1 / 1 / 1 / 1 | 1 / 1 / 1 / 1 / 1 |
| MGAT5B | chr17 | 74868914 | G>T | R28L | 1 / 1 / 1 / 1 / 1 | 1 / 1 / 1 / 1 / 1 |
| MIEF2 | chr17 | 18167276 | C>A | A188E | 1 / 1 / 1 / 1 / 1 | 1 / 1 / **0** / 1 / 1 |
| MRC2 | chr17 | 60741939 | G>T | G50V | 1 / 1 / 1 / 1 / 1 | 1 / 1 / 1 / 1 / 1 |
| MTHFR | chr1 | 11855385 | C>A | Q267H | 1 / 1 / 1 / 1 / 1 | 1 / 1 / 1 / 1 / 1 |
| MYCBP2 | chr13 | 77752022 | C>A | G1734V | 1 / 1 / 1 / 1 / 1 | 1 / 1 / 1 / **0** / 1 |
| N4BP3 | chr5 | 177548913 | C>A | R516S | 1 / 1 / 1 / 1 / 1 | 1 / 1 / 1 / 1 / 1 |
| NBAS | chr2 | 15514736 | C>A | L1233F | 1 / 1 / 1 / 1 / 1 | NA |
| NBPF10 | chr1 | 146465178 | A>T | K3688I | 0 / 1 / 0 / 0 / 0 | 0 / **0** / 0 / 0 / 0 |
| NLRP4 | chr19 | 56369085 | G>T | R109L | 1 / 1 / 1 / 1 / 1 | 1 / 1 / 1 / 1 / 1 |
| 0C2L | chr1 | 892580 | T>C | K85E | 1 / 1 / 1 / 1 / 1 | 1 / 1 / 1 / 1 / 1 |
| NPTXR | chr22 | 39222727 | A>T | D292E | 1 / 1 / 1 / 1 / 1 | 1 / 1 / 1 / 1 / 1 |
| OR51A2 | chr11 | 4976013 | A>G | W311R | 0 / 1 / 0 / 1 / 0 | **0 / 1 / 0 / 1 / 0** |
| OR5T3 | chr11 | 56019882 | G>T | L69F | 1 / 1 / 1 / 1 / 1 | 1 / 1 / 1 / 1 / 1 |
| OTOF | chr2 | 26688591 | C>A | R1583L | 1 / 1 / 1 / 1 / 1 | 1 / 1 / **0** / 1 / 1 |
| OTX2 | chr14 | 57268736 | A>C | F204C | 1 / 1 / 1 / 1 / 1 | 1 / 1 / 1 / 1 / 1 |
| PCMTD1 | chr8 | 52733041 | T>A | E139V | 1 / 1 / 1 / 1 / 1 | NA |
| PDZRN3 | chr3 | 73433784 | G>A | R302C | 1 / 1 / 1 / 1 / 1 | 1 / 1 / 1 / 1 / 1 |
| PKN1 | chr19 | 14552255 | G>T | D114Y | 1 / 1 / 1 / 1 / 1 | 1 / 1 / 1 / 1 / 1 |
| PRAMEF6 | chr1 | 13002068 | T>C | H94R | 0 / 0 / 0 / 0 / 1 | NA |
| PROX1 | chr1 | 214184915 | G>A | E629K | 1 / 1 / 1 / 1 / 1 | 1 / 1 / 1 / 1 / 1 |
| PXDNL | chr8 | 52325779 | G>A | S612F | 1 / 1 / 1 / 1 / 1 | 1 / 1 / 1 / 1 / 1 |
| RBM10 | chrX | 47045683 | G>A | G778E | 1 / 1 / 1 / 1 / 1 | NA |
| RHO | chr3 | 129251159 | A>G | N199S | 1 / 1 / 1 / 1 / 1 | 1 / 1 / 1 / 1 / 1 |
| RLTPR | chr16 | 67684013 | G>A | R715H | 0 / 0 / 0 / 1 / 0 | 0 / 0 / 0 / 1 / 0 |
| RYR1 | chr19 | 39055851 | G>T | A4288S | 1 / 0 / 0 / 0 / 0 | NA |
| SCN2A | chr2 | 166179706 | G>T | R571L | 1 / 1 / 1 / 1 / 1 | 1 / 1 / **0** / 1 / 1 |
| SEC16A | chr9 | 139341431 | T>A | N2245I | 0 / 0 / 1 / 0 / 0 | 0 / 0 / 1 / 0 / 0 |
| SEMA3A | chr7 | 83689847 | G>A | H161Y | 1 / 1 / 1 / 1 / 1 | 1 / 1 / 1 / 1 / 1 |
| SENP1 | chr12 | 48468289 | G>C | S253C | 1 / 1 / 1 / 1 / 1 | 1 / 1 / 1 / 1 / 1 |
| SERPINB10 | chr18 | 61582881 | C>A | A46D | 1 / 1 / 1 / 1 / 1 | 1 / 1 / 1 / 1 / 1 |
| SH3TC1 | chr4 | 8224570 | G>T | L372F | 1 / 1 / 1 / 1 / 1 | 1 / 1 / 1 / 1 / 1 |
| SIX4 | chr14 | 61190227 | G>T | A189E | 1 / 1 / 1 / 1 / 1 | 1 / 1 / 1 / 1 / 1 |
| SRD5A3 | chr4 | 56236224 | A>T | K308M | 1 / 0 / 1 / 1 / 1 | 1 / **1** / 1 / 1 / 1 |
| SREBF1 | chr17 | 17716110 | C>A | L1090F | 1 / 1 / 0 / 0 / 1 | NA |
| SYCP1 | chr1 | 115487502 | G>T | A685S | 1 / 1 / 1 / 1 / 1 | NA |
| TAAR5 | chr6 | 132909861 | T>C | Q322R | 1/ 1 / 1 / 1 / 1 | 1/ 1 / 1 / 1 / 1 |
| THBS2 | chr6 | 169632184 | T>A | Q681L | 1/ 1 / 1 / 1 / 1 | 1/ 1 / 1 / 1 / 1 |
| THSD1 | chr13 | 52952508 | C>A | A480S | 1 / 1 / 1 / 1 / 1 | 1 / 1 / 1 / 1 / 1 |
| TNFSF13 | chr17 | 7462511 | A>G | Q52R | 0 / 0 / 0 / 1 / 0 | NA |
| TNN | chr1 | 175049503 | C>A | T330N | 1 / 1 / 1 / 1 / 1 | 1 / 1 / **0** / 1 / 1 |
| TRAPPC3 | chr1 | 36602905 | C>A | A82S | 1 / 1 / 1 / 1 / 1 | 1 / 1 / 1 / 1 / 1 |
| TRIM49D1 | chr11 | 89646429 | G>T | L268I | 1 / 0 / 0 / 1 / 1 | NA |
| TRIM49D1 | chr11 | 89664379 | C>A | L268I | 0 / 0 / 1 / 1 / 0 | NA |
| UNC79 | chr14 | 94079363 | C>A | F1148L | 1 / 1 / 1 / 1 / 1 | 1 / 1 / 1 / 1 / 1 |
| VCAN | chr5 | 82785949 | T>A | S35T | 1 / 1 / 1 / 1 / 1 | 1 / 1 / **0** / 1 / 1 |
| VWA5B1 | chr1 | 20637132 | T>A | L13Q | 1 / 1 / 1 / 1 / 1 | 1 / 1 / 1 / 1 / 1 |
| WDR43 | chr2 | 29152515 | C>T | T459M | 1 / 1 / 1 / 1 / 1 | NA |
| ZNF827 | chr4 | 146813545 | C>A | K372N | 1 / 1 / 1 / 1 / 1 | 1 / 1 / 1 / 1 / 1 |

Chr: chromosome; NGS: Next-generation sequencing; SS: Sanger sequencing; 1 indicate mutation was detected, 0 indicate mutation was not detected; NA: indicate the mutation were not selected for Sanger sequencing; 1 that indicated in bold suggests mutation found by SS, but not NGS, 0 that indicated in bold suggests mutation found by NGS, but not SS.
