# Supplementary material for: Multi-omics study revealing the complexity and spatial heterogeneity of tumor-infiltrating lymphocytes in primary liver carcinoma
Source: Oncotarget. 2017 Mar 31;8(21):34844–57. doi: 10.18632/oncotarget.16758 (PMC5471016; doi:10.18632/oncotarget.16758)
Supplement: Supplementary file 3 [file oncotarget-08-34844-s003.docx]

**Supplementary Table S7: TCR sequencing primers**

| **Primer name** | **sequences** |
| --- | --- |
| TRBCRo | GTGTGGCCTTTTGGGTGTGG |
| TRBV1Fo | AATGAAACGTGAGCATCTGG |
| TRBV2Fo | GTGTCCCCATCTCTAATCAC |
| TRBV3Fo | TATGTATTGGTATAAACAGG |
| TRBV4Fo | GTCTTTGAAATGTGAACAAC |
| TRBV5Fo | GATCAAAACGAGAGGACAGC |
| TRBV6aFo | GTGTGCCCAGGATATGAACC |
| TRBV6bFo | CAGGATATGAGACATAATGC |
| TRBV7Fo | CTCAGGTGTGATCCAATTTC |
| TRBV9Fo | GAGACCTCTCTGTGTACTGG |
| TRBV10Fo | GGAATCACCCAGAGCCCAAG |
| TRBV11Fo | CCTAAGGATCGATTTTCTGC |
| TRBV12Fo | AGGTGACAGAGATGGGACAA |
| TRBV13Fo | CTATCCTATCCCTAGACACG |
| TRBV14Fo | AGATGTGACCCAATTTCTGG |
| TRBV15Fo | TCAGACTTTGAACCATAACG |
| TRBV16Fo | TATTGTGCCCCAATAAAAGG |
| TRBV17Fo | ATCCATCTTCTGGTCACATG |
| TRBV18Fo | GCAGCCCAATGAAAGGACAC |
| TRBV19Fo | TGAACAGAATTTGAACCACG |
| TRBV20Fo | TCGAGTGCCGTTCCCTGGAC |
| TRBV21Fo | GCAAAGATGGATTGTGTTCC |
| TRBV23Fo | CATTTGGTCAAAGGAAAAGG |
| TRBV24Fo | ATGCTGGAATGTTCTCAGAC |
| TRBV25Fo | CTCTGGAATGTTCTCAAACC |
| TRBV26Fo | CCCAGAATATGAATCATGTT |
| TRBV27Fo | TTGTTCTCAGAATATGMCC |
| TRBV28Fo | ATGTGTCCAGGATATGGACC |
| TRBV29Fo | TCACCATGATGTTCTGGTAC |
| TRBV30Fo | TGTGGAGGGAACATCAAACC |
| TRBV1Fi | **TCTTTCCCTACACGACGCTCTTCCGATCT**CATTGAAAACAAGACTGTGC |
| TRBV2Fi | **TCTTTCCCTACACGACGCTCTTCCGATCT**TGAAATCTCAGAGAAGTCTG |
| TRBV3Fi | **TCTTTCCCTACACGACGCTCTTCCGATCT**CTCTAAGAAATTTCTGAAGA |
| TRBV4Fi | **TCTTTCCCTACACGACGCTCTTCCGATCT**GGAGCTCATGTTTGTCTACA |
| TRBV5aFi | **TCTTTCCCTACACGACGCTCTTCCGATCT**CAGGGGCCCCAGTTTATCTT |
| TRBV5bFi | **TCTTTCCCTACACGACGCTCTTCCGATCT**GAAACARAGGAAACTTCCCT |
| TRBV6aFi | **TCTTTCCCTACACGACGCTCTTCCGATCT**GGTATCGACAAGACCCAGGC |
| TRBV6bFi | **TCTTTCCCTACACGACGCTCTTCCGATCT**TAGACAAGATCTAGGACTGG |
| TRBV7aFi | **TCTTTCCCTACACGACGCTCTTCCGATCT**TCTAATTTACTTCCAAGGCA |
| TRBV7bFi | **TCTTTCCCTACACGACGCTCTTCCGATCT**TCCCAGAGTGATGCTCAACG |
| TRBV7cFi | **TCTTTCCCTACACGACGCTCTTCCGATCT**ACTTACTTCAATTATGAAGC |
| TRBV7dFi | **TCTTTCCCTACACGACGCTCTTCCGATCT**CCAGAATGAAGCTCAACTAG |
| TRBV9Fi | **TCTTTCCCTACACGACGCTCTTCCGATCT**CTCATTCAGTATTATAATGG |
| TRBV10Fi | **TCTTTCCCTACACGACGCTCTTCCGATCT**GACATGGGCTGAGGCTGATC |
| TRBV11Fi | **TCTTTCCCTACACGACGCTCTTCCGATCT**ACTCTCAAGATCCAGCCTGC |
| TRBV12aFi | **TCTTTCCCTACACGACGCTCTTCCGATCT**TGCAGGGACTGGAATTGCTG |
| TRBV12bFi | **TCTTTCCCTACACGACGCTCTTCCGATCT**GTACAGACAGACCATGATGC |
| TRBV13Fi | **TCTTTCCCTACACGACGCTCTTCCGATCT**AAGATGCAGAGCGATAAAGG |
| TRBV14Fi | **TCTTTCCCTACACGACGCTCTTCCGATCT**AGTCTAAACAGGATGAGTCC |
| TRBV15Fi | **TCTTTCCCTACACGACGCTCTTCCGATCT**AAAGATTTTAACAATGAAGC |
| TRBV16Fi | **TCTTTCCCTACACGACGCTCTTCCGATCT**AATGTCTTTGATGAAACAGG |
| TRBV17Fi | **TCTTTCCCTACACGACGCTCTTCCGATCT**AACATTGCAGTTGATTCAGG |
| TRBV18Fi | **TCTTTCCCTACACGACGCTCTTCCGATCT**AATATCATAGATGAGTCAGG |
| TRBV19Fi | **TCTTTCCCTACACGACGCTCTTCCGATCT**TTTCAGAAAGGAGATATAGC |
| TRBV20Fi | **TCTTTCCCTACACGACGCTCTTCCGATCT**GATGGCAACTTCCAATGAGG |
| TRBV21Fi | **TCTTTCCCTACACGACGCTCTTCCGATCT**CGCTGGAAGAAGAGCTCAAG |
| TRBV23Fi | **TCTTTCCCTACACGACGCTCTTCCGATCT**GAATGAACAAGTTCTTCAAG |
| TRBV24Fi | **TCTTTCCCTACACGACGCTCTTCCGATCT**GTCAAAGATATAAACAAAGG |
| TRBV25Fi | **TCTTTCCCTACACGACGCTCTTCCGATCT**TAATTCCACAGAGAAGGGAG |
| TRBV26Fi | **TCTTTCCCTACACGACGCTCTTCCGATCT**ATTCACCTGGCACTGGGAGC |
| TRBV27Fi | **TCTTTCCCTACACGACGCTCTTCCGATCT**TGAGGTGACTGATAAGGGAG |
| TRBV28Fi | **TCTTTCCCTACACGACGCTCTTCCGATCT**AAAAGGAGATATTCCTGAGG |
| TRBV29Fi | **TCTTTCCCTACACGACGCTCTTCCGATCT**CTGGACAGAGCCTGACACTG |
| TRBV30Fi | **TCTTTCCCTACACGACGCTCTTCCGATCT**TTCTACTCCGTTGGTATTGG |
| SuperF | AATGATACGGCGACCACCGAGATCTACACTCTTTCCCTACACGACG |
| SuperR | CAAGCAGAAGACGGCATACGAGAT-**barcode**-GTGACTGGAGTTCAGACGTGTG |

*Common sequences are indicated in bold.
